# MUG-Mel2, a novel highly pigmented and well characterized NRAS mutated human melanoma cell line

**DOI:** 10.1038/s41598-017-02197-y

**Published:** 2017-05-18

**Authors:** Beate Rinner, Greta Gandolfi, Katharina Meditz, Marie-Therese Frisch, Karin Wagner, Alessia Ciarrocchi, Federica Torricelli, Raili Koivuniemi, Johanna Niklander, Bernadette Liegl-Atzwanger, Birgit Lohberger, Ellen Heitzer, Nassim Ghaffari-Tabrizi-Wizsy, Dagmar Zweytick, Iris Zalaudek

**Affiliations:** 1 Division of Biomedical Research, Medical University Graz, Graz, Austria; 2 Laboratorio di Ricerca Traslazionale Arcispedale S. Maria Nuova - IRCCS, Reggio Emilia, Italy; 3 Center for Medical Research, Medical University of Graz, Graz, Austria; 4 Division of Pharmaceutical Biosciences, Faculty of Pharmacy, University of Helsinki, Helsinki, Finland; 5 Institute of Pathology, Medical University of Graz, Graz, Austria; 6 Department of Orthopedic Surgery, Medical University of Graz, Graz, Austria; 7 Institute of Human Genetics, Medical University of Graz, Graz, Austria; 8 In SFL Chicken CAM Lab, Institute of Pathophysiology and Immunology, Medical University of Graz, Graz, Austria; 9 Institute of Molecular Biosciences, Biophysics Division, University of Graz, Graz, Austria; 10 Division of Dermatology, Graz, Medical University Graz, Graz, Austria

## Abstract

NRAS mutation in melanoma has been associated with aggressive tumor biology and poor prognosis. Although targeted therapy has been tested for NRAS mutated melanoma, response rates still appear much weaker, than in BRAF mutated melanoma. While plenty of cell lines exist, however, only few melanogenic cell lines retain their *in vivo* characteristics. In this work we present an intensively pigmented and well-characterized cell line derived from a highly aggressive NRAS mutated cutaneous melanoma, named MUG-Mel2. We present the clinical course, unique morphology, angiogenic properties, growth characteristics using *in vivo* experiments and 3D cell culture, and results of the exome gene sequencing of an intensively pigmented melanogenic cell line MUG-Mel2, derived from a cutaneous metastasis of an aggressive NRAS p. Q61R mutated melanoma. Amongst several genetic alterations, mutations in GRIN2A, CREBP, PIK3C2G, ATM, and ATR were present. These mutations, known to reinforce DNA repair problems in melanoma, might serve as potential treatment targets. The aggressive and fast growing behavior in animal models and the obtained phenotype in 3D culture reveal a perfect model for research in the field of NRAS mutated melanoma.

## Introduction

The term melanoma embraces a large spectrum of malignant melanocytic proliferations that differ with respect to their clinical morphology, epidemiology, growth pattern and, genetic alterations. Mutations in the BRAF (B-Raf proto-oncogene, serine/threonine kinase) and NRAS (neuroblastoma rat sarcoma viral oncogene) have been identified as key drivers in melanoma progression. In approximately 50% of cutaneous melanomas BRAF mutations are found, while about 25% of melanomas harbor mutations in NRAS^1^. The two alterations appear to be mutually exclusive.

NRAS mutations lead to activation of the MAPK and PI3K/Akt/mTOR pathway^2^ and are present among melanoma in various other cancers such as leukemia, lung and colorectal cancer^3^. In melanoma, the vast majority (90%) of mutations are located in codon 61, represented mostly by p. Q61K and p.Q61R mutations^4, 5^. In contrast to BRAF mutated melanoma, the clinical and molecular characteristics of NRAS mutated melanoma subtypes are less well documented. Current literature however, suggests a more aggressive nature of NRAS mutated melanomas compared to BRAF mutated or wildtype melanomas. In particular the NRAS Q61 mutation has been shown to be associated with poor outcome^6^. Besides the biological differences between NRAS and BRAF mutated melanoma, the poor outcome might also be related to a weaker response of NRAS mutated melanoma compared to BRAF mutated melanomas to current targeted therapy (i.e. vemurafenib, dabrafenib with or without MAPK inhibitors)^7^. For these reasons, the development of melanoma cell lines that retain comparable characteristics as in the *in vivo* state are crucial to search for novel treatment options in this particular subset of NRAS mutated melanomas.

To obtain the morphology *in vitro* as close as possible to the *in vivo* state we cultured the cell line in a nanofibrillar cellulose (NFC) hydrogel. When considering suitable scaffold for the 3D-culture of MUG-Mel2, some important attributes were taken into account. The material should be possible to be optimized for MUG-Mel2 due its novel status, and the scaffold should also be removable for further analysis, such as immunohistochemistry. As one of the few completely removable 3D-scaffolds available^8^, NFC was considered a good option for the study. In addition that the NFC hydrogel’s concentration can be tailored for every cell line^8^, resulting into suitable scaffold stiffness for the cell line in question, the material has also been found to be able to support organotypic growth of the cell line HepG2, indicating a promising 3D environment modelling for a neoplastic cell line^9^.

Herein, we present the clinical, biological and genetic characteristics of a peculiar melanogenic cell line - named MUG-Mel2, derived from a fresh biopsy tissue of a cutaneous metastasis of a highly aggressive NRAS p.Q61R mutated melanoma. For the genetic analysis we performed copy number variations, targeted exome sequencing and determined pathway analyzes. The unique phenotype of the melanoma - the stable brown coloring, could be preserved over all passages in cell culture, which in turn is beneficially convenient and, useful for easy detection *in vivo* experiments. Cell culture models that maintain characteristics of the *in vivo* state are crucial research models to explore novel and relevant targets for treatment.

## Results

### Cell Line Establishment

**Figure 1:**
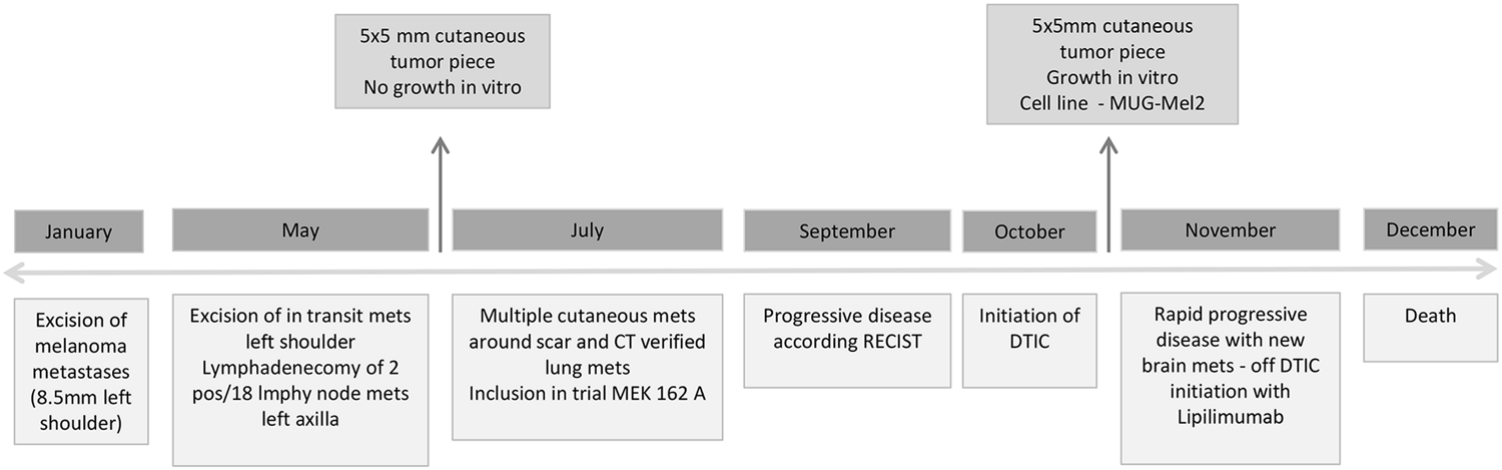
Clinical course and date of MUG-Mel2 cell line establishment.

**Figure 2:**
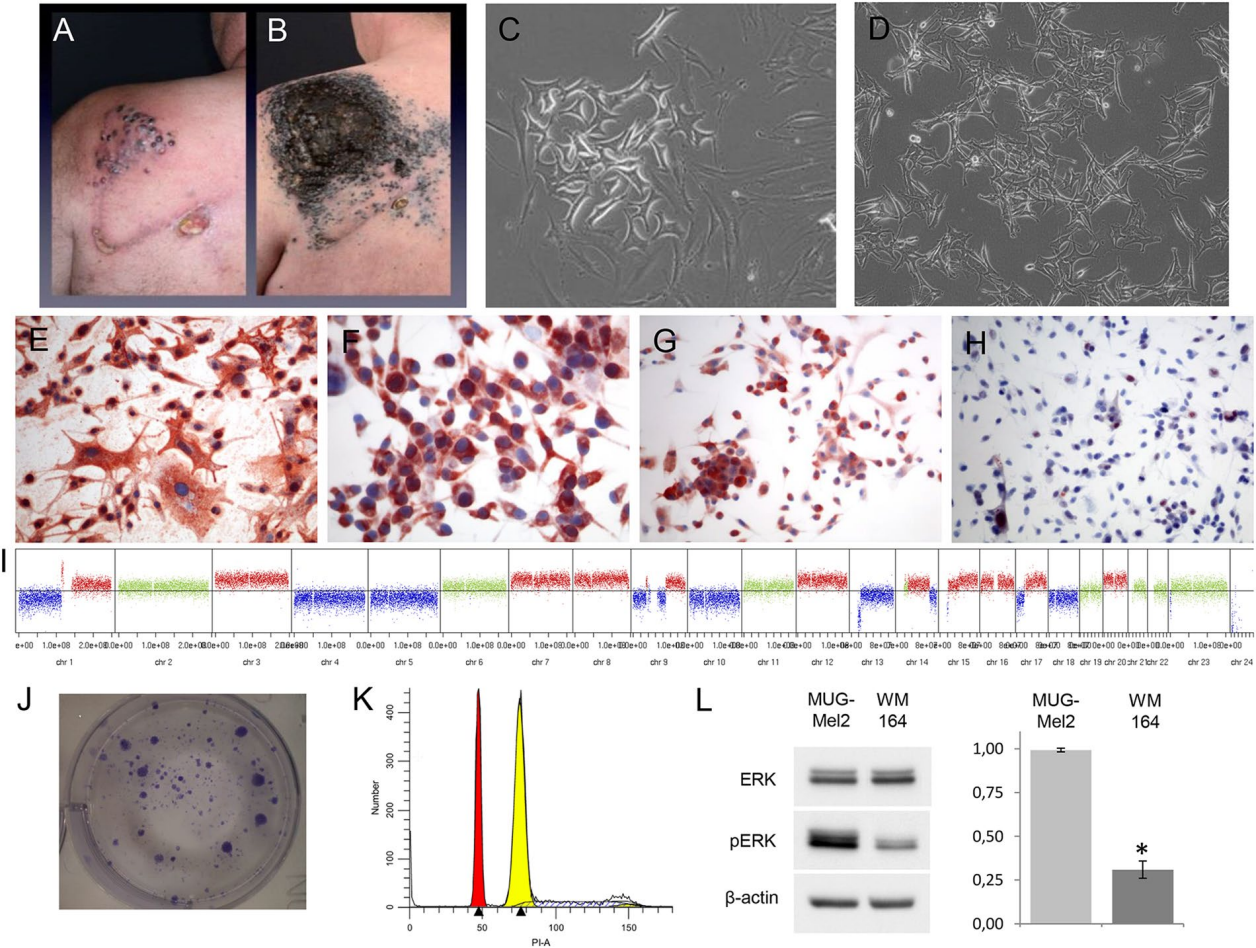
Male patient with cutaneous metastases appearing shortly after wide surgical excision of a primary melanoma on his left shoulder (**A**). Rapid progression of cutaneous metastases three months after the excision of the primary melanoma irrespective of targeted therapy (**B**), outgrowth of melanoma tumor cells surrounded by stroma cells (**C**), MUG-Mel2 cell line after ten days (**D**). Immunohistochemistry revealed in a strong HMB-45 expression (**E**), strong Melan-A expression (**F**) strong Tyrosinase expression (**G**) and a weak S-100 expression (**H**). Copy number variation profile of MUG-Mel2 passage 89. Regions with log2 ratios > 0.2 that indicate gains of chromosomal material are shown in red and those with log2 ratios < −0.2 that indicate loss of chromosomal material are shown in blue. Balanced genomic regions are depicted in green (**I**). Tumorigenicity was performed by CFU assay (**J**). DNA index was calculated by analyzing the geometric mean of MUG-Mel2 and MNC. DNA index of 1.6 was calculated, which means that the tumor cells were hyperdiploid, cell cycle was analysed by ModFit software 4.1, red peak indicats G1 peak of MNC, yellow peak G1 peak of MUG-Mel2 cells (**K**). Phosphorylation of ERK was determined by Western blot, WM164 was used as control cell line (**L**).

The MUG-Mel2 cell line was obtained from a cutaneous primary, ulcerated melanoma on the left shoulder. Clinical course time line is described in Fig. 1. Four months after wide surgical excision of the primary melanoma, the patient developed multiple cutaneous satellite metastases around the scar with progressive spread all over the body (Fig. 2A). Tumor pieces were cultured *in vitro*, no melanoma cell growth can be obtained. After three months of the treatment with MEK inhibitor (binetimib in a trial), there was no objective evidence of response as the patient continued to develop multiple cutaneous metastases (Fig. 2B). After tumor therapy, tumor pieces were given in culture, a massive growth of the cells occurred and the cell line MUG-Mel2 was established. Right from the beginning, culture revealed prominent cells with triangular dendritic morphology as typically seen in melanoma and surrounding fibroblast-like cells (Fig. 2C). After ten days, exclusively melanoma cells were further cultured (Fig. 2D). During cell line cultivation, cells were regularly cryopreserved. Although cells were passaged more than 100 times and retained in culture for more than 12 months, no significant changes in their morphology were observed. For cell line identification we used Power Plex^®^ 16, frozen primary tumor and MUG-Mel 2 passage nine and passage 60, all showed the same STR profile in all markers (Suppl Table 1). Immunostaining of MUG-Mel2 revealed high expression of HMB-45 (Fig. 2E), Melan-A (Fig. 2F), Tyrosinase (Fig. 2G), and a weak expression of S100 (Fig. 2H). Cell growth rate was determined by real-time monitoring xCELLigence and doubling time of 38.19 ± 4.09 hours was calculated. Cell size of 20.59 µm was measured by Casy cell counting.

### Genetic Characterization

In order to characterize the cell line at the genetic level, we analyzed copy numbers using low-coverage whole genome sequencing. Genome wide copy numbers inferred from the counts were observed across the genome. MUG-Mel2 showed a variety of copy number aberrations, including gains of chromosome 1p, 3, 7, 8, 9q, 12, 14, 15, 16, 17q, 20 as well as losses of chromosomes 1q, 4, 5, 9p, 10, 13, 17p and 18 (Fig. 2I), These genetic alterations are highly concordant with available copy number data for melanoma from the public Progenetix database (Suppl Figure 1). CFU assays to confirm the tumorigenicity revealed a total of 26.83 ± 4.79 clones after a period of 30 days, a representative example is presented in Fig. 2J. DNA index was calculated by analyzing the geometric mean of MUG-Mel2 78.00/ geometric mean of MNC 48.68. A DNA index of 1.6 was calculated, which means that the tumor cells were hyperdiploid. Cell cycle of spiked MNC and MUG-Mel2 analysed with two cell cycles are presented in Fig. 2K. Phosphorylation of ERK, a key player of RAS oncogenic pathway was determined by Western blot. Compared with WM 164 (BRAF mutated) cell line a significant increase of ERK phosphorylation was detected (Fig. 2L). Due to this putative activation, we checked available low-coverage whole genome sequencing data for focal amplifications of genes involved in the respective pathway. However, no focal amplifications were observed for AKT, BRAF, EGFR, KRAS, MAPK1-15, MEK, NRAS or PIK3CA (Suppl Table 2).

### *Ex ovo* CAM Assay

**Figure 3:**
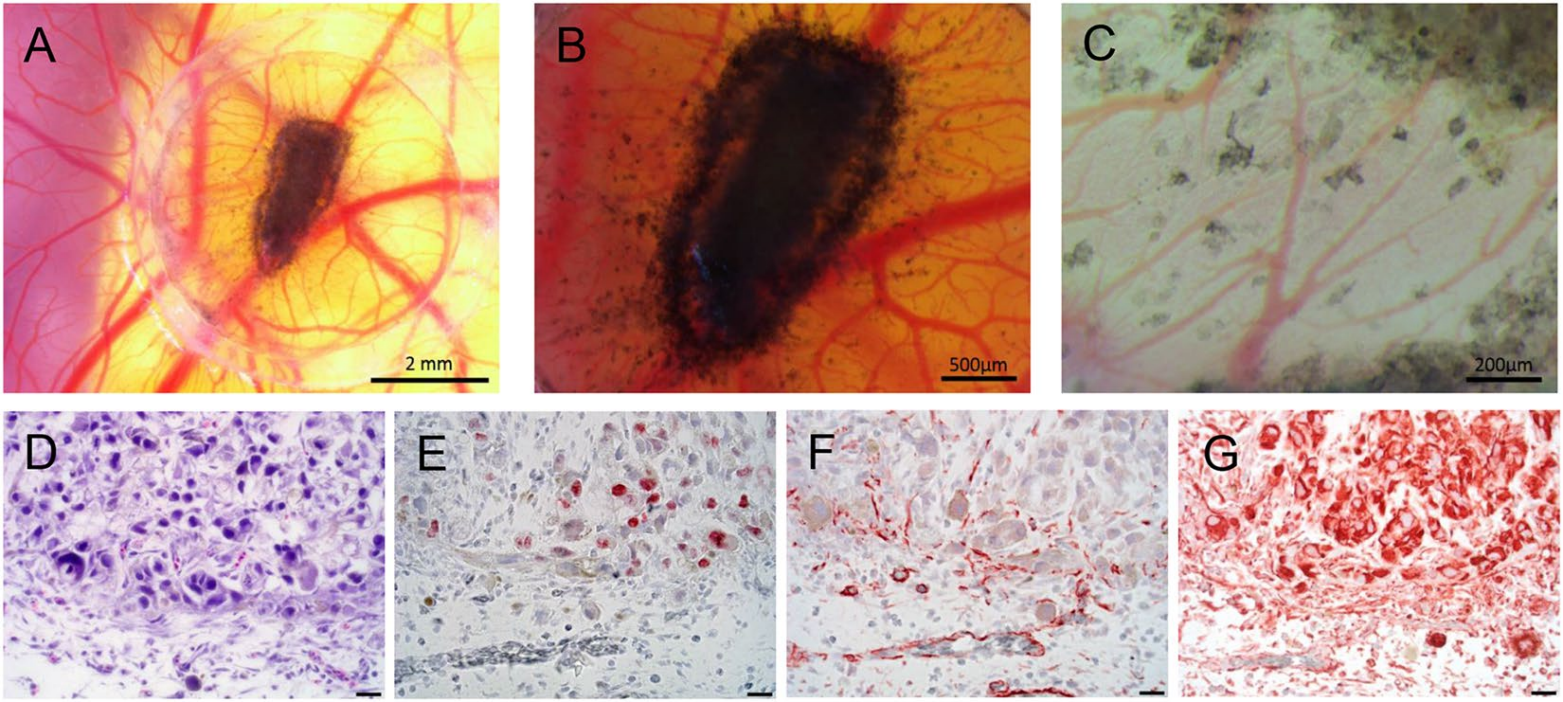
*Ex ovo* CAM assay: MUG-Mel2 onplants formed highly pigmented tumors within the silicone ring on the CAM surface after three days of incubation. Avian vessels developed radially towards the onplants (**A**: 10x magnification, **B**: 25x magnification), loco-regional metastases with solitary evading tumor cells surrounded by newly formed blood vessels were present (**C**: 63x magnification). Morphological analysis of hematoxylin/eosin stained sections revealed strong interaction of MUG-Mel2 cells with the CAM mesenchyme and invasion of tumor cells from the primary onplant site into the surrounding CAM tissue (**D**). MUG-Mel2 cells are mitotic active (Ki-67 staining) (**E**), tumor-driven neoangiogenesis was detected with anti-desmin (**F**). MUG-Mel2 cells express the tumor biomarker vimentin (**G**) (400x magnification, scale bar = 20 µm).

In the *ex ovo* CAM assay MUG-Mel2 formed intensively pigmented tumor masses within three days. Macroscopic observation revealed attraction of numerous vessels that developed radially towards the onplants and the presence of loco-regional metastases with solitary evading tumor cells surrounded by newly formed blood vessels (Fig. 3A–C). HE staining revealed that solid tumor samples were strongly connate to the CAM with outgrowth of the tumor invading avian vasculature (Fig. 3D), which can also be detected by desmin staining (Fig. 3F). Proliferating cells were detected by Ki-67 staining and were shown to be uniformely distributed throughout the xenograft (Fig. 3E). MUG-Mel2 cells also highly express vimentin, a cytoskeletal protein, which is present in several melanoma cells (Fig. 3G).

### Xenotransplants confirmed tumorigenicity of MUG-Mel2

**Figure 4:**
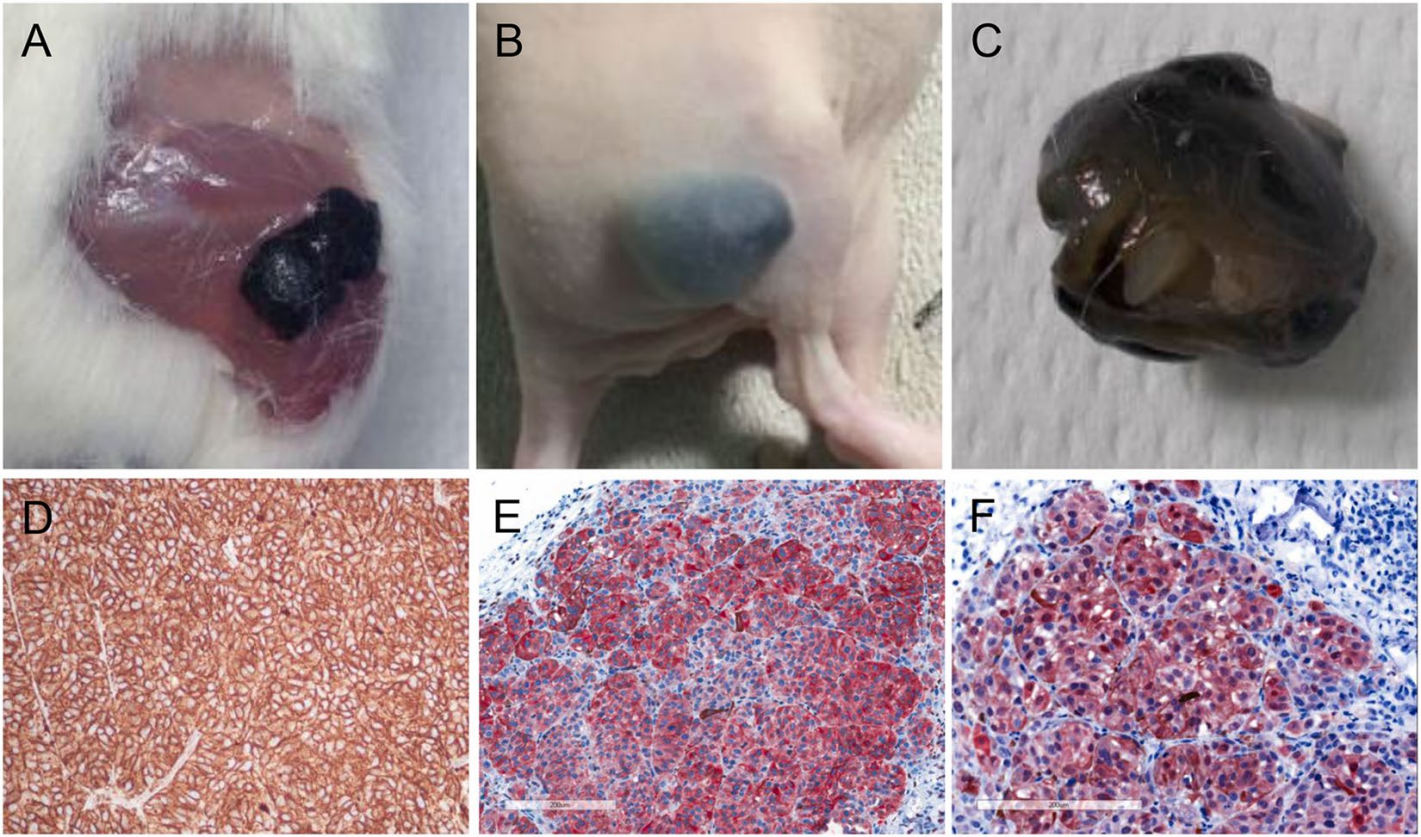
Tumorigenicity: MUG-Mel2 growing in NSG mice (**A**), black tumor was visible within 10 days (**B**). Separated tumor from nude mouse (**C**). IHC from xenotransplantations with strong HMB-45 expression (**D**), strong Melanin-A expression (**E**), and strong S-100 expression (**F**).

The melanoma cells were easily detectable because of the intense pigmentations. To determine tumorigenicity of MUG-Mel2, cells were injected first into NOD/SCID/Il −2rznull (NSG) mice. All three (3/3) xenotransplants developed black nodules after 10 days. The same evolution and growth was also detectable in all xenotransplants (5/5) in nude mice at 100% (Fig. 4A–C). To verify the melanoma growing in mice, IHC were performed, showing a strong expression of HMB-45 (Fig. 4D), strong expression of Melan-A (Fig. 4E), and a strong expression of S100 (Fig. 4F). From each mouse liver, spleen, heart, kidney and lung was examined, no metastases were found within one month. Tumor material from NSG mice was mechanically dissociated and again established in cell culture (xMUG-Mel2). Both, MUG-Mel2 and xMUG-Mel2 were seeded into 96 wells, MTS assays were performed in order to test whether the proliferation rate can be increased after *in vivo* stimulation. There were no significant differences regarding the growth behavior of MUG-Mel2 and xMUG-Mel2 (data not shown).

### MUG-Mel2 cells were able to form spheroids

**Figure 5:**
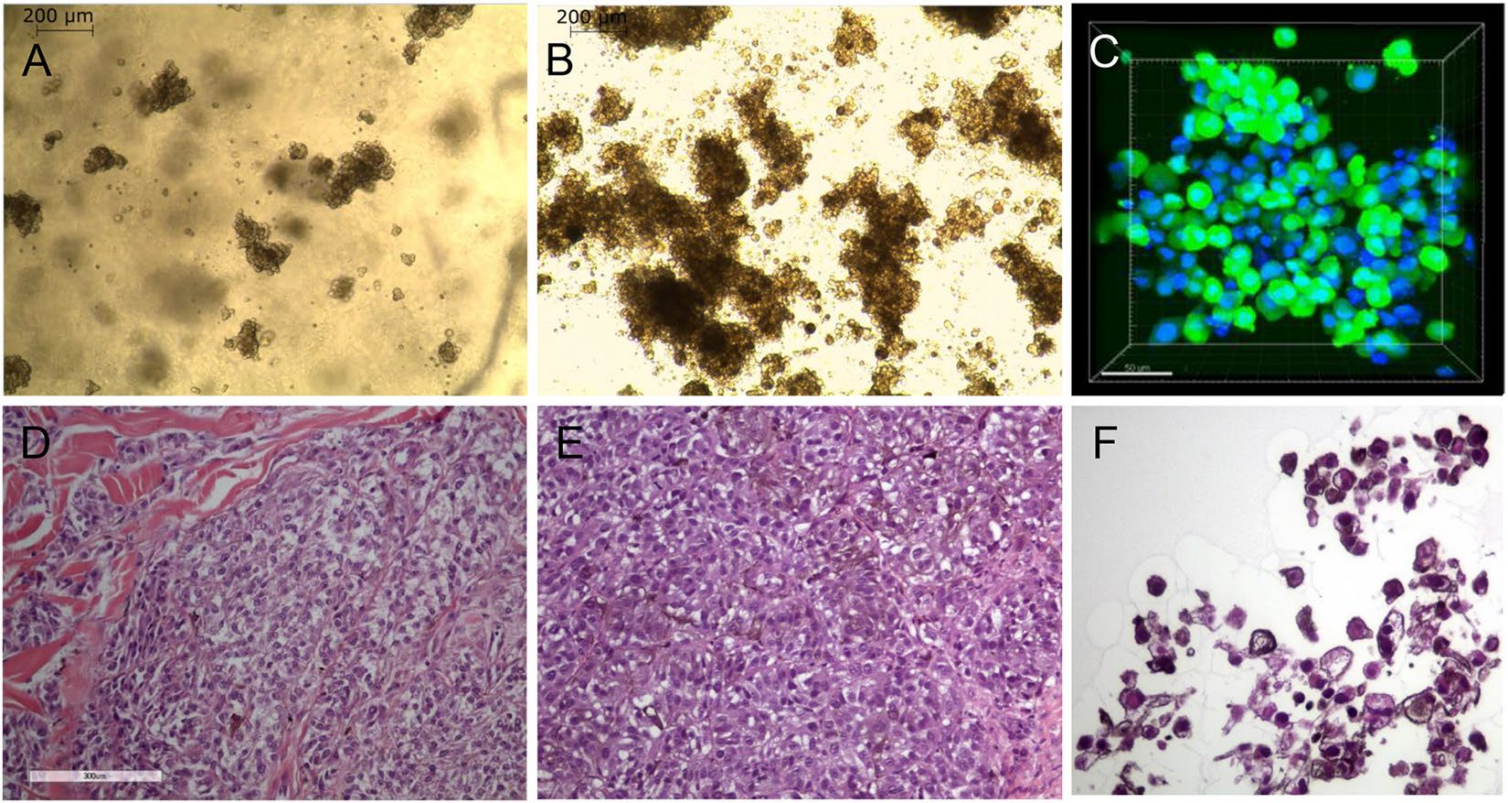
Morphology of MUG-Mel2 in 3D cultures. Cells formed spheroids in nanofibrillar cellulose (NFC) after two days in a concentration of 0.4% (**A**: 50x magnification), after five days in a concentration of 0.4% (**B**: 50x magnification); intensive brown staining was observed. Calcein staining (green) revealed the viability of the spheroids, nuclei were counterstained with DAPI (blue) (**C**). HE staining of human tumor tissue (**D**), tumor xenotransplantat (**E**), and 3D culture of MUG-Mel2 cells showed typical melanoma morphology and pigmented cytoplasm (**F**).

The ability of MUG-Mel2 to form spheroids could be achieved by cultivation in NFC scaffolds, which was tested by using three different concentrations of NFC and two cell concentrations. The morphology of the spheroids after two days and after five days is shown in Fig. 5A,B. To indicate the viability of 3D cultures, Calcein was used, while only live cells can hydrolyze the cell-permable dye; cell nuclei were counterstained with DAPI (Fig. 5C). The typical morphology of the melanoma cells has been preserved from the human tumor tissue, via the xenograft, to the 3D culture (Fig. 5A–F). Cells cultured in NFC showed a strong pigementation in the cytoplasm of melanoma cells (Fig. 5F).

### Somatic variants of the MUG-Mel2 cell line

**Figure 6:**
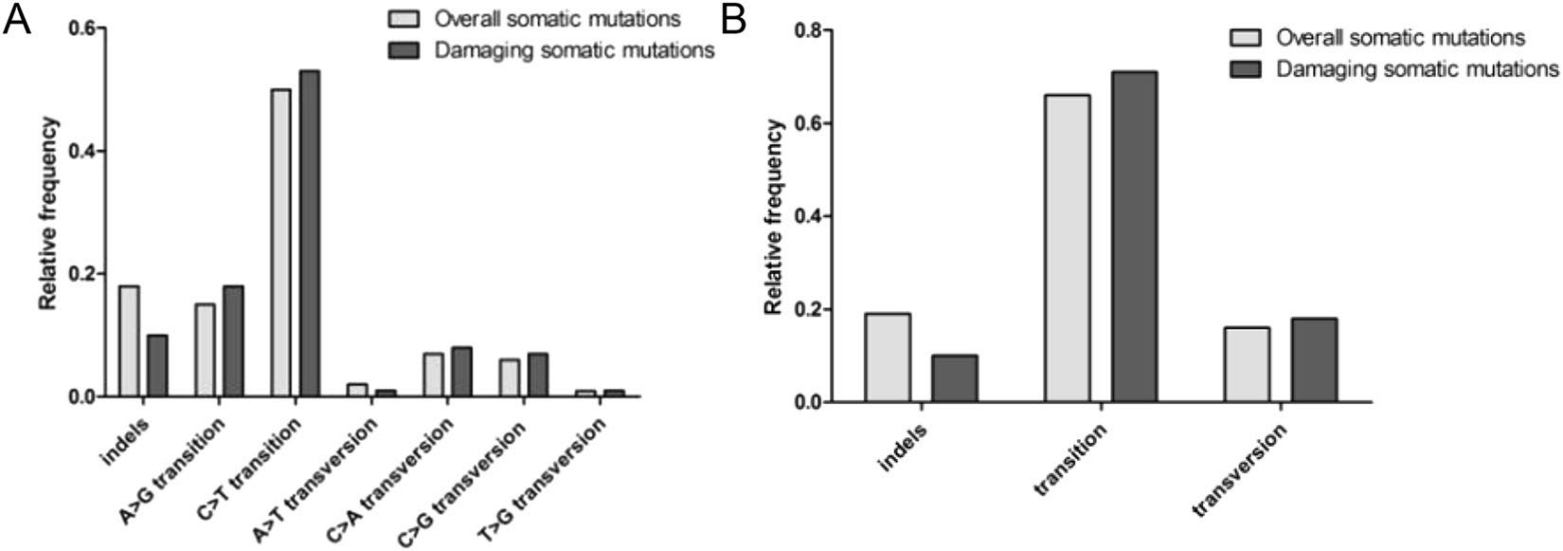
Relative frequencies of each type of nucleotide substitution (**A**) and of indels, transitions and transversions (**B**) among the overall somatic mutations and among the damaging somatic mutations detected in MUG-Mel 2 cell line.

**Figure 7:**
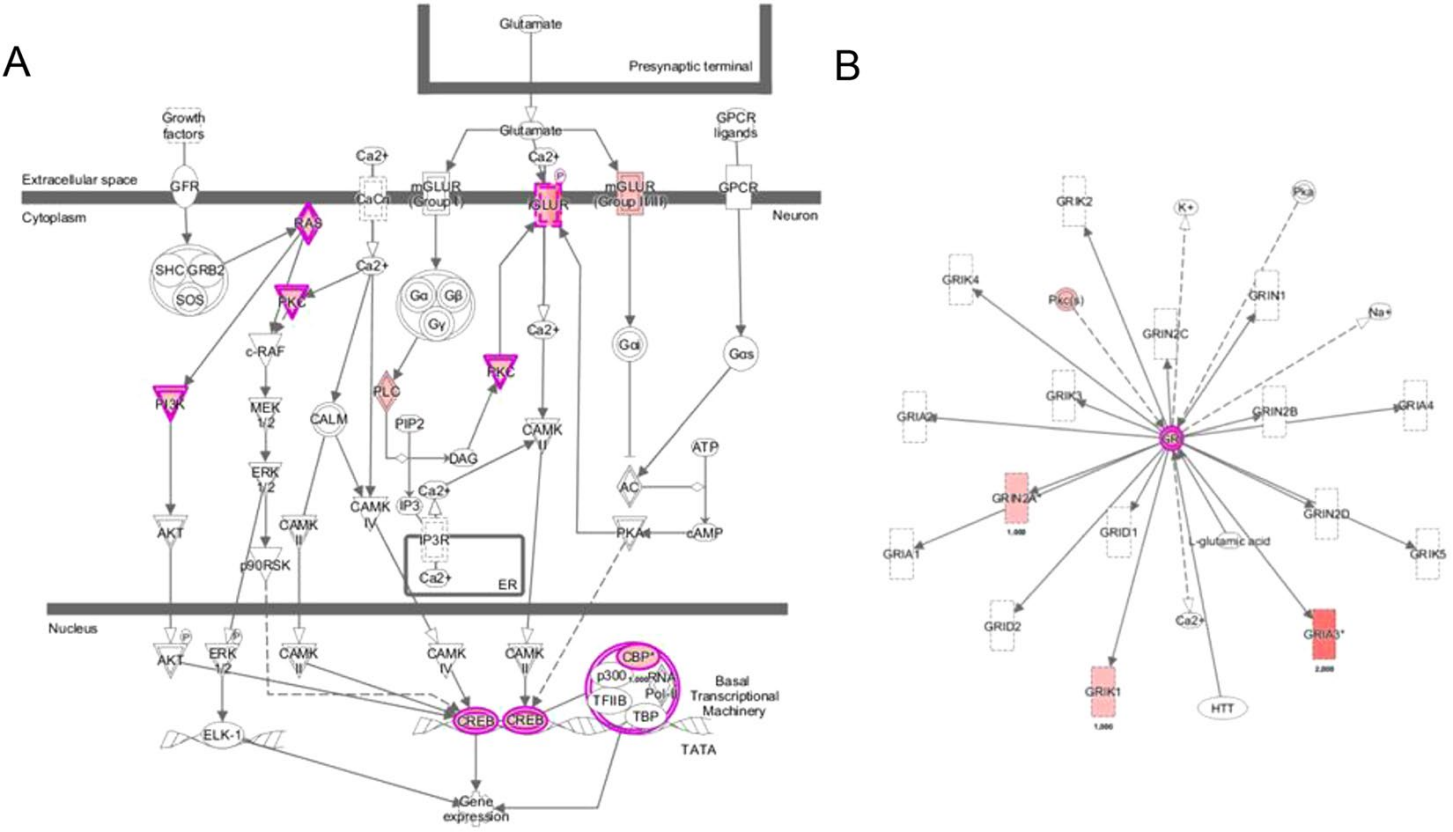
Canonical Pathway of ‘CREB Signaling in Neurons’ (IPA, p-value 3.02E-05). Color red: the darker, the more damaging mutations were found by exon sequencing. Color pink: overlap of genes within dataset of 282 damaging mutations. Color pink double bordered: shows more than one gene within a complex. Genes from the dataset involved in the pathway are: PRKDC, NRAS, TYK2, CREBBP, PIK3C2G, ATR, JAK2, PRKD3, ATM.

**Figure 8:**
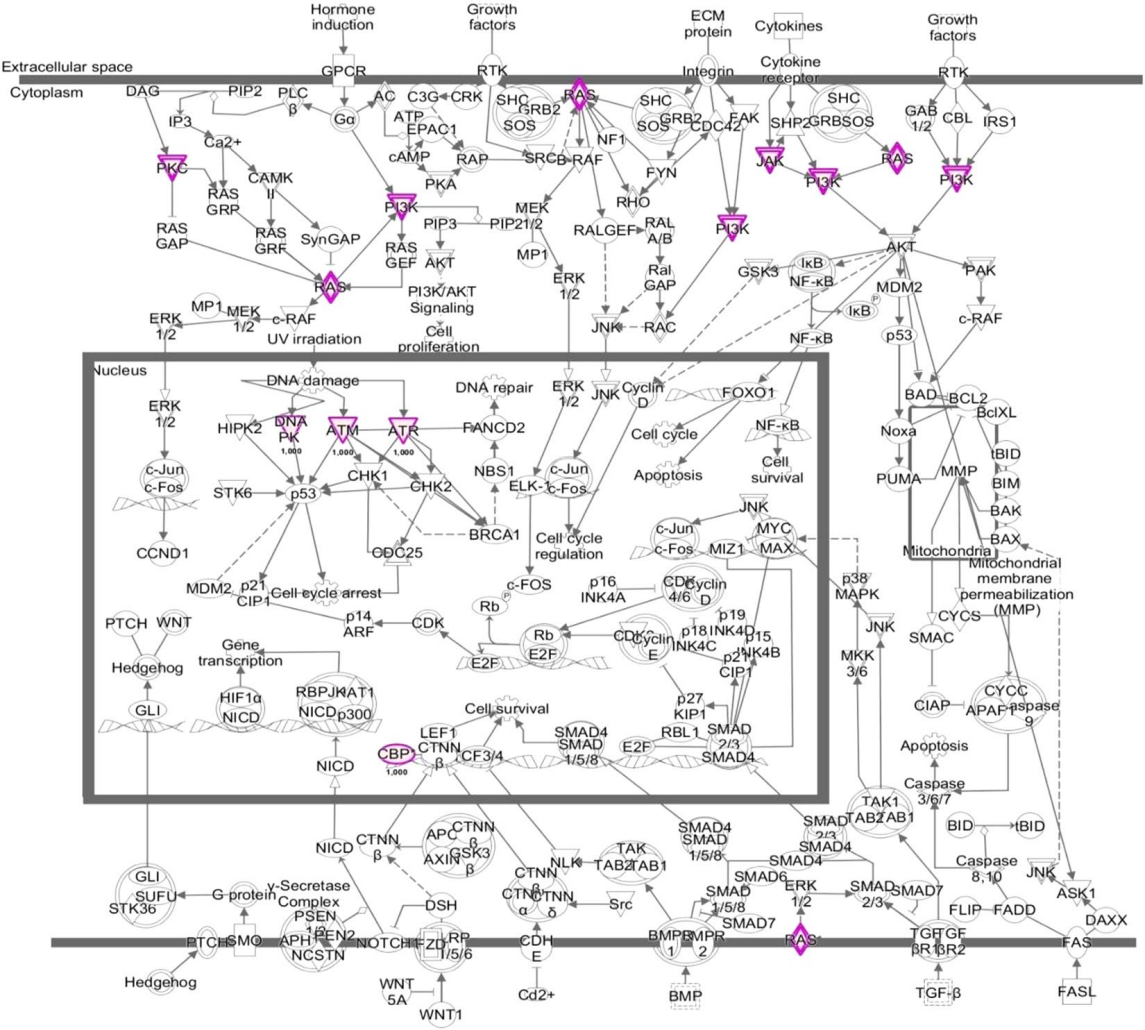
Canonical Pathway of ‘Molecular Mechanisms of Cancer’ (IPA, p-value 0.0199). Color red: the darker, the more damaging mutations were found by exon sequencing. Color pink: overlays within dataset of 282 damaging mutations. Color pink double bordered: shows more than one gene within a complex. Genes from the dataset involved in the pathway are: PRKDC, NRAS, TYK2, CREBBP, PIK3C2G, ATR, JAK2, PRKD3, ATM.

Targeted exome sequencing was used to detect somatic variants in the tumor cell line. We identified a total of 647 somatic mutations, affecting a total of 451 genes, and a mutational rate of 54.5 mutation/Mb (Suppl Tables S3 and S4). Of the overall mutations, 293 were missense, 6 nonsense (stop-gain), and 10 frameshift, while 186 were synonymous and 152 were located in UTR, introns or intergenic regions. The 309 possibly damaging mutations (including missense, nonsense and frameshift) affected a total of 229 genes (Table S3). To confirm this analysis, we also sequenced with the same approach MUG-Mel2 P9 and P60. The overall number of mutations was quite stable across the passages (309 in primary tumor, 305 in P9 and 272 in P60). As well, over 60% of mutations detected in the primary tumor were confirmed at the later passages suggesting an overall stability of the genome of these cells (Table S5). The majority of mutations were transitions (66% of overall somatic mutations and 66% of the damaging somatic mutations), with a striking prevalence of C > T transitions (50% of overall somatic mutations and 53% of the damaging somatic mutations) (Fig. 6A,B). Ingenuity Pathway Analysis of the 229 genes with at least one damaging mutation gave an insight of which pathways or biological functions are related to these genes. For better understanding, Table 1 presents a summary of the top 16 pathways resulting from the p-values, indicates the amount of mutation in genes. The most common genes in that pathway are ATM (14 times), PIK3C2G (14 times), NRAS (11 times), HLA-DRB1 (11 times), and HLA-DRB5 (11 times) which clearly indicates the importance of those mutations. These genes are among other represented in ‘CREB Signaling in Neurons’ (p-value 3.02E-05), mutated genes: GRIN2A, PLCE1, NRAS, CREBBP, PIK3C2G, GRM6, PRKD3, GRIA3, ATM, GRIK1 (Fig. 7). Looking deeper at the ATM mutation, it might be visible in the ‘Molecular Mechanisms of Cancer’ (p-value 0.0199) pathway (Fig. 8). We found a high mutational burden in the overall target (386 mutation/Mb), the genes which are listed in Table S2 with decreasing damaging mutation burden, the most highly mutated genes are KIR2DL1, KIR2DL3, AQP7, HLA-DRB5, SAA2, HLA-A, KIR3DL1, HLA-DPB1, HLA-C, CCL22 are involved in immune response, whereas mutated genes are enriched for metabolic, immunorelated and cancer-related pathways.

**Table 1:** Export of Ingenuity Pathway Analysis of 229 genes with at least one damaging mutation showing the top 16 most significant Canonical Pathways across the entire dataset.

**Ingenuity Canonical Pathways**	**(p-value)**	**Ratio**	**No overlap with dataset**	**Molecules**
Crosstalk between Dendritic Cells and Natural Killer Cells	**4,7863E-07**	1,01E-01	80/89 (90%)	KIR2DL1/KIR2DL3,KIR3DL1,HLA-DRB1,HLA-A,HLA-C,KIR3DL2,KIR2DL4,MICA,HLA-DRB5
IL-4 Signaling	**4,2658E-06**	9,20E-02	79/87 (91%)	NRAS,HLA-DRB1,TYK2,PIK3C2G,HLA-DQB1,JAK2,HLA-DRB5,ATM
Synaptic Long Term Potentiation	**5,88844E-06**	7,50E-02	111/120 (93%)	GRIN2A,PLCE1,NRAS,CREBBP,GRM6,CACNA1C,PRKD3,GRIA3,GRIN3A
Dendritic Cell Maturation	**6,76083E-06**	5,79E-02	179/190 (94%)	PLCE1,HLA-DRB1,HLA-A,HLA-C,CREBBP,PIK3C2G,HLA-DQB1,JAK2,FCGR3A/FCGR3B,HLA-DRB5,ATM
Natural Killer Cell Signaling	**6,76083E-06**	7,38E-02	113/122 (93%)	KIR2DL1/KIR2DL3,KIR3DL1,NRAS,PIK3C2G,KIR3DL2,KIR2DL4,PRKD3,FCGR3A/FCGR3B,ATM
Autoimmune Thyroid Disease Signaling	**1,04713E-05**	1,28E-01	41/47 (87%)	HLA-DRB1,HLA-A,HLA-C,HLA-DQB1,HLA-DRB5,TG
Graft-versus-Host Disease Signaling	**1,1749E-05**	1,25E-01	42/48 (88%)	KIR2DL1/KIR2DL3,HLA-DRB1,HLA-A,HLA-C,HLA-DQB1,HLA-DRB5
Amyotrophic Lateral Sclerosis Signaling	**2,63027E-05**	7,21E-02	103/111 (93%)	GRIN2A,PIK3C2G,CACNA1C,NEFH,GRIA3,ATM,GRIK1,GRIN3A
**CREB Signaling in Neurons**	**3,01995E-05**	**5,43E-02**	**174/184 (95%)**	**GRIN2A,PLCE1,NRAS,CREBBP,PIK3C2G,GRM6,PRKD3,GRIA3,ATM,GRIK1**
Neuropathic Pain Signaling In Dorsal Horn Neurons	**3,16228E-05**	7,02E-02	106/114 (93%)	GRIN2A,PLCE1,PIK3C2G,GRM6,PRKD3,GRIA3,ATM,GRIN3A
Glutamate Receptor Signaling	**3,23594E-05**	1,05E-01	51/57 (89%)	GRIN2A,GRM6,SLC1A1,GRIA3,GRIK1,GRIN3A
Antigen Presentation Pathway	**4,46684E-05**	1,35E-01	32/37 (86%)	HLA-DRB1,HLA-A,HLA-C,HLA-DPB1,HLA-DRB5
Production of Nitric Oxide and Reactive Oxygen Species in Macrophages	**4,57088E-05**	5,18E-02	183/193 (95%)	NCF1,APOB,MPO,TYK2,CREBBP,PIK3C2G,NCF4,JAK2,PRKD3,ATM
CNTF Signaling	**5,7544E-05**	9,52E-02	57/63 (90%)	LIFR,NRAS,TYK2,PIK3C2G,JAK2,ATM
Virus Entry via Endocytic Pathways	**0,000114815**	6,86E-02	95/102 (93%)	NRAS,HLA-A,HLA-C,CLTCL1,PIK3C2G,PRKD3,ATM
Mouse Embryonic Stem Cell Pluripotency	**0,000144544**	6,60E-02	99/106 (93%)	LIFR,NRAS,TYK2,CREBBP,PIK3C2G,JAK2,ATM

The significant values for the canonical pathways are calculated by Fisher’s exact test right-tailed. The ratio is calculated by the number of genes from the analysis list (229) in the given pathway, divided by the total number of genes that make up that pathway. All molecules involved are shown in the last column.

## Discussion

MUG-Mel2 is a new established cell line, characterized by mutations among others in NRAS p.Q61R, GRIN2A and DNA damaged genes. The peculiarity of the cell line is that it was cultured from a highly aggressive cutaneous metastasis, which did not respond either to targeted, immuno- or cytotoxic therapy despite revealing a mutation in NRAS p.Q61R.

The cell line exhibits well known melanoma markers such as Melan-A, S-100, Tyrosinase, and HMB-45. Remarkably, the markers and the peculiar cell morphology of heavily pigmented dendritic cells were preserved even after long-term culture conditions in 2D as well as in 3D culture. 3D and co-culture systems mimic the *in vivo* state more accurately, and enable the preservation of the original tissue’s functionality. We tested the 3D growth ability of MUG-Mel2 to see if cells are able to form spheroids and if the intense stable pigmentation of cells can be reflected. The wood based NFC hydrogel presents an optimal matrix for the cells, to maintain their natural behavior and to increase the deep brown staining (see Fig. 5A–C and F).

Incidence of melanoma has been increasing in recent years, therapies for BRAF mutated melanoma currently exist however, a considerable part of melanoma can still not be treated despite intensive research. Therefore the detection of new treatment targets and therapies is urgently needed. The Ras/Raf/MEK/ERK pathway, involved in the control of growth signals, cell survival and invasion play a major role in melanomas. Melanomas are known to harbour activating mutations in RAS and/or BRAF suggesting that the downstream effector Extracellular signal-regulated kinase (ERK) may be playing a major role in the oncogenic behavior of these tumors^10^. Activation of ERK1/2 was observed by immunohistochemistry in 54% of primary and 33% of metastastic melanomas, respectively^11^, MUG-Mel2 presents a high activated (phosphorylated) ERK1/2, significant more activated compared to WM 164, a BRAF mutated cell line. In 1970 Lee and Merrill discovered that melanomas are heterogeneous and that different clinical appearance is also associated with different distributions by site and age with different outcomes^12^. Melanomas with NRAS mutation account for approximately 25% of all melanomas, most commonly mutations are observed in codons 12, 13 and 61 which leads to aberrant cell proliferation^13^, metastasis^14^, and chemo-resistance^15, 16^. Many other cancer types also show NRAS mutation with an important clinical impact on the overall population in view of tumor incidence, mortality and survival^17^. NRAS is a frequently UV-mutated oncogene, generally occurring in melanoma of the skin in UV exposed areas, which is proven by characteristic base changes in the DNA (C > T transition). This common change is also seen in MUG-Mel2, which demonstrates a high portion of C > T transitions (>50%). We were able to analyze a variety of mutations with the targeted exome sequencing analysis. For a better overview the mutations were summarized in damaging mutations and the amount of mutations was evaluated with the ingenuity canonical pathway analysis. MUG-Mel2 presents among other mutated genes involved in DNA damage. DDR (DNA damage response) signaling pathway is the central regulation network in response to DNA damage, key proteins are ATM and ATR kinases, both are activated upon DNA damage and DNA replication stress^18^. ATM (ataxia telangiectasia mutated) or ATR (ataxia telangiectasia and Rad3-related protein) one of the DNA-damage checkpoint pathways, depending on generating double-stranded-break (DSB) or single-stranded-break DNA (ssDNA)^19^, revealed an important issue for cancer development. Interestingly ATM, located on chromosome 11, showed a balance CNV profile of ATM gene, whereas one non synonymous mutation (ATM p.T373K) was found. ATR located on chromosome 3, best known for its role in the activation of checkpoint kinase 1 (ChK1)^20^, presented a gain in MUG-Mel2, and additionally one non synonymous mutation (ATR p.K764E). ATR is essential for cell survival, knocking out of ATR in mice leads to embryonic death whereas in contrast ATM is not essential for cell survival^20^. So far no inhibitors of ATR have yet been developed. However, Chk1 and Chk2 are critical kinases downstream of both. ATM and ATR play roles in cell cycle arrest, DNA repair or apoptosis, and are valuable for further investigation. Wei *et al*. performed exome sequencing on 14 metastatic melanomas and found among others GRIN2A mutation^21^. The presence of glutamate receptor GRIN2A, which encodes N-methyl-(D)-aspartic acid (NMD) was also confirmed in other high-throughput sequencing studies^22^. Mutation of glutamate receptor (mGluRs), GRMR3 (glutamate metapotropic receptor 3) and PLCB4, an enzyme that catalyzes the formation of IP3 (inositol triphosphate) and DAG (diacylglycerol) downstream of mGluRs, seems to play an important role in melanoma^21^. While there is a long list of genes mutated in melanoma, we want to underline and emphasize mutations belonging to glutamate receptors and related pathways. GRIN2A, found on chromosome 16p13.2, bears the agonist binding side for glutamate. We observed two mutations (p.E962K and p.G1243G) in MUG-Mel2. Wei *et al*. discovered GRIN2A mutations in 6 of 14 melanoma samples, and PLCB4 (4 from 14 melanoma samples bearing a mutation). However, so far the tumorigenic effect of GRIN2A and PLCB4 is unclear. MUG-Mel2 presents one non synonymous mutation in PLCB4 and one non synonymous mutation in GRM3. GRM3, a metabolic glutamate receptor also activated by glutamate is mutated in 16% (13 of 80 tumors) of melanoma cases^23^. Prickett *et al*. showed that GRM3 promotes the proliferation and migration behavior of melanoma cells and mutated GRM3 clones are more susceptible to MEK inhibitors, AZD-6244^23^. Furthermore two GRM3 mutated cell lines exhibited anchorage-independent growth and increased migration mediated by MAPK signaling^24^. Further studies will need to address whether mutations of GRM3 or GRIN2A are the reason for tumorigenicity or aggressiveness in melanoma cells.

To conclude, the newly established cell line MUG-Mel2 presented, due to its phenotypically and genotypically precise characterization, aggressive and fast growing behavior in mouse and chick models. In particular, with the high pigmentation the cell line is a promising new model for innovative NRAS therapy options.

## Methods

### Patient History

The cell line was obtained from a fresh specimen of a cutaneous metastasis of a 48-year-old male patient with a cutaneous primary, ulcerated melanoma (8.5 mm thickness) on the left shoulder. Sentinel node biopsy of the left axilla revealed a positive node and subsequent elective lymph node dissection revealed additional two positive out of 18 excised nodes. Only four months after wide surgical excision of the primary melanoma, the patient developed multiple cutaneous satellite metastases around the scar with progressive spread all over the body (Fig. 2A). In addition multiple lung metastases were detected. Genetic analysis of the primary tumor and one cutaneous metastasis presented a mutation in NRAS p. Q61R. No mutations in BRAF or c-kit were detected. Based on the genetic analysis, treatment with a MEK inhibitor was initiated. After three months of the treatment, there was no objective evidence of response as the patient continued to develop multiple cutaneous metastases. Radiological staging moreover showed a rapid progression with new brain, liver and lung metastases. The treatment with MEK inhibitor was discontinued and he received two cycles of dacarbazine without any clinical benefit. At month one, after the diagnosis of the primary melanoma and a rapid progress, a last attempt to treat with CTL4-antibody ipilimumab was made. However, the patient died from brain metastases shortly after first administration of ipilimumab.

### Cell Culture

The tumor tissue (cutaneous metastasis) was obtained immediately after surgery, followed by mechanical disaggregation of the tumor tissue into approximately 1–2 mm^3^ pieces. Cells were cultured in RPMI (Life Technologies, Carlsbad, CA) containing 10% fetal bovine serum (FBS, Biochrom AG, Berlin, Germany), 2 mM L-glutamine (Life Technologies), and 1% penicillin/streptomycin (Pen/Strep, Life Technologies). The melanoma cells, named MUG-Mel2, grew at a pH of 7.4. Cells were grown to 80% confluence and detached from the flasks with Accutase (Sigma Aldrich, Vienna, Austria). Incubation of all cells was carried out at 37 °C in a humidified atmosphere of 5% CO_2_. All cells were periodically checked for mycoplasma by PCR. The study was approved by the local ethics committee board of the Medical University of Graz (vote #18–192ex06/07; valid until 17.04.2016) and the patient gave written informed consent for the study specific procedure. We confirm that all experiments were performed in accordance with relevant guidelines and regulations.

### MTS Assay and xCELLigence

10,000 MUG-Mel2 cells per well and 10,000 cells isolated from the xenotransplanted NSG mice were seeded into 96 well microtiter plates and the Cell Titer 96 Aqueous Non-Radioactive Cell Proliferation Assay Kit (Promega, Madison, WI) was performed according to manufacturer’s recommendations. The incubation time was 24, 48 and 72 hours, respectively. Culture medium was used as background value. The viability was determined colorimetrically by absorbance at 490 nm.

Cell proliferation was measured using the xCELLigence Real-Time Cellular Analysis system (OLS OMNI Life Science Bremen, Germany). Briefly, the background impedance was measured following the addition of 100 μL of medium to the 16-well E-plates (OLS). Cell suspensions containing various amount of cells (7.5 × 10^3^, 1 × 10^4^, 2 × 10^4^, 2. × 10^4^ cells) in 100 μL medium were seeded into the wells according to the instructions in the user’s manual. The different amounts of cells were performed in quadruplicates. Proliferation was monitored every twenty minutes. Impedance changes expressed as cell index (CI) were automatically calculated and correlated with cell growth over a period of 100 h using the RTCA software (OLS). Doubling time was calculated from 2. × 10^4^ cells.

### Immunohistochemistry

Immunostaining was performed using the Envision Plus detection system (Dako, Carpinteria, CA) and the Ultra-Vision LP detection system (Thermo Scientific, Fremont, USA) according to the manufacturer’s instructions. All specimens from the patient as well as the cell culture were stained with antibodies against Melan-A, S-100 protein, Tyrosinase, HMB-45. For CAM assay xenografts, anti-Ki-67 was used for the analysis of mitotic active cells (clone MIB-1), Anti-Desmin to detect newly formed vessels. All antibodies were obtained from DAKO. Spheroid staining: cell nucleoli were stained with Hoechst and proliferation with Ki-67 (eBioscience, San Diego, CA). Appropriate positive and negative controls were included.

### Cell Cycle

Cells were harvested by trypsinization and fixed with 70% ice-cold ethanol for 10 min at 4 °C. After washing with PBS, the cell pellet was re-suspended in PI-staining buffer (50 µl/ml PI, RNAse A, Beckman Coulter, Brea, CA) and incubated for 15 min at 37 °C. Cells were spiked with mononuclear cells (MNC), positive diploid population control and then analyzed by FACS LSRII (BD Biosciences). A minimum of 10,000 events per sample was acquired and data were determined by using FACS DIVA software (BD Biosciences). DNA index was calculated by geometric mean M2 (MUG-Mel2)/geometric mean M1 (MNC).

### Cell Forming Units (CFUs)

Two six well plates (Corning Inc., Corning, NY) were seeded with 400 cells per well and incubated at 37 °C and 5% CO_2_ for 30 days for colony formation. After the incubation time, melanoma cells were washed with PBS, fixed with 4% formaldehyde and stained with 1 mg/ml Crystal Violet, after washing with PBS, colonies were counted and mean value was formed.

### 3D cell Culturing with Nanofibrillar Cellulose Hydrogel

Sterile GrowDex™ nanofibrillar cellulose (NFC) hydrogel was obtained from UPM-Kymmene Corporation, Finland. The NFC concentration of the hydrogel was 1.55 wt%. To determine the optimal conditions for 3D cell culturing, three concentrations (0.4%; 0.7% and 1% wt/v) of NFC hydrogel, as well as two cell-seeding concentrations (50,000 and 100,000 cells per 100 ul of NFC / 96-well) were tested. MUG-Mel2 spheroids grown in 0.4% NFC hydrogel were collected at day three for live calcein staining. Prior to collection, NFC hydrogel was digested from the cell culture by cellulase enzyme (VTT, Finland) for 24 h at 37 °C using 300 µg of cellulose per 1 mg of NFC. Spheroids were washed three times with 1xDPBS, suspended in staining solution containing Calcein-AM 1:500 (Cellstain double staining kit; Sigma Aldrich) and 50 µg/ml DAPI (Invitrogen, Carlsbad, CA) for 15 min at 37 °C, and subsequently mounted using Prolong Diamond Antifade Mountant (Life Technologies). Fluorescent images were taken with the Leica TCS SP5 microscope using HCX PL APO 20x/0.7 objective. Imaris 8.4.1 software was used for image acquisition.

### *Ex ovo* CAM Assay

Fertilized Lohmann white classic chicken eggs from local hatchery were incubated at 37.6 °C and 70% humidity. The egg shell was cracked on day three of chick development and the embryo decanted to a sterile dish. On day 10, 10E6 MUG-Mel2 cells/xenograft (n = 12) were re-suspended in 15 μl PBS and 5 μl Matrigel matrix (BD, Biosciences) and grafted in the center of a 5 mm silicon ring on the surface of the chorionallantoic membrane (CAM). Xenografts were photographed using a stereo-microscope (Olympus SZX16) and excised with the surrounding CAM five days after seeding, fixed in 4% paraformaldehyde and paraffin embedded. For subsequent analyses haematoxilin/eosin and immunostaining of 5 μm sections were performed to determine the tumor cell morphology.

### Tumorigenicity

2 × 10^6^ MUG-Mel2 from passage 23 were re-suspended in 200 µl serum free medium and Matrigel (Corning) (1:1) and were subcutaneously injected into the right flank of three six-week-old female NOD/SCID/IL-2rγnull (NSG-) mice (Charles River Laboratories, Sulzfeld, Germany). Five NU-Foxn1-nu (Charles River Laboratories), eight-weeks-old, female mice were xenotransplanted with 2 × 10^6^ MUG-Mel2/100 µl serum free medium without Matrigel at passage 60. The cell suspension was inoculated subcutaneously into the right flank of the mice. The mice were observed daily. All animal work was done in accordance with a protocol approved by the institutional animal care and use committee at the Austrian Federal Ministry for Science and Research (BMWF) (vote 66.010/0160-II/3b/2012).

### Cell Line Identification Power Plex^®^ 16 system

Frozen tumor tissue was dissected into small pieces and re-suspended in 180 μl ATL buffer from Qiagen (Vienna, Austria). The cell pellet (1 × 10^6^) from MUG-Mel2 (passage 9) was re-suspended in 200 μl PBS, 20 μl proteinase K. Afterwards, 200 μl AL buffer from Qiagen was added. DNA preparations were carried out using the QIAamp DNA Mini kit (Qiagen) according to the manufacturer’s instructions. After normalizing the DNA, 0.7 ng DNA was amplified using Power Plex 16 System (Promega) in a 10 μl reaction. The product (1 μl) was mixed with Hi-Di formamide (Applied Biosystems Inc., Foster City, CA) and Internal Lane Standard (ILS600), denatured and fractionated on an ABI 3730 genetic analyzer. Resulting data were processed and evaluated using ABI Genemapper 4.0.

### Copy Number Profiling

Genome wide copy number aberrations (CNA) were established using low-coverage whole genome sequencing. Shotgun libraries were prepared using the TruSeq DNA LT Sample preparation Kit (Illumina, San Diego, CA) with slight modifications to the manufacturer’s protocol. Briefly, 380 ng, 144 ng and 360 ng input DNA from MUG-Mel2 were fragmented in 130 µl using the Covaris System (Covaris, Woburn, MA, USA). After concentrating the volume to 50 µl end repair, A-tailing and adapter ligation were performed following the manufacturer’s instructions. For selective amplification of the library fragments that have adapter molecules on both ends we used 15 PCR cycles for the higher concentrated samples, i.e. primary melanoma tumor from the skin, MBM tissue resulting MUG-Mel2 cell line. Libraries were quality checked on an Agilent Bioanalyzer using a DNA 7500 Chip (Agilent Technologies, Santa Clara, CA) and quantified using qPCR with a commercially available PhiX library (Illumina, San Diego, CA) as a standard. Libraries were pooled equimolarily and sequenced on an Illumina MiSeq in a 150 bp single read run. On completion of the run data were base called demultiplexed on the instrument (provided as Illumina).

### Targeted Exome Sequencing

Targeted exome sequencing of MUG-Mel2 cell line was performed using the TruSight One sequencing panel (Illumina), which targets 11,946,514-bp exon regions in 4,813 genes. Libraries were obtained following manufacturer´s instructions and sequenced using the MiSeq next-generation sequencer (Illumina). At least 96% of the target had a depth of coverage >20X, with a mean depth of coverage of 105X. The extracted variants were annotated using the Variant Studio software (Illumina). Variants with a reported frequency < 1% according to the 1000 Genome project annotation were considered as somatic variants.

Pathway analysis were performed with Ingenuity Pathway Analysis, IPA-version 2781452; Build: ‘ing_jakku’ (QIAGEN, Ingenuity Systems; www.ingenuity.com). A core analysis was performed to get an insight on which pathways or functional groups these genes are related to. IPA setting was filtered on human molecules and/or relationships as well as melanoma cell lines such as M14 or LOX IMVI or UACC-257 or Melanoma Cell Lines not otherwise specified or other Melanoma Cell Lines or SK-MEL-2 or SK-MEL-5 or UACC-62 or A375 or SK-MEL-28 or MALME-3M.

### Western blot analysis

For immunoblotting, whole cell protein extracts from MUG-Mel2 and WM164 were prepared with lysis buffer (50 mM Tris-HCl pH 7.4, 150 mM NaCl, 1 mM NaF, 1 mM EDTA, 1% NP-40, 1 mM Na3 VO4, and protease inhibitor cocktail (P8340; Sigma Aldrich), subjected to SDS-PAGE and blotted onto Amersham™ Protran™ Premium 0.45 µM nitrocellulose membrane (GE healthcare Life science, Little Chalfont, UK). All steps were performed on ice. Protein concentration was determined with the Pierce BCA Protein Assay Kit (Thermo Fisher Scientific) according to the manufacturer’s protocol. Primary antibodies against p44/42 MAPK (ERK 1/2), phospho-p44/42 MAPK (ERK 1/2), and β-actin were purchased from Cell Signaling Technology (Cambridge, UK). Blots were developed using a horseradish peroxidase- conjugated secondary antibody (Dako, Jena, Germany) at room temperature for 1 h and the Amersham™ ECL™ prime western blotting detection reagent (GE Healthcare), in accordance with the manufacturer’s protocol. Chemiluminescence signals were detected with the ChemiDocTouch Imaging System (BioRad Laboratories Inc., Herkules, CA) and images were processed with the ImageLab 5.2 Software (BioRad Laboratories Inc.).

## Electronic supplementary material

Supplementary Information
