# Supplementary material for: MUG-Mel2, a novel highly pigmented and well characterized NRAS mutated human melanoma cell line
Source: Sci Rep. 2017 May 18;7:2098. doi: 10.1038/s41598-017-02197-y (PMC5437015; doi:10.1038/s41598-017-02197-y)
Supplement: Supplementary file 1 — Supplementary Information [file 41598_2017_2197_MOESM1_ESM.pdf]

SREP-17-05070

MUG-Mel2, a novel highly pigmented and well characterized NRAS mutated human melanoma cell line

Beate Rinner[1*#], Greta Gandolfi[2*], Katharina Meditz[1], Marie-Therese Frisch[1], Karin Wagner[3], Alessia Ciarrocchi[2], Federica Torricelli[2], Raili Koivuniemi[4], Johanna Niklander[4], Bernadette Liegl-Atzwanger[5], Birgit Lohberger[6], Ellen Heitzer[7], Nassim Ghaffari-Tabrizi-Wizsy[8], Dagmar Zweytick[9], Iris Zalaudek[10]

[1] Division of Biomedical Research. Medical University Graz. Graz. Austria

[2] Laboratorio di Ricerca Traslazionale Arcispedale S. Maria Nuova - IRCCS. Reggio Emilia. Italy

[3] Center for Medical Research. Medical University of Graz. Graz. Austria

[4] Institute of Biomedicine/Biochemistry and Developmental Biology. University of Helsinki. Helsinki. Finland

[5] Institute of Pathology. Medical University of Graz. Graz. Austria

[6]Department of Orthopedic Surgery. Medical University of Graz. Graz. Austria

[7]Institute of Human Genetics. Medical University of Graz. Graz. Austria

[8]In SFL Chicken CAM Lab. Institute of Pathophysiology and Immunology. Medical University of Graz. Graz. Austria

[9] Institute of Molecular Biosciences. Biophysics Division. University of Graz. Graz. Austria

[10]Division of Dermatology. Graz. Medical University Graz. Graz. Austria

*authors contributed equally

[#]**Corresponding author:** Asst. Prof. Priv. -Doz. Dr. Beate Rinner

Division of Biomedical Research. Medical University of Graz

Roseggerweg 48. A-8036 Graz. Austria

Tel.: +43 316 385 73524

Fax.: +43 316 385 73009

email: beate.rinner@medunigraz.at

Key words: melanoma. NRAS mutation. exome-sequencing. DNA damage. NFC hydrogel

Supplementary materials

Figure legends

Supplemented Figure S1: MUG-Mel2 showed a variety of CNA frequently occurring in melanoma. publicly available copy number data from Progenetix database.

Supplemental Table S1: STR genotype of the patient tumour tissue and two different passages of MUG-Mel2 (p9 and p60) cell line.

Supplemental Table S2: Focal amplifications of genes in MUG-Mel2 involved in RAS/RAF pathway

Supplemental Table S3: List of the mutated genes by targeted exome sequencing analysis of MUG-Mel2 cell line. For each gene. the number of total mutations. the number of damaging mutations (including missense. frameshift and nonsense). the total length of the gene region targeted by the sequencing. and the mutational burden expressed as the number of total mutation/kb and number of damaging mutations/kb is reported.

Supplemental Table S4: List of the 647 somatic mutations found by targeted exomes sequencing in MUG-Mel2 cell line.

Supplemental Table S5: Frequency of mutations of 50 representative mutations across passages 9 and 60 of MUG-Mel2 cell line.

Figures and Tables

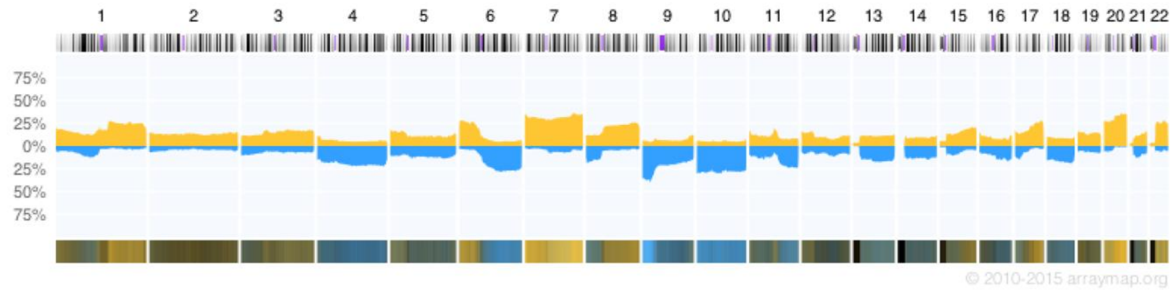

Figure 1

STR-Locus	tissue	MUG-Mel2 (p9)	MUG-Mel2 (p60)
D3S1358	14.15	14.15	14.15
TH01	10	10	9.3
D21S11	29. 31.2	29. 31.2	29. 31.2
D18S51	13.18	12	13
Penta E	7.14	7.14	7.14
D5S818	12.13	12	12
D13S317	12	12	12
D7S820	10.11	10.11	10.11
D16S539	10.11	10.11	10.11
CSF1PO	11	11	11
Penta D	9.13	9.13	9.13
Amelogenin	X.Y	X.Y	X
vWA	15.17	15.17	15.17
D8S1179	13.15	13.15	13.15
TPOX	9.11	9.11	9.11
FGA	22. 23.2	22	22

Table S1

Location	Chromosome	Start	End	Size [Mb]	Log2 Ratio	CN
AKT1 at chr14:105235687-105262080	chr14	102402822	106324231	3.92	-0.42	1.50
BRAF at chr7:140433813-140624564	chr7	102768975	159071691	56.30	0.5352	2.90
EGFR at chr7:55086725-55238738	chr7	6781474	55605006	48.82	0.5558	2.94
KRAS at chr12:25357723-25403865	chr12	8149873	31383502	23.23	0.5396	2.91
MAPK1 at chr22:22123319-22221970	chr15	0	20174282	20.17	0.1012	2.15
MAPK2 at chr22:22127162-22160276	chr22	0	51176570	51.18	0.00	1.99
MAPK3 at chr16:30125426-30134630	chr16	0	5081019	5.08	0.3128	2.48
MAPK4 at chr18:48086484-48258196	chr18	0	76995117	77.00	-0.4090	1.51
MAPK6 at chr15:52311411-52358462	chr15	42037830	52393746	10.36	0.2456	2.37
MAPK7 at chr17:19281774-19286857	chr17	0	20520571	20.52	-0.5072	1.41
MAPK8 at chr10:49514682-49647402	chr10	47675521	50046852	2.37	-0.5136	1.40
MAPK9 at chr5:179660595-179719071	chr5	59327199	180730344	121.40	-0.4132	1.50
MAPK10 at chr4:86933449-87292020	chr4	70256443	190955048	120.70	-0.4275	1.49
MAPK11 at chr22:50702142-50708822	chr15	0	20174282	20.17	0.1012	2.15
MAPK12 at chr22:50691330-50700239	chr15	0	20174282	20.17	0.1012	2.15
MAPK13 at chr6:36098261-36112301	chr6	33104000	37505569	4.40	0.0889	2.13
MAPK14 at chr6:35995454-36079013	chr6	33104000	37505569	4.40	0.0889	2.13
MAPK15 at chr8:144798507-144804633	chr8	141381954	146247376	4.87	0.3799	2.60
MEK at chr15:66727375-66782101	chr15	0	20174282	20.17	0.1012	2.15
NRAS at chr1:115247085-115259515	chr1	11109512	14129531	3.02	-0.4828	1.43
PIK3CA at chr3:178866311-178957881	chr3	162627563	197857588	35.23	0.54	2.91

Table S2

Gene	N of mutation	N of damaging mutations	Total target length	Total mutation burden(mut per 1Kb)	Damaging mutation burden (mut per 1Kb)
KIR2DL1	16	8	1036	15.4	7.7
KIR2DL3	8	5	722	11.1	6.9
AQP7	8	4	679	11.8	5.9
HLA-DRB5	9	4	693	13.0	5.8
SAA2	2	2	381	5.2	5.2
HLA-A	5	4	812	6.2	4.9
KIR3DL1	9	6	1323	6.8	4.5
HLA-DPB1	7	3	769	9.1	3.9
HLA-C	2	1	271	7.4	3.7
CCL22	1	1	276	3.6	3.6
HLA-DRB1	3	2	592	5.1	3.4
MICA	4	4	1190	3.4	3.4
PRB3	1	1	306	3.3	3.3
FRG1	7	5	1593	4.4	3.1
TPSB2	2	2	819	2.4	2.4
HLA-DQB1	1	1	412	2.4	2.4
LHB	1	1	426	2.3	2.3
CDA	1	1	441	2.3	2.3
GYPA	1	1	453	2.2	2.2
NINJ1	1	1	453	2.2	2.2
NDUFA6	1	1	465	2.2	2.2
LOR	3	2	939	3.2	2.1
IL17F	1	1	492	2.0	2.0
MUC7	2	2	1129	1.8	1.8
IFNA10	1	1	566	1.8	1.8
NRAS	1	1	570	1.8	1.8
MUC3A	1	1	608	1.6	1.6
CD177	2	2	1302	1.5	1.5
CIDEA	1	1	652	1.5	1.5
GJC2	2	2	1320	1.5	1.5
CD79B	1	1	693	1.4	1.4
MGMT	1	1	709	1.4	1.4
TM4SF19	1	1	726	1.4	1.4
EMG1	2	1	733	2.7	1.4
TMEM216	1	1	734	1.4	1.4
TPTE	3	2	1540	1.9	1.3
CYP4F2	2	2	1548	1.3	1.3
CA2	1	1	783	1.3	1.3
FCGR3B	2	1	802	2.5	1.2
HYDIN	33	17	14357	2.3	1.2
DLX5	1	1	864	1.2	1.2
OTX2	1	1	894	1.1	1.1
DNASE1L3	1	1	907	1.1	1.1
SMUG1	1	1	933	1.1	1.1
EDA2R	1	1	948	1.1	1.1
CLEC11A	1	1	965	1.0	1.0
CD207	1	1	987	1.0	1.0
RRH	1	1	1014	1.0	1.0
WDR45B	1	1	1035	1.0	1.0
GALE	1	1	1047	1.0	1.0
SETD8	2	1	1048	1.9	1.0

ABHD5	1	1	1050	1.0	1.0
NBPF1	4	3	3159	1.3	0.9
ZPBP	1	1	1056	0.9	0.9
LRPAP1	1	1	1063	0.9	0.9
CCRL2	1	1	1066	0.9	0.9
ZDHHC15	1	1	1077	0.9	0.9
FFAR4	1	1	1086	0.9	0.9
KIR2DL4	3	1	1122	2.7	0.9
APOBEC3B	1	1	1138	0.9	0.9
NR1I3	1	1	1169	0.9	0.9
MTFMT	1	1	1170	0.9	0.9
NCF1	1	1	1173	0.9	0.9
KCNJ9	1	1	1177	0.8	0.8
TNFAIP3	2	2	2362	0.8	0.8
NPSR1	1	1	1209	0.8	0.8
GOT1	1	1	1230	0.8	0.8
SERPINH1	1	1	1257	0.8	0.8
GATM	1	1	1272	0.8	0.8
NCF4	1	1	1309	0.8	0.8
GATA4	1	1	1329	0.8	0.8
KIR3DL2	2	1	1356	1.5	0.7
ADAM19	2	2	2731	0.7	0.7
ODC1	1	1	1373	0.7	0.7
FGG	1	1	1377	0.7	0.7
SERPINC1	1	1	1395	0.7	0.7
MMP12	2	1	1399	1.4	0.7
GTF2IRD2	3	2	2832	1.1	0.7
CYP2C9	1	1	1473	0.7	0.7
GRIA3	3	2	2966	1.0	0.7
SERPINF2	1	1	1486	0.7	0.7
ANXA11	2	1	1501	1.3	0.7
CYP24A1	1	1	1545	0.6	0.6
KCNJ3	1	1	1562	0.6	0.6
SLC1A1	2	1	1575	1.3	0.6
RPE65	1	1	1602	0.6	0.6
MAVS	1	1	1614	0.6	0.6
LIFR	2	2	3294	0.6	0.6
TROVE2	1	1	1647	0.6	0.6
SCARB1	1	1	1650	0.6	0.6
MCCC2	1	1	1692	0.6	0.6
GAD2	1	1	1739	0.6	0.6
CD96	1	1	1758	0.6	0.6
TAF15	1	1	1779	0.6	0.6
UNC93B1	1	1	1793	0.6	0.6
GPI	1	1	1794	0.6	0.6
TOX3	2	1	1795	1.1	0.6
PPM1D	1	1	1809	0.6	0.6
ACSM3	1	1	1835	0.5	0.5
COG8	1	1	1839	0.5	0.5
SIGLEC12	2	1	1850	1.1	0.5
GP6	1	1	1863	0.5	0.5
HSPA1L	1	1	1922	0.5	0.5
KRT1	1	1	1935	0.5	0.5
GP1BA	1	1	1959	0.5	0.5
SLC34A2	2	1	2073	1.0	0.5
SLCO1B3	1	1	2092	0.5	0.5

TPRN	1	1	2136	0.5	0.5
KEL	1	1	2199	0.5	0.5
MPO	1	1	2238	0.4	0.4
SYTL5	1	1	2239	0.4	0.4
RXFP2	1	1	2265	0.4	0.4
CEL	3	1	2271	1.3	0.4
MLH1	1	1	2283	0.4	0.4
ANKK1	1	1	2287	0.4	0.4
APP	1	1	2355	0.4	0.4
COG4	1	1	2370	0.4	0.4
CDH8	1	1	2386	0.4	0.4
PLG	1	1	2437	0.4	0.4
SELP	1	1	2474	0.4	0.4
ATP6V0A1	1	1	2511	0.4	0.4
TAS1R1	1	1	2517	0.4	0.4
ESPN	1	1	2565	0.4	0.4
FMN2	5	2	5148	1.0	0.4
BCL11A	1	1	2599	0.4	0.4
GRM6	2	1	2634	0.8	0.4
PKP2	1	1	2646	0.4	0.4
PRKD3	1	1	2652	0.4	0.4
ACTN2	2	1	2685	0.7	0.4
PRDM9	1	1	2685	0.4	0.4
DPP6	1	1	2706	0.4	0.4
ITIH1	1	1	2711	0.4	0.4
HK2	1	1	2754	0.4	0.4
ZC3H3	1	1	2832	0.4	0.4
PMS2	1	1	2836	0.4	0.4
MIA3	2	2	5693	0.4	0.4
WDR36	1	1	2856	0.4	0.4
GRIK1	1	1	2889	0.3	0.3
TRPC5	1	1	2909	0.3	0.3
DDX11	2	1	2918	0.7	0.3
SRGAP2	3	1	2933	1.0	0.3
NEFH	1	1	3063	0.3	0.3
NLRP3	1	1	3111	0.3	0.3
AP3B1	1	1	3285	0.3	0.3
SPINK5	1	1	3297	0.3	0.3
GRIN3A	1	1	3348	0.3	0.3
JAK2	1	1	3373	0.3	0.3
COL6A2	1	1	3382	0.3	0.3
ZAN	2	2	6936	0.3	0.3
WDR35	1	1	3546	0.3	0.3
TYK2	1	1	3564	0.3	0.3
PER3	1	1	3582	0.3	0.3
TAF2	1	1	3600	0.3	0.3
CTC1	1	1	3628	0.3	0.3
ZNF804A	2	1	3630	0.6	0.3
PML	1	1	3658	0.3	0.3
ADAMTS18	1	1	3666	0.3	0.3
PEX19	1	1	3693	0.3	0.3
TRIOBP	2	2	7392	0.3	0.3
ADAMTS2	1	1	3708	0.3	0.3
PHKA2	1	1	3708	0.3	0.3
JAG2	1	1	3717	0.3	0.3
ATP2B2	1	1	3732	0.3	0.3

TRAPPC9	1	1	3741	0.3	0.3
MAGEL2	2	1	3746	0.5	0.3
IRS4	1	1	3774	0.3	0.3
MYO6	1	1	3858	0.3	0.3
RPGRIP1	1	1	3861	0.3	0.3
SH3TC2	1	1	3867	0.3	0.3
PFAS	1	1	3987	0.3	0.3
MSH6	1	1	4083	0.2	0.2
MTUS1	1	1	4105	0.2	0.2
TG	2	2	8307	0.2	0.2
MST1R	1	1	4180	0.2	0.2
NPHP4	1	1	4281	0.2	0.2
PIK3C2G	1	1	4304	0.2	0.2
ARHGAP31	1	1	4335	0.2	0.2
PTPRT	1	1	4348	0.2	0.2
ATP10A	1	1	4476	0.2	0.2
TRPM2	2	1	4477	0.4	0.2
TOPBP1	1	1	4539	0.2	0.2
GOLGA3	1	1	4606	0.2	0.2
ABCC3	1	1	4631	0.2	0.2
KMT2C	5	3	13956	0.4	0.2
IQSEC2	1	1	4689	0.2	0.2
EIF4G1	1	1	4824	0.2	0.2
IQGAP3	1	1	4855	0.2	0.2
CLTCL1	2	1	4888	0.4	0.2
IQGAP1	2	1	4933	0.4	0.2
PCNT	2	2	10011	0.2	0.2
CAMTA1	1	1	5069	0.2	0.2
LTBP4	1	1	5159	0.2	0.2
TSC2	1	1	5424	0.2	0.2
SI	2	1	5484	0.4	0.2
LAMA5	3	2	11005	0.3	0.2
GRIN2A	2	1	5646	0.4	0.2
CUL7	1	1	5929	0.2	0.2
SCN9A	1	1	5934	0.2	0.2
CCDC88C	1	1	6087	0.2	0.2
ANK1	1	1	6114	0.2	0.2
MYO7B	1	1	6302	0.2	0.2
DYSF	1	1	6490	0.2	0.2
DNAH5	2	2	13875	0.1	0.1
PRPF8	1	1	7008	0.1	0.1
CACNA1H	1	1	7062	0.1	0.1
GPR179	1	1	7090	0.1	0.1
CACNA1C	1	1	7106	0.1	0.1
PLCE1	1	1	7191	0.1	0.1
HERC2	4	2	14410	0.3	0.1
CREBBP	2	1	7329	0.3	0.1
PTPN13	2	1	7423	0.3	0.1
NOTCH2	2	1	7469	0.3	0.1
PCDH15	1	1	7504	0.1	0.1
COL6A5	1	1	7539	0.1	0.1
ATR	1	1	7935	0.1	0.1
CHD6	2	1	8109	0.2	0.1
NIPBL	1	1	8460	0.1	0.1
AKAP13	1	1	8548	0.1	0.1
COL7A1	1	1	8835	0.1	0.1

ATM	1	1	9171	0.1	0.1
COL6A3	1	1	9581	0.1	0.1
VPS13A	1	1	9645	0.1	0.1
RANBP2	1	1	9675	0.1	0.1
ZFHX4	1	1	10838	0.1	0.1
PRKDC	1	1	12386	0.1	0.1
PKD1	5	1	12912	0.4	0.1
APOB	1	1	13692	0.1	0.1
NEB	2	1	25683	0.1	0.0
TTN	4	4	110459	0.0	0.0
COX7A1	1	0	233	4.3	0.0
GPX1	2	0	649	3.1	0.0
MGST2	1	0	444	2.3	0.0
SPRR3	1	0	506	2.0	0.0
ARF4	1	0	534	1.9	0.0
APRT	1	0	548	1.8	0.0
VCX3A	1	0	556	1.8	0.0
ALOX5AP	1	0	648	1.5	0.0
CRP	1	0	670	1.5	0.0
PMS2P3	2	0	1388	1.4	0.0
RSPO4	1	0	705	1.4	0.0
NCR3	1	0	706	1.4	0.0
PRSS1	1	0	744	1.3	0.0
B3GALTL	2	0	1497	1.3	0.0
PGAM2	1	0	762	1.3	0.0
CIDEC	1	0	786	1.3	0.0
PNPLA1	2	0	1615	1.2	0.0
COMT	1	0	816	1.2	0.0
TTPA	1	0	837	1.2	0.0
GGT5	2	0	1749	1.1	0.0
CHDH	2	0	1775	1.1	0.0
SYNGR1	1	0	897	1.1	0.0
FCAR	1	0	924	1.1	0.0
FCGR2A	1	0	944	1.1	0.0
ANKRD1	1	0	960	1.0	0.0
PSMD7	1	0	965	1.0	0.0
TAL1	1	0	990	1.0	0.0
NEIL2	1	0	1004	1.0	0.0
PAWR	1	0	1014	1.0	0.0
TPM2	1	0	1014	1.0	0.0
CDK7	1	0	1026	1.0	0.0
GBGT1	1	0	1035	1.0	0.0
PRPH2	1	0	1041	1.0	0.0
SUCLG1	1	0	1041	1.0	0.0
PTGER2	1	0	1072	0.9	0.0
SERPINB5	1	0	1119	0.9	0.0
NEU2	1	0	1138	0.9	0.0
AIPL1	1	0	1155	0.9	0.0
RASSF1	1	0	1172	0.9	0.0
BCKDHB	1	0	1179	0.8	0.0
MAF	1	0	1216	0.8	0.0
ADORA2A	1	0	1234	0.8	0.0
SERPINI2	1	0	1236	0.8	0.0
IDH1	1	0	1245	0.8	0.0
PSTPIP1	1	0	1251	0.8	0.0
CPB2	1	0	1258	0.8	0.0

STAT6	2	0	2520	0.8	0.0
GFI1	1	0	1269	0.8	0.0
PROZ	1	0	1269	0.8	0.0
C2orf71	3	0	3867	0.8	0.0
GAL3ST3	1	0	1291	0.8	0.0
STK11	1	0	1302	0.8	0.0
PWRN1	1	0	1304	0.8	0.0
TSPYL1	1	0	1314	0.8	0.0
CADM1	1	0	1329	0.8	0.0
ADRA2B	1	0	1344	0.7	0.0
IFI44L	1	0	1348	0.7	0.0
AP4M1	1	0	1362	0.7	0.0
CHRM2	1	0	1401	0.7	0.0
NEIL1	1	0	1418	0.7	0.0
KYNU	1	0	1420	0.7	0.0
HADHB	1	0	1425	0.7	0.0
ADAMTSL2	2	0	2856	0.7	0.0
MLYCD	1	0	1482	0.7	0.0
CYP21A2	2	0	2968	0.7	0.0
FKRP	1	0	1488	0.7	0.0
SLC19A3	1	0	1491	0.7	0.0
CYP2C8	1	0	1498	0.7	0.0
MUC13	1	0	1522	0.7	0.0
RPS6KB1	1	0	1572	0.6	0.0
TXNRD2	1	0	1575	0.6	0.0
UGT2B7	1	0	1581	0.6	0.0
CHRNA3	1	0	1586	0.6	0.0
ICAM1	1	0	1589	0.6	0.0
NEFL	1	0	1632	0.6	0.0
ILDR1	1	0	1641	0.6	0.0
PDPK1	1	0	1654	0.6	0.0
FOXC1	1	0	1662	0.6	0.0
CES1	1	0	1707	0.6	0.0
AMHR2	1	0	1722	0.6	0.0
YTHDF2	1	0	1732	0.6	0.0
MYT1L	2	0	3532	0.6	0.0
MITF	1	0	1786	0.6	0.0
GALNT9	1	0	1798	0.6	0.0
GSE1	2	0	3654	0.5	0.0
PAPSS2	1	0	1860	0.5	0.0
SLC6A18	1	0	1872	0.5	0.0
BCR	2	0	3790	0.5	0.0
SMPD1	1	0	1896	0.5	0.0
SLC6A8	1	0	1908	0.5	0.0
RPN2	1	0	1944	0.5	0.0
HDC	1	0	1989	0.5	0.0
EOMES	1	0	2052	0.5	0.0
BMPER	1	0	2058	0.5	0.0
SLC27A5	1	0	2060	0.5	0.0
DCLRE1C	1	0	2079	0.5	0.0
HRC	1	0	2091	0.5	0.0
TGM6	1	0	2121	0.5	0.0
FMO6P	1	0	2125	0.5	0.0
EEF2K	1	0	2158	0.5	0.0
CEP41	3	0	6554	0.5	0.0
NDUFS1	1	0	2226	0.4	0.0

MYB	1	0	2267	0.4	0.0
HADHA	1	0	2292	0.4	0.0
SLC26A6	1	0	2292	0.4	0.0
SEC23A	1	0	2298	0.4	0.0
LOXL2	1	0	2309	0.4	0.0
LMF1	2	0	4674	0.4	0.0
WT1-AS	1	0	2338	0.4	0.0
CDH5	1	0	2341	0.4	0.0
AMT	1	0	2405	0.4	0.0
TMC6	1	0	2418	0.4	0.0
SEMA4C	1	0	2485	0.4	0.0
SETBP1	2	0	4980	0.4	0.0
HIF1A	1	0	2569	0.4	0.0
TLR5	1	0	2577	0.4	0.0
CCHCR1	1	0	2595	0.4	0.0
LITAF	1	0	2617	0.4	0.0
GRM3	1	0	2632	0.4	0.0
FGFR1	1	0	2635	0.4	0.0
UBE3A	1	0	2648	0.4	0.0
LAMB1	2	0	5325	0.4	0.0
LPIN1	1	0	2673	0.4	0.0
CBL	1	0	2721	0.4	0.0
RALGDS	1	0	2763	0.4	0.0
MYO5B	2	0	5547	0.4	0.0
MSH4	1	0	2788	0.4	0.0
MAN1B1	1	0	2900	0.3	0.0
AARS	1	0	2907	0.3	0.0
PDE11A	1	0	2964	0.3	0.0
ANO7	1	0	2982	0.3	0.0
ALG2	1	0	2989	0.3	0.0
ACAN	1	0	3027	0.3	0.0
LIG3	1	0	3058	0.3	0.0
TMPRSS15	1	0	3060	0.3	0.0
GLDC	1	0	3063	0.3	0.0
ANO6	1	0	3076	0.3	0.0
NRP2	1	0	3104	0.3	0.0
NLRP7	1	0	3114	0.3	0.0
CLU	1	0	3146	0.3	0.0
LLGL1	1	0	3170	0.3	0.0
SP100	1	0	3192	0.3	0.0
INVS	1	0	3198	0.3	0.0
CFL2	1	0	3270	0.3	0.0
USP7	1	0	3275	0.3	0.0
DISC1	1	0	3277	0.3	0.0
ADNP	1	0	3303	0.3	0.0
WWC1	1	0	3334	0.3	0.0
ZEB1	1	0	3385	0.3	0.0
CACNA2D4	1	0	3414	0.3	0.0
TSC1	1	0	3495	0.3	0.0
LAMB3	1	0	3519	0.3	0.0
SREBF1	1	0	3534	0.3	0.0
PLEKHG4	1	0	3576	0.3	0.0
PLCB4	1	0	3581	0.3	0.0
TRERF1	1	0	3586	0.3	0.0
PARD3B	1	0	3592	0.3	0.0
DIAPH3	1	0	3602	0.3	0.0

ACACB	2	0	7322	0.3	0.0
WDR11	1	0	3675	0.3	0.0
COG6	1	0	3696	0.3	0.0
ERBB2	1	0	3738	0.3	0.0
CNGB1	1	0	3782	0.3	0.0
MYBPC3	1	0	3825	0.3	0.0
NPC1	1	0	3837	0.3	0.0
CNTNAP5	1	0	3921	0.3	0.0
PTPRC	1	0	3926	0.3	0.0
PHLPP2	1	0	3951	0.3	0.0
GIGYF2	1	0	3966	0.3	0.0
CNTNAP2	1	0	3996	0.3	0.0
KIF7	1	0	4032	0.2	0.0
FAM20A	1	0	4110	0.2	0.0
ACSF3	1	0	4209	0.2	0.0
XIAP	2	0	8419	0.2	0.0
MMAB	1	0	4225	0.2	0.0
DAPK1	1	0	4265	0.2	0.0
WNK1	2	0	8643	0.2	0.0
GRIN2B	1	0	4455	0.2	0.0
COL5A2	1	0	4500	0.2	0.0
MAP3K1	1	0	4539	0.2	0.0
GNAS	1	0	4586	0.2	0.0
TMCO1	1	0	4650	0.2	0.0
ALS2	1	0	5052	0.2	0.0
COL4A4	1	0	5073	0.2	0.0
UQCRB	1	0	5109	0.2	0.0
UBR1	1	0	5250	0.2	0.0
SYT14	1	0	5388	0.2	0.0
DLG5	1	0	5725	0.2	0.0
ANAPC1	1	0	5785	0.2	0.0
MYH15	1	0	5796	0.2	0.0
MYH7	1	0	5808	0.2	0.0
DOCK3	1	0	6037	0.2	0.0
MYPN	1	0	6165	0.2	0.0
KAT6B	1	0	6222	0.2	0.0
OTOF	1	0	6319	0.2	0.0
DOCK9	1	0	6335	0.2	0.0
MUC5B	1	0	6345	0.2	0.0
PTPRQ	1	0	6384	0.2	0.0
F5	1	0	6675	0.1	0.0
ABCA4	1	0	6822	0.1	0.0
EFHC1	1	0	6845	0.1	0.0
FOXP2	1	0	6978	0.1	0.0
CR1	1	0	7420	0.1	0.0
CEP290	1	0	7440	0.1	0.0
MYO18B	1	0	7659	0.1	0.0
VAPB	1	0	7938	0.1	0.0
COL6A4P2	1	0	9157	0.1	0.0
HTT	1	0	9359	0.1	0.0
CSMD1	1	0	10622	0.1	0.0
SPTBN5	1	0	10850	0.1	0.0
SMG1	1	0	10920	0.1	0.0
ZFHX3	1	0	11100	0.1	0.0
VPS13B	1	0	12255	0.1	0.0
ALMS1	1	0	12504	0.1	0.0

DNAH9	1	0	13389	0.1	0.0
PLEC	1	0	14728	0.1	0.0
RNF213	1	0	15792	0.1	0.0
OBSCN	1	0	25109	0.0	0.0
SYNE1	1	0	26488	0.0	0.0
**total**	**647**	**309**	**1673799**	**0.4**	**0.2**

Table S3

variant (gene-chrom-pos-nucleotide)	Gene	Consequence	Protein. Position	Amino. Acids	HGVSc_nomenclature	Transcript	HGVS_mutation	Protein_seq	HGVSp_nomenclature
NPHP4-1-5934950-C>C/T	NPHP4	missense_variant	1010	D/N	NM_015102.3:c.3028G>A	NM_015102.3	c.3028G>A	NP_055917.1	NP_055917.1:p.Asp1010Asn
ESPN-1-6511705-C>C/T	ESPN	missense_variant	653	S/L	NM_031475.2:c.1958C>T	NM_031475.2	c.1958C>T	NP_113663.2	NP_113663.2:p.Ser653Leu
TAS1R1-1-6638925-C>C/T	TAS1R1	missense_variant	603	R/C	NM_138697.3:c.1807C>T	NM_138697.3	c.1807C>T	NP_619642.2	NP_619642.2:p.Arg603Cys
CAMTA1-1-7724050-G>G/A	CAMTA1	missense_variant	481	M/I	NM_015215.2:c.1443G>A	NM_015215.2	c.1443G>A	NP_056030.1	NP_056030.1:p.Met481Ile
PER3-1-7890053-G>G/A	PER3	missense_variant	1007	A/T	NM_016831.1:c.3019G>A	NM_016831.1	c.3019G>A	NP_058515.1	NP_058515.1:p.Ala1007Thr
NBPF1-1-16893721-A>A/C	NBPF1	missense_variant	931	F/C	NM_017940.3:c.2792T>G	NM_017940.3	c.2792T>G	NP_060410.2	NP_060410.2:p.Phe931Cys
NBPF1-1-16895634-T>T/G	NBPF1	missense_variant	850	K/Q	NM_017940.3:c.2548A>C	NM_017940.3	c.2548A>C	NP_060410.2	NP_060410.2:p.Lys850Gln
NBPF1-1-16902894-A>A/G	NBPF1	missense_variant	663	C/R	NM_017940.3:c.1987T>C	NM_017940.3	c.1987T>C	NP_060410.2	NP_060410.2:p.Cys663Arg
NBPF1-1-16907328-G>G/T	NBPF1	synonymous_variant	501	T	NM_017940.3:c.1503C>A	NM_017940.3	c.1503C>A	NP_060410.2	NM_017940.3:c.1503C>A(p.=)
CDA-1-20940341-G>G/A	CDA	missense_variant	91	M/I	NM_001785.2:c.273G>A	NM_001785.2	c.273G>A	NP_001776.1	NP_001776.1:p.Met91Ile
GALE-1-24122489-G>G/T	GALE	missense_variant	333	L/I	NM_001127621.1:c.997C>A	NM_001127621.1	c.997C>A	NP_001121093.1	NP_001121093.1:p.Leu333Ile
YTHDF2-1-29069334-T>T/C	YTHDF2	synonymous_variant	184	T	NM_001173128.1:c.552T>C	NM_001173128.1	c.552T>C	NP_001166599.1	NM_001173128.1:c.552T>C(p.=)
TAL1-1-47691568-GCAGA>GCAGA/G	TAL1	intron_variant	0	NA	NM_003189.2:c.-1-11_-1-8delTCTG	NM_003189.2	c.-1-11_-1-8delTCTG	NP_003180.1	NA
RPE65-1-68904721-T>T/C	RPE65	missense_variant	301	N/S	NM_000329.2:c.902A>G	NM_000329.2	c.902A>G	NP_000320.1	NP_000320.1:p.Asn301Ser
MSH4-1-76346920-C>CAT/CAT	MSH4	intron_variant	0	NA	NM_002440.3:c.1782-11_1782-10insAT	NM_002440.3	c.1782-11_1782-10insAT	NP_002431.2	NA
IFI44L-1-79107305-C>C/T	IFI44L	intron_variant	0	NA	NM_006820.2:c.1324+11C>T	NM_006820.2	c.1324+11C>T	NP_006811.2	NA
GFI1-1-92946037-G>G/A	GFI1	synonymous_variant	264	F	NM_001127216.1:c.792C>T	NM_001127216.1	c.792C>T	NP_001120688.1	NM_001127216.1:c.792C>T(p.=)
ABCA4-1-94544135-A>AC/AC	ABCA4	intron_variant	0	NA	NM_000350.2:c.1356+10dupG	NM_000350.2	c.1356+10dupG	NP_000341.2	NA
NRAS-1-115256529-T>T/C	NRAS	missense_variant	61	Q/R	NM_002524.4:c.182A>G	NM_002524.4	c.182A>G	NP_002515.1	NP_002515.1:p.Gln61Arg
NOTCH2-1-120506250-C>C/T	NOTCH2	missense_variant	621	R/H	NM_024408.3:c.1862G>A	NM_024408.3	c.1862G>A	NP_077719.2	NP_077719.2:p.Arg621His
NOTCH2-1-120539936-C>C/T	NOTCH2	synonymous_variant	145	T	NM_024408.3:c.435G>A	NM_024408.3	c.435G>A	NP_077719.2	NM_024408.3:c.435G>A(p.=)
SPRR3-1-152975763-C>C/T	SPRR3	synonymous_variant	89	G	NM_005416.2:c.267C>T	NM_005416.2	c.267C>T	NP_005407.1	NM_005416.2:c.267C>T(p.=)
LOR-1-153233990-T>T/G	LOR	missense_variant	189	Y/D	NM_000427.2:c.565T>G	NM_000427.2	c.565T>G	NP_000418.2	NP_000418.2:p.Tyr189Asp
LOR-1-153233991-A>A/G	LOR	missense_variant	189	Y/C	NM_000427.2:c.566A>G	NM_000427.2	c.566A>G	NP_000418.2	NP_000418.2:p.Tyr189Cys
LOR-1-153233991-A>ACTCTGGCGGCGG/ACTCTGGCGGCGG	LOR	inframe	189	Y/YSGGG	NM_000427.2:c.566_567insCTCTGGCGGCGG	NM_000427.2	c.566_567insCTCTGGCGGCGG	NP_000418.2	NP_000418.2:p.Tyr189_Ser190insSerGlyGlyGly
IQGAP3-1-156504449-A>A/T	IQGAP3	missense_variant	1195	M/K	NM_178229.4:c.3584T>A	NM_178229.4	c.3584T>A	NP_839943.2	NP_839943.2:p.Met1195Lys
CRP-1-159683483-G>G/A	CRP	synonymous_variant	169	S	NM_000567.2:c.507C>T	NM_000567.2	c.507C>T	NP_000558.2	NM_000567.2:c.507C>T(p.=)
KCNJ9-1-160057516-A>A/G	KCNJ9	missense_variant	364	E/G	NM_004983.2:c.1091A>G	NM_004983.2	c.1091A>G	NP_004974.2	NP_004974.2:p.Glu364Gly
PEX19-1-160254899-C>C/T	PEX19	missense_variant	6	E/K	NM_002857.3:c.16G>A	NM_002857.3	c.16G>A	NP_002848.1	NP_002848.1:p.Glu6Lys
NR1I3-1-161200970-G>G/A	NR1I3	stop_gained	254	R/*	NM_001077482.2:c.760C>T	NM_001077482.2	c.760C>T	NP_001070950.1	NP_001070950.1:p.Arg254Ter
FCGR2A-1-161483723-G>G/A	FCGR2A	splice_variant	0	NA	NM_001136219.1:c.780+1G>A	NM_001136219.1	c.780+1G>A	NP_001129691.1	NA
FCGR3B-1-161599693-T>C/C	FCGR3B	missense_variant	101	N/S	NM_001244753.1:c.302A>G	NM_001244753.1	c.302A>G	NP_001231682.1	NP_001231682.1:p.Asn101Ser
FCGR3B-1-161600181-G>G/A	FCGR3B	intron_variant	0	NA	NM_001244753.1:c.149-3C>T	NM_001244753.1	c.149-3C>T	NP_001231682.1	NA
TMCO1-1-165694251-CTCTCTCTCTCTATA>C/C	TMCO1	3_prime_UTR_variant	0	NA	NM_019026.4:c.*2995_*3008delTATAGAGAGAGAGA	NM_019026.4	c.*2995_*3008delTATAGAGAGAGAGA	NP_061899.2	NA
F5-1-169510577-A>A/G	F5	synonymous_variant	1251	L	NM_000130.4:c.3751T>C	NM_000130.4	c.3751T>C	NP_000121.2	NM_000130.4:c.3751T>C(p.=)
SELP-1-169559424-C>C/T	SELP	missense_variant	819	G/R	NM_003005.3:c.2455G>A	NM_003005.3	c.2455G>A	NP_002996.2	NP_002996.2:p.Gly819Arg
FMO6P-1-171130598-CACACA>CACACA/C	FMO6P	nc_exon_variant	0	NA	NR_002601.1:n.2022_2026delACACA	NR_002601.1	n.2022_2026delACACA	NA	NA
SERPINC1-1-173873176-C>C/A	SERPINC1	missense_variant	416	A/S	NM_000488.3:c.1246G>T	NM_000488.3	c.1246G>T	NP_000479.1	NP_000479.1:p.Ala416Ser
TROVE2-1-193045734-C>C/A	TROVE2	missense_variant	302	S/Y	NM_001173524.1:c.905C>A	NM_001173524.1	c.905C>A	NP_001166995.1	NP_001166995.1:p.Ser302Tyr

PTPRC-1-198685878-G>G/A	PTPRC	synonymous_variant	453	T	NM_002838.4:c.1359G>A	NM_002838.4	c.1359G>A	NP_002829.3	NM_002838.4:c.1359G>A(p.=)
SRGAP2-1-206516261-C>C/T	SRGAP2	synonymous_variant	22	S	NM_015326.2:c.65C>T	NM_015326.2	c.65C>T	NP_056141.2	NM_015326.2:c.65C>T(p.=)
SRGAP2-1-206566904-T>T/C	SRGAP2	missense_variant	150	V/A	NM_015326.2:c.448T>C	NM_015326.2	c.448T>C	NP_056141.2	NP_056141.2:p.Val150Ala
SRGAP2-1-206579936-A>A/G	SRGAP2	intron_variant	0	NA	NM_015326.2:c.1099+3A>G	NM_015326.2	c.1099+3A>G	NP_056141.2	NA
CR1-1-207751193-C>C/T	CR1	synonymous_variant	1527	F	NM_000651.4:c.4581C>T	NM_000651.4	c.4581C>T	NP_000642.3	NM_000651.4:c.4581C>T(p.=)
LAMB3-1-209811256-G>G/A	LAMB3	synonymous_variant	123	F	NM_001127641.1:c.369C>T	NM_001127641.1	c.369C>T	NP_001121113.1	NM_001127641.1:c.369C>T(p.=)
SYT14-1-210337096-CAG>CAG/C	SYT14	3_prime_UTR_variant	0	NA	NM_001146261.2:c.*2710_*2711delAG	NM_001146261.2	c.*2710_*2711delAG	NP_001139733.1	NA
MIA3-1-222803062-A>A/G	MIA3	missense_variant	834	I/V	NM_198551.2:c.2500A>G	NM_198551.2	c.2500A>G	NP_940953.2	NP_940953.2:p.Ile834Val
MIA3-1-222803422-G>G/A	MIA3	missense_variant	954	E/K	NM_198551.2:c.2860G>A	NM_198551.2	c.2860G>A	NP_940953.2	NP_940953.2:p.Glu954Lys
TLR5-1-223283881-T>T/C	TLR5	synonymous_variant	831	K	NM_003268.5:c.2493A>G	NM_003268.5	c.2493A>G	NP_003259.2	NM_003268.5:c.2493A>G(p.=)
GJC2-1-228346057-G>G/A	GJC2	missense_variant	200	G/R	NM_020435.3:c.598G>A	NM_020435.3	c.598G>A	NP_065168.2	NP_065168.2:p.Gly200Arg
GJC2-1-228346058-G>G/A	GJC2	missense_variant	200	G/E	NM_020435.3:c.599G>A	NM_020435.3	c.599G>A	NP_065168.2	NP_065168.2:p.Gly200Glu
OBSCN-1-228560326-C>C/A	OBSCN	synonymous_variant	7283	R	NM_001098623.1:c.21847C>A	NM_001098623.1	c.21847C>A	NP_001092093.1	NM_001098623.1:c.21847C>A(p.=)
DISC1-1-231990724-A>A/G	DISC1	intron_variant	0	NA	NM_001164537.1:c.2077+36461A>G	NM_001164537.1	c.2077+36461A>G	NP_001158009.1	NA
ACTN2-1-236898949-C>C/T	ACTN2	missense_variant	238	P/S	NM_001103.2:c.712C>T	NM_001103.2	c.712C>T	NP_001094.1	NP_001094.1:p.Pro238Ser
ACTN2-1-236924311-C>C/T	ACTN2	intron_variant	0	NA	NM_001103.2:c.2368-4C>T	NM_001103.2	c.2368-4C>T	NP_001094.1	NA
FMN2-1-240370899-A>A/G	FMN2	synonymous_variant	929	A	NM_020066.4:c.2787A>G	NM_020066.4	c.2787A>G	NP_064450.3	NM_020066.4:c.2787A>G(p.=)
FMN2-1-240370907-T>T/C	FMN2	missense_variant	932	L/P	NM_020066.4:c.2795T>C	NM_020066.4	c.2795T>C	NP_064450.3	NP_064450.3:p.Leu932Pro
FMN2-1-240370908-C>C/T	FMN2	synonymous_variant	932	L	NM_020066.4:c.2796C>T	NM_020066.4	c.2796C>T	NP_064450.3	NM_020066.4:c.2796C>T(p.=)
FMN2-1-240370913-T>T/C	FMN2	missense_variant	934	L/P	NM_020066.4:c.2801T>C	NM_020066.4	c.2801T>C	NP_064450.3	NP_064450.3:p.Leu934Pro
FMN2-1-240371145-T>T/C	FMN2	synonymous_variant	1011	P	NM_020066.4:c.3033T>C	NM_020066.4	c.3033T>C	NP_064450.3	NM_020066.4:c.3033T>C(p.=)
NLRP3-1-247608066-G>G/A	NLRP3	missense_variant	985	G/E	NM_001079821.2:c.2954G>A	NM_001079821.2	c.2954G>A	NP_001073289.1	NP_001073289.1:p.Gly985Glu
MYT1L-2-1842968-T>T/A	MYT1L	synonymous_variant	1009	G	NM_015025.2:c.3027A>T	NM_015025.2	c.3027A>T	NP_055840.2	NM_015025.2:c.3027A>T(p.=)
MYT1L-2-1946770-TTCCTCC>TTCCTCC/T	MYT1L	inframe	NA	EEE/E	NM_015025.2:c.483_488delGGAGGA	NM_015025.2	c.483_488delGGAGGA	NP_055840.2	NP_055840.2:p.Glu162_Glu163del
ODC1-2-10583672-C>C/T	ODC1	missense_variant	204	D/N	NM_002539.1:c.610G>A	NM_002539.1	c.610G>A	NP_002530.1	NP_002530.1:p.Asp204Asn
LPIN1-2-11919638-C>C/T	LPIN1	intron_variant	0	NA	NM_001261428.1:c.978-7C>T	NM_001261428.1	c.978-7C>T	NP_001248357.1	NA
WDR35-2-20153619-C>C/T	WDR35	missense_variant	470	R/Q	NM_001006657.1:c.1409G>A	NM_001006657.1	c.1409G>A	NP_001006658.1	NP_001006658.1:p.Arg470Gln
APOB-2-21260123-G>G/A	APOB	missense_variant	181	T/I	NM_000384.2:c.542C>T	NM_000384.2	c.542C>T	NP_000375.2	NP_000375.2:p.Thr181Ile
HADHA-2-26415280-C>C/T	HADHA	synonymous_variant	633	G	NM_000182.4:c.1899G>A	NM_000182.4	c.1899G>A	NP_000173.2	NM_000182.4:c.1899G>A(p.=)
HADHB-2-26477125-G>GACT/GACT	HADHB	inframe	NA	-/T	NM_000183.2:c.3_4insACT	NM_000183.2	c.3_4insACT	NP_000174.1	NP_000174.1:p.Met1_Thr2insThr
OTOF-2-26689571-G>G/A	OTOF	intron_variant	0	NA	NM_194248.2:c.4500+11C>T	NM_194248.2	c.4500+11C>T	NP_919224.1	NA
C2orf71-2-29287926-C>C/CGCT	C2orf71	inframe	NA	-/S	NM_001029883.2:c.3673_3675dupAGC	NM_001029883.2	c.3673_3675dupAGC	NP_001025054.1	NP_001025054.1:p.Ser1225dup
C2orf71-2-29293951-A>A/G	C2orf71	synonymous_variant	1059	P	NM_001029883.2:c.3177T>C	NM_001029883.2	c.3177T>C	NP_001025054.1	NM_001029883.2:c.3177T>C(p.=)
C2orf71-2-29296447-C>C/T	C2orf71	synonymous_variant	227	E	NM_001029883.2:c.681G>A	NM_001029883.2	c.681G>A	NP_001025054.1	NM_001029883.2:c.681G>A(p.=)
PRKD3-2-37520362-T>T/C	PRKD3	missense_variant	114	H/R	NM_005813.3:c.341A>G	NM_005813.3	c.341A>G	NP_005804.1	NP_005804.1:p.His114Arg

MSH6-2-48023195-A>A/C	MSH6	missense_variant	207	E/A	NM_000179.2:c.620A>C	NM_000179.2	c.620A>C	NP_000170.1	NP_000170.1:p.Glu207Ala
BCL11A-2-60688231-C>C/T	BCL11A	missense_variant	606	G/S	NM_022893.3:c.1816G>A	NM_022893.3	c.1816G>A	NP_075044.2	NP_075044.2:p.Gly606Ser
PUS10.PEX13-2-61244926-C>C/T	PUS10.PEX13	intron_variant	0	NA	NM_144709.2:c.-16+217G>A	NM_144709.2	c.-16+217G>A	NP_653310.2	NA
CD207-2-71058929-C>C/T	CD207	missense_variant	247	G/R	NM_015717.3:c.739G>A	NM_015717.3	c.739G>A	NP_056532.3	NP_056532.3:p.Gly247Arg
DYSF-2-71908201-G>G/A	DYSF	missense_variant	2045	R/Q	NM_001130987.1:c.6134G>A	NM_001130987.1	c.6134G>A	NP_001124459.1	NP_001124459.1:p.Arg2045Gln
ALMS1-2-73675227-T>TCTC/TCTC	ALMS1	inframe	524	S/SP	NM_015120.4:c.1570_1571insCTC	NM_015120.4	c.1570_1571insCTC	NP_055935.4	NP_055935.4:p.Ser524_Leu525insPro
HK2-2-75106027-C>C/T	HK2	missense_variant	415	S/F	NM_000189.4:c.1244C>T	NM_000189.4	c.1244C>T	NP_000180.2	NP_000180.2:p.Ser415Phe
SUCLG1-2-84668557-A>A/G	SUCLG1	synonymous_variant	115	A	NM_003849.3:c.345T>C	NM_003849.3	c.345T>C	NP_003840.2	NM_003849.3:c.345T>C(p.=)
ADRA2B-2-96780986-C>CTCCTCCTCT/CTCCTCCTCT	ADRA2B	inframe	301	E/EEEE	NM_000682.5:c.894_902dupAGAGGAGGA	NM_000682.5	c.894_902dupAGAGGAGGA	NP_000673.2	NP_000673.2:p.Glu298_Glu300dup
SEMA4C-2-97527545-G>G/A	SEMA4C	synonymous_variant	510	V	NM_017789.4:c.1530C>T	NM_017789.4	c.1530C>T	NP_060259.4	NM_017789.4:c.1530C>T(p.=)
RANBP2-2-109383674-T>T/C	RANBP2	missense_variant	2227	Y/H	NM_006267.4:c.6679T>C	NM_006267.4	c.6679T>C	NP_006258.3	NP_006258.3:p.Tyr2227His
ANAPC1-2-112536264-G>T/T	ANAPC1	synonymous_variant	1791	I	NM_022662.3:c.5373C>A	NM_022662.3	c.5373C>A	NP_073153.1	NM_022662.3:c.5373C>A(p.=)
CNTNAP5-2-125367427-C>C/T	CNTNAP5	synonymous_variant	601	A	NM_130773.2:c.1803C>T	NM_130773.2	c.1803C>T	NP_570129.1	NM_130773.2:c.1803C>T(p.=)
MYO7B-2-128351149-T>T/G	MYO7B	missense_variant	725	L/R	NM_001080527.1:c.2174T>G	NM_001080527.1	c.2174T>G	NP_001073996.1	NP_001073996.1:p.Leu725Arg
KYNU-2-143742682-T>T/C	KYNU	synonymous_variant	253	H	NM_003937.2:c.759T>C	NM_003937.2	c.759T>C	NP_003928.1	NM_003937.2:c.759T>C(p.=)
NEB-2-152448640-T>T/C	NEB	missense_variant	4912	N/D	NM_001164507.1:c.14734A>G	NM_001164507.1	c.14734A>G	NP_001157979.1	NP_001157979.1:p.Asn4912Asp
NEB-2-152501045-G>G/A	NEB	synonymous_variant	2527	Y	NM_001164507.1:c.7581C>T	NM_001164507.1	c.7581C>T	NP_001157979.1	NM_001164507.1:c.7581C>T(p.=)
KCNJ3-2-155555406-A>A/G	KCNJ3	missense_variant	40	K/R	NM_002239.3:c.119A>G	NM_002239.3	c.119A>G	NP_002230.1	NP_002230.1:p.Lys40Arg
SCN9A-2-167060594-A>A/G	SCN9A	missense_variant	1538	W/R	NM_002977.3:c.4612T>C	NM_002977.3	c.4612T>C	NP_002968.1	NP_002968.1:p.Trp1538Arg
PDE11A-2-178494173-G>GGGA/GGGA	PDE11A	inframe	NA	-/S	NM_016953.3:c.2761_2763dupTCC	NM_016953.3	c.2761_2763dupTCC	NP_058649.3	NP_058649.3:p.Ser921dup
TTN-2-179566294-G>G/A	TTN	missense_variant	10187	P/S	NM_001267550.1:c.30559C>T	NM_001267550.1	c.30559C>T	NP_001254479.1	NP_001254479.1:p.Pro10187Ser
TTN-2-179577984-C>C/A	TTN	missense_variant	8959	W/C	NM_001267550.1:c.26877G>T	NM_001267550.1	c.26877G>T	NP_001254479.1	NP_001254479.1:p.Trp8959Cys
TTN-2-179587604-G>G/A	TTN	missense_variant	7341	S/F	NM_001267550.1:c.22022C>T	NM_001267550.1	c.22022C>T	NP_001254479.1	NP_001254479.1:p.Ser7341Phe
TTN-2-179598488-C>C/T	TTN	missense_variant	5210	E/K	NM_001267550.1:c.15628G>A	NM_001267550.1	c.15628G>A	NP_001254479.1	NP_001254479.1:p.Glu5210Lys
ZNF804A-2-185801756-G>G/A	ZNF804A	missense_variant	545	G/S	NM_194250.1:c.1633G>A	NM_194250.1	c.1633G>A	NP_919226.1	NP_919226.1:p.Gly545Ser
ZNF804A-2-185802211-C>C/CACA	ZNF804A	inframe	NA	-/T	NM_194250.1:c.2088_2089insACA	NM_194250.1	c.2088_2089insACA	NP_919226.1	NP_919226.1:p.Asn696_Thr697insThr
COL5A2-2-189898907-T>T/C	COL5A2	synonymous_variant	1463	E	NM_000393.3:c.4389A>G	NM_000393.3	c.4389A>G	NP_000384.2	NM_000393.3:c.4389A>G(p.=)
ALS2-2-202582895-A>A/C	ALS2	synonymous_variant	1247	G	NM_020919.3:c.3741T>G	NM_020919.3	c.3741T>G	NP_065970.2	NM_020919.3:c.3741T>G(p.=)
PARD3B-2-206023483-C>C/A	PARD3B	intron_variant	0	NA	NM_152526.5:c.1435-13452C>A	NM_152526.5	c.1435-13452C>A	NP_689739.4	NA
NRP2-2-206605371-C>C/T	NRP2	synonymous_variant	425	F	NM_201266.1:c.1275C>T	NM_201266.1	c.1275C>T	NP_957718.1	NM_201266.1:c.1275C>T(p.=)
NDUFS1-2-207012392-T>T/C	NDUFS1	intron_variant	0	NA	NM_001199984.1:c.463-7A>G	NM_001199984.1	c.463-7A>G	NP_001186913.1	NA
IDH1-2-209106871-TG>TG/T	IDH1	intron_variant	0	NA	NM_005896.2:c.699-3delC	NM_005896.2	c.699-3delC	NP_005887.2	NA
COL4A4-2-227945264-C>C/A	COL4A4	synonymous_variant	566	G	NM_000092.4:c.1698G>T	NM_000092.4	c.1698G>T	NP_000083.3	NM_000092.4:c.1698G>T(p.=)
SLC19A3-2-228560800-GA>G/G	SLC19A3	intron_variant	0	NA	NM_025243.3:c.980-4delT	NM_025243.3	c.980-4delT	NP_079519.1	NA

SP100-2-231405597-G>G/A	SP100	synonymous_variant	739	P	NM_001080391.1:c.2217G>A	NM_001080391.1	c.2217G>A	NP_001073860.1	NM_001080391.1:c.2217G>A(p.=)
GIGYF2-2-233704627-G>G/T	GIGYF2	synonymous_variant	966	S	NM_001103147.1:c.2898G>T	NM_001103147.1	c.2898G>T	NP_001096617.1	NM_001103147.1:c.2898G>T(p.=)
NEU2-2-233899077-C>C/T	NEU2	synonymous_variant	151	Y	NM_005383.2:c.453C>T	NM_005383.2	c.453C>T	NP_005374.2	NM_005383.2:c.453C>T(p.=)
COL6A3-2-238280651-G>G/A	COL6A3	missense_variant	1337	P/S	NM_004369.3:c.4009C>T	NM_004369.3	c.4009C>T	NP_004360.2	NP_004360.2:p.Pro1337Ser
ANO7-2-242157228-G>G/T	ANO7	synonymous_variant	754	P	NM_001001891.3:c.2262G>T	NM_001001891.3	c.2262G>T	NP_001001891.2	NM_001001891.3:c.2262G>T(p.=)
CIDEC-3-9912149-G>G/T	CIDEC	intron_variant	0	NA	NM_001199623.1:c.247-143C>A	NM_001199623.1	c.247-143C>A	NP_001186552.1	NA
ATP2B2-3-10413597-C>C/T	ATP2B2	missense_variant	519	V/I	NM_001001331.2:c.1555G>A	NM_001001331.2	c.1555G>A	NP_001001331.1	NP_001001331.1:p.Val519Ile
EOMES-3-27763427-G>G/GCGGCGC	EOMES	inframe	120	A/GAA	NM_005442.2:c.358_359insGCGCCG	NM_005442.2	c.358_359insGCGCCG	NP_005433.2	NP_005433.2:p.Ala120_Ala120insGlyAla
MLH1-3-37089131-A>A/G	MLH1	missense_variant	618	K/R	NM_000249.3:c.1853A>G	NM_000249.3	c.1853A>G	NP_000240.1	NP_000240.1:p.Lys618Arg
ABHD5-3-43743914-G>G/T	ABHD5	missense_variant	114	R/L	NM_016006.4:c.341G>T	NM_016006.4	c.341G>T	NP_057090.2	NP_057090.2:p.Arg114Leu
CCRL2-3-46450597-G>G/A	CCRL2	missense_variant	355	E/K	NM_001130910.1:c.1063G>A	NM_001130910.1	c.1063G>A	NP_001124382.1	NP_001124382.1:p.Glu355Lys
COL7A1-3-48618079-G>G/T	COL7A1	missense_variant	1663	P/T	NM_000094.3:c.4987C>A	NM_000094.3	c.4987C>A	NP_000085.1	NP_000085.1:p.Pro1663Thr
SLC26A6-3-48665978-G>G/A	SLC26A6	intron_variant	0	NA	NM_022911.2:c.1693-4C>T	NM_022911.2	c.1693-4C>T	NP_075062.2	NA
GPX1-3-49395673-GGCCGCC>G/GGCC	GPX1	inframe	NA	AAA/AA	NM_201397.1:c.33_38delGGCGGCinsGGC	NM_201397.1	c.33_38delGGCGGCinsGGC	NP_958799.1	NP_958799.1:p.Ala13del
AMT-3-49457752-G>G/A	AMT	synonymous_variant	121	N	NM_000481.3:c.363C>T	NM_000481.3	c.363C>T	NP_000472.2	NM_000481.3:c.363C>T(p.=)
MST1R-3-49934059-G>G/A	MST1R	missense_variant	785	H/Y	NM_002447.2:c.2353C>T	NM_002447.2	c.2353C>T	NP_002438.2	NP_002438.2:p.His785Tyr
RASSF1-3-50378090-G>G/A	RASSF1	synonymous_variant	49	G	NM_170714.1:c.147C>T	NM_170714.1	c.147C>T	NP_733832.1	NM_170714.1:c.147C>T(p.=)
DOCK3-3-51246259-C>C/G	DOCK3	synonymous_variant	364	S	NM_004947.4:c.1092C>G	NM_004947.4	c.1092C>G	NP_004938.1	NM_004947.4:c.1092C>G(p.=)
ITIH1-3-52825833-A>A/G	ITIH1	missense_variant	881	H/R	NM_002215.3:c.2642A>G	NM_002215.3	c.2642A>G	NP_002206.2	NP_002206.2:p.His881Arg
CHDH-3-53853074-G>G/T	CHDH	intron_variant	0	NA	NM_018397.4:c.1264-7C>A	NM_018397.4	c.1264-7C>A	NP_060867.2	NA
CHDH-3-53853075-A>A/T	CHDH	intron_variant	0	NA	NM_018397.4:c.1264-8T>A	NM_018397.4	c.1264-8T>A	NP_060867.2	NA
ARF4-3-57563120-GA>GA/G	ARF4	intron_variant	0	NA	NM_001660.3:c.259-7delT	NM_001660.3	c.259-7delT	NP_001651.1	NA
DNASE1L3-3-58196522-C>C/G	DNASE1L3	missense_variant	38	D/H	NM_004944.3:c.112G>C	NM_004944.3	c.112G>C	NP_004935.1	NP_004935.1:p.Asp38His
MITF-3-69928279-C>C/T	MITF	intron_variant	0	NA	NM_198159.2:c.105-6C>T	NM_198159.2	c.105-6C>T	NP_937802.1	NA
MYH15-3-108102586-T>A/A	MYH15	intron_variant	0	NA	NM_014981.1:c.5692-10A>T	NM_014981.1	c.5692-10A>T	NP_055796.1	NA
CD96-3-111298000-G>G/A	CD96	missense_variant	240	D/N	NM_198196.2:c.718G>A	NM_198196.2	c.718G>A	NP_937839.1	NP_937839.1:p.Asp240Asn
ARHGAP31-3-119102053-C>C/T	ARHGAP31	missense_variant	221	P/L	NM_020754.2:c.662C>T	NM_020754.2	c.662C>T	NP_065805.2	NP_065805.2:p.Pro221Leu
ILDR1-3-121724098-G>G/A	ILDR1	synonymous_variant	124	I	NM_001199799.1:c.372C>T	NM_001199799.1	c.372C>T	NP_001186728.1	NM_001199799.1:c.372C>T(p.=)
MUC13-3-124646705-A>AAAG/AAAG	MUC13	inframe	62	F/SF	NM_033049.3:c.182_184dupCTT	NM_033049.3	c.182_184dupCTT	NP_149038.3	NP_149038.3:p.Phe62_Pro62insSer
COL6A4P2-3-129982997-CT>CT/C	COL6A4P2	nc_exon_variant	0	NA	NR_027898.1:n.1942delT	NR_027898.1	n.1942delT	NA	NA
COL6A5-3-130095464-A>A/C	COL6A5	missense_variant	151	E/A	NM_153264.5:c.452A>C	NM_153264.5	c.452A>C	NP_694996.5	NP_694996.5:p.Glu151Ala
TOPBP1-3-133320222-C>C/T	TOPBP1	missense_variant	1481	V/I	NM_007027.3:c.4441G>A	NM_007027.3	c.4441G>A	NP_008958.2	NP_008958.2:p.Val1481Ile
ATR-3-142274770-T>T/C	ATR	missense_variant	764	K/E	NM_001184.3:c.2290A>G	NM_001184.3	c.2290A>G	NP_001175.2	NP_001175.2:p.Lys764Glu
SI-3-164733763-C>C/T	SI	missense_variant	1289	G/R	NM_001041.3:c.3865G>A	NM_001041.3	c.3865G>A	NP_001032.2	NP_001032.2:p.Gly1289Arg
SI-3-164792451-A>A/G	SI	synonymous_variant	41	I	NM_001041.3:c.123T>C	NM_001041.3	c.123T>C	NP_001032.2	NM_001041.3:c.123T>C(p.=)
SERPINI2-3-167167141-C>C/T	SERPINI2	synonymous_variant	338	E	NM_006217.3:c.1014G>A	NM_006217.3	c.1014G>A	NP_006208.1	NM_006217.3:c.1014G>A(p.=)
EIF4G1-3-184045777-C>C/T	EIF4G1	stop_gained	1321	Q/*	NM_001194947.1:c.3961C>T	NM_001194947.1	c.3961C>T	NP_001181876.1	NP_001181876.1:p.Gln1321Ter
TM4SF19-3-196050620-C>C/T	TM4SF19	missense_variant	232	E/K	NM_001204897.1:c.694G>A	NM_001204897.1	c.694G>A	NP_001191826.1	NP_001191826.1:p.Glu232Lys
HTT-4-3208217-C>C/T	HTT	intron_variant	0	NA	NM_002111.6:c.5719-6C>T	NM_002111.6	c.5719-6C>T	NP_002102.4	NA
LRPAP1-4-3519881-C>C/T	LRPAP1	missense_variant	211	D/N	NM_002337.3:c.631G>A	NM_002337.3	c.631G>A	NP_002328.1	NP_002328.1:p.Asp211Asn

SLC34A2-4-25677884-C>C/T	SLC34A2	missense_variant	529	A/V	NM_006424.2:c.1586C>T	NM_006424.2	c.1586C>T	NP_006415.2	NP_006415.2:p.Ala529Val
SLC34A2-4-25677885-C>C/T	SLC34A2	synonymous_variant	529	A	NM_006424.2:c.1587C>T	NM_006424.2	c.1587C>T	NP_006415.2	NM_006424.2:c.1587C>T(p.=)
UGT2B7-4-69962700-T>T/C	UGT2B7	synonymous_variant	154	F	NM_001074.2:c.462T>C	NM_001074.2	c.462T>C	NP_001065.2	NM_001074.2:c.462T>C(p.=)
MUC7-4-71347185-T>T/C	MUC7	missense_variant	242	S/P	NM_001145006.1:c.724T>C	NM_001145006.1	c.724T>C	NP_001138478.1	NP_001138478.1:p.Ser242Pro
MUC7-4-71347207-T>T/C	MUC7	missense_variant	249	L/P	NM_001145006.1:c.746T>C	NM_001145006.1	c.746T>C	NP_001138478.1	NP_001138478.1:p.Leu249Pro
PTPN13-4-87692632-T>T/A	PTPN13	synonymous_variant	1709	I	NM_080685.2:c.5127T>A	NM_080685.2	c.5127T>A	NP_542416.1	NM_080685.2:c.5127T>A(p.=)
PTPN13-4-87692633-T>T/C	PTPN13	missense_variant	1710	C/R	NM_080685.2:c.5128T>C	NM_080685.2	c.5128T>C	NP_542416.1	NP_542416.1:p.Cys1710Arg
RRH-4-110763764-C>C/T	RRH	missense_variant	287	T/I	NM_006583.2:c.860C>T	NM_006583.2	c.860C>T	NP_006574.1	NP_006574.1:p.Thr287Ile
MGST2-4-140616417-C>C/T	MGST2	synonymous_variant	75	N	NM_002413.4:c.225C>T	NM_002413.4	c.225C>T	NP_002404.1	NM_002413.4:c.225C>T(p.=)
GYPA-4-145041720-A>G/G	GYPA	missense_variant	20	L/S	NM_002099.6:c.59T>C	NM_002099.6	c.59T>C	NP_002090.4	NP_002090.4:p.Leu20Ser
FGG-4-155533035-G>G/C	FGG	missense_variant	108	A/G	NM_021870.2:c.323C>G	NM_021870.2	c.323C>G	NP_068656.2	NP_068656.2:p.Ala108Gly
FRG1-4-190876196-G>G/A	FRG1	missense_variant	108	A/T	NM_004477.2:c.322G>A	NM_004477.2	c.322G>A	NP_004468.1	NP_004468.1:p.Ala108Thr
FRG1-4-190876204-G>G/A	FRG1	synonymous_variant	110	K	NM_004477.2:c.330G>A	NM_004477.2	c.330G>A	NP_004468.1	NM_004477.2:c.330G>A(p.=)
FRG1-4-190876242-G>G/A	FRG1	missense_variant	123	G/E	NM_004477.2:c.368G>A	NM_004477.2	c.368G>A	NP_004468.1	NP_004468.1:p.Gly123Glu
FRG1-4-190876257-G>G/A	FRG1	missense_variant	128	R/H	NM_004477.2:c.383G>A	NM_004477.2	c.383G>A	NP_004468.1	NP_004468.1:p.Arg128His
FRG1-4-190882973-A>A/G	FRG1	intron_variant	0	NA	NM_004477.2:c.630-4A>G	NM_004477.2	c.630-4A>G	NP_004468.1	NA
FRG1-4-190883026-G>G/T	FRG1	missense_variant	227	D/Y	NM_004477.2:c.679G>T	NM_004477.2	c.679G>T	NP_004468.1	NP_004468.1:p.Asp227Tyr
FRG1-4-190883051-G>G/A	FRG1	missense_variant	235	R/Q	NM_004477.2:c.704G>A	NM_004477.2	c.704G>A	NP_004468.1	NP_004468.1:p.Arg235Gln
SLC6A18-5-1235627-C>C/T	SLC6A18	synonymous_variant	157	A	NM_182632.2:c.471C>T	NM_182632.2	c.471C>T	NP_872438.2	NM_182632.2:c.471C>T(p.=)
DNAH5-5-13776635-G>G/A	DNAH5	stop_gained	3096	R/*	NM_001369.2:c.9286C>T	NM_001369.2	c.9286C>T	NP_001360.1	NP_001360.1:p.Arg3096Ter
DNAH5-5-13777417-C>C/T	DNAH5	missense_variant	3000	R/Q	NM_001369.2:c.8999G>A	NM_001369.2	c.8999G>A	NP_001360.1	NP_001360.1:p.Arg3000Gln
PRDM9-5-23527565-A>A/C	PRDM9	missense_variant	790	N/H	NM_020227.2:c.2368A>C	NM_020227.2	c.2368A>C	NP_064612.2	NP_064612.2:p.Asn790His
NIPBL-5-37019434-A>A/G	NIPBL	missense_variant	1648	I/V	NM_133433.3:c.4942A>G	NM_133433.3	c.4942A>G	NP_597677.2	NP_597677.2:p.Ile1648Val
LIFR-5-38481813-G>G/A	LIFR	missense_variant	1060	P/S	NM_001127671.1:c.3178C>T	NM_001127671.1	c.3178C>T	NP_001121143.1	NP_001121143.1:p.Pro1060Ser
LIFR-5-38485971-T>T/C	LIFR	missense_variant	816	D/G	NM_001127671.1:c.2447A>G	NM_001127671.1	c.2447A>G	NP_001121143.1	NP_001121143.1:p.Asp816Gly
MAP3K1-5-56177848-TCAA>T/T	MAP3K1	inframe	NA	ST/S	NM_005921.1:c.2822_2824delCAA	NM_005921.1	c.2822_2824delCAA	NP_005912.1	NP_005912.1:p.Thr942del
CDK7-5-68550423-A>A/G	CDK7	intron_variant	0	NA	NM_001799.3:c.161-6A>G	NM_001799.3	c.161-6A>G	NP_001790.1	NA
MCCC2-5-70952652-A>A/G	MCCC2	missense_variant	553	I/V	NM_022132.4:c.1657A>G	NM_022132.4	c.1657A>G	NP_071415.1	NP_071415.1:p.Ile553Val
AP3B1-5-77335015-G>G/T	AP3B1	missense_variant	887	F/L	NM_003664.3:c.2661C>A	NM_003664.3	c.2661C>A	NP_003655.3	NP_003655.3:p.Phe887Leu
WDR36-5-110434448-C>C/T	WDR36	missense_variant	163	A/V	NM_139281.2:c.488C>T	NM_139281.2	c.488C>T	NP_644810.1	NP_644810.1:p.Ala163Val
SPINK5-5-147505328-C>C/A	SPINK5	missense_variant	958	L/I	NM_001127698.1:c.2872C>A	NM_001127698.1	c.2872C>A	NP_001121170.1	NP_001121170.1:p.Leu958Ile
SH3TC2-5-148417937-A>A/G	SH3TC2	missense_variant	308	F/L	NM_024577.3:c.922T>C	NM_024577.3	c.922T>C	NP_078853.2	NP_078853.2:p.Phe308Leu
ADAM19-5-156945833-C>C/G	ADAM19	missense_variant	222	E/Q	NM_033274.3:c.664G>C	NM_033274.3	c.664G>C	NP_150377.1	NP_150377.1:p.Glu222Gln
ADAM19-5-156991445-G>G/A	ADAM19	missense_variant	63	L/F	NM_033274.3:c.187C>T	NM_033274.3	c.187C>T	NP_150377.1	NP_150377.1:p.Leu63Phe
WWC1-5-167850877-C>C/T	WWC1	synonymous_variant	538	S	NM_001161661.1:c.1614C>T	NM_001161661.1	c.1614C>T	NP_001155133.1	NM_001161661.1:c.1614C>T(p.=)
GRM6-5-178413725-G>G/A	GRM6	synonymous_variant	510	P	NM_000843.3:c.1530C>T	NM_000843.3	c.1530C>T	NP_000834.2	NM_000843.3:c.1530C>T(p.=)
GRM6-5-178418906-C>C/T	GRM6	missense_variant	228	S/N	NM_000843.3:c.683G>A	NM_000843.3	c.683G>A	NP_000834.2	NP_000834.2:p.Ser228Asn
ADAMTS2-5-178581083-C>C/T	ADAMTS2	missense_variant	450	R/H	NM_014244.4:c.1349G>A	NM_014244.4	c.1349G>A	NP_055059.2	NP_055059.2:p.Arg450His
FOXC1-6-1612017-A>A/ACGG	FOXC1	inframe	446	H/HG	NM_001453.2:c.1337_1338insCGG	NM_001453.2	c.1337_1338insCGG	NP_001444.2	NP_001444.2:p.His446_Gly447insGly
HLA-A-6-29910716-C>C/G	HLA-A	missense_variant	86	Q/E	NM_002116.7:c.256C>G	NM_002116.7	c.256C>G	NP_002107.3	NP_002107.3:p.Gln86Glu
HLA-A-6-29910717-A>A/G	HLA-A	missense_variant	86	Q/R	NM_002116.7:c.257A>G	NM_002116.7	c.257A>G	NP_002107.3	NP_002107.3:p.Gln86Arg
HLA-A-6-29911893-C>C/T	HLA-A	intron_variant	0	NA	NM_002116.7:c.620-6C>T	NM_002116.7	c.620-6C>T	NP_002107.3	NA
HLA-A-6-29912108-G>G/C	HLA-A	missense_variant	277	E/Q	NM_002116.7:c.829G>C	NM_002116.7	c.829G>C	NP_002107.3	NP_002107.3:p.Glu277Gln
HLA-A-6-29912856-A>A/T	HLA-A	missense_variant	345	T/S	NM_002116.7:c.1033A>T	NM_002116.7	c.1033A>T	NP_002107.3	NP_002107.3:p.Thr345Ser

CCHCR1-6-31113276-G>G/A	CCHCR1	intron_variant	0	NA	NM_001105564.1:c.1694-6C>T	NM_001105564.1	c.1694-6C>T	NP_001099034.1	NA
HLA-C-6-31237769-G>G/A	HLA-C	missense_variant	330	A/V	NM_002117.5:c.989C>T	NM_002117.5	c.989C>T	NP_002108.4	NP_002108.4:p.Ala330Val
HLA-C-6-31237774-G>G/C	HLA-C	synonymous_variant	328	V	NM_002117.5:c.984C>G	NM_002117.5	c.984C>G	NP_002108.4	NM_002117.5:c.984C>G(p.=)
MICA-6-31379817-T>C/C	MICA	missense_variant	236	I/T	NM_001177519.1:c.707T>C	NM_001177519.1	c.707T>C	NP_001170990.1	NP_001170990.1:p.Ile236Thr
MICA-6-31379931-G>A/A	MICA	missense_variant	274	R/Q	NM_001177519.1:c.821G>A	NM_001177519.1	c.821G>A	NP_001170990.1	NP_001170990.1:p.Arg274Gln
MICA-6-31380157-TGCTG>TGCTG/T	MICA	frameshift_variant	NA	NA	NM_001177519.1:c.949_952delGCTG	NM_001177519.1	c.949_952delGCTG	NP_001170990.1	NP_001170990.1:p.Gly318AlafsTer67
MICA-6-31380161-G>G/GCT	MICA	frameshift_variant	318	NA	NM_001177519.1:c.952_953insCT	NM_001177519.1	c.952_953insCT	NP_001170990.1	NP_001170990.1:p.Gly318AlafsTer69
NCR3-6-31556992-G>G/C	NCR3	intron_variant	0	NA	NM_147130.2:c.497-39C>G	NM_147130.2	c.497-39C>G	NP_667341.1	NA
HSPA1L-6-31778948-C>C/T	HSPA1L	missense_variant	268	A/T	NM_005527.3:c.802G>A	NM_005527.3	c.802G>A	NP_005518.3	NP_005518.3:p.Ala268Thr
TNXA-6-31977552-C>C/G	TNXA	nc_exon_variant	0	NA	NR_001284.2:n.2010G>C	NR_001284.2	n.2010G>C	NA	NA
CYP21A2-6-32006867-G>G/A	CYP21A2	intron_variant	0	NA	NM_000500.7:c.293-4G>A	NM_000500.7	c.293-4G>A	NP_000491.4	NA
CYP21A2-6-32008451-C>C/T	CYP21A2	synonymous_variant	375	S	NM_000500.7:c.1125C>T	NM_000500.7	c.1125C>T	NP_000491.4	NM_000500.7:c.1125C>T(p.=)
HLA-DRB5-6-32489671-A>G/G	HLA-DRB5	intron_variant	0	NA	NM_002125.3:c.370+11T>C	NM_002125.3	c.370+11T>C	NP_002116.2	NA
HLA-DRB5-6-32489672-C>C/A	HLA-DRB5	intron_variant	0	NA	NM_002125.3:c.370+10G>T	NM_002125.3	c.370+10G>T	NP_002116.2	NA
HLA-DRB5-6-32489674-A>A/G	HLA-DRB5	intron_variant	0	NA	NM_002125.3:c.370+8T>C	NM_002125.3	c.370+8T>C	NP_002116.2	NA
HLA-DRB5-6-32489675-T>T/C	HLA-DRB5	intron_variant	0	NA	NM_002125.3:c.370+7A>G	NM_002125.3	c.370+7A>G	NP_002116.2	NA
HLA-DRB5-6-32489766-A>A/T	HLA-DRB5	missense_variant	96	F/I	NM_002125.3:c.286T>A	NM_002125.3	c.286T>A	NP_002116.2	NP_002116.2:p.Phe96Ile
HLA-DRB5-6-32489791-A>G/C	HLA-DRB5	synonymous_variant	87	A	NM_002125.3:c.261T>G	NM_002125.3	c.261T>G	NP_002116.2	NM_002125.3:c.261T>G(p.=)
HLA-DRB5-6-32489792-G>G/T	HLA-DRB5	missense_variant	87	A/D	NM_002125.3:c.260C>A	NM_002125.3	c.260C>A	NP_002116.2	NP_002116.2:p.Ala87Asp
HLA-DRB5-6-32489795-T>T/C	HLA-DRB5	missense_variant	86	D/G	NM_002125.3:c.257A>G	NM_002125.3	c.257A>G	NP_002116.2	NP_002116.2:p.Asp86Gly
HLA-DRB5-6-32489796-C>C/T	HLA-DRB5	missense_variant	86	D/N	NM_002125.3:c.256G>A	NM_002125.3	c.256G>A	NP_002116.2	NP_002116.2:p.Asp86Asn
HLA-DRB1-6-32548026-G>G/C	HLA-DRB1	missense_variant	262	T/R	NM_002124.3:c.785C>G	NM_002124.3	c.785C>G	NP_002115.2	NP_002115.2:p.Thr262Arg
HLA-DRB1-6-32552059-G>T/A	HLA-DRB1	missense_variant	66	S/F	NM_002124.3:c.197C>T	NM_002124.3	c.197C>T	NP_002115.2	NP_002115.2:p.Ser66Phe
HLA-DQB1-6-32632781-A>T/T	HLA-DQB1	missense_variant	58	L/H	NM_001243961.1:c.173T>A	NM_001243961.1	c.173T>A	NP_001230890.1	NP_001230890.1:p.Leu58His
HLA-DPB1-6-33048689-G>A/A	HLA-DPB1	missense_variant	114	G/E	NM_002121.5:c.341G>A	NM_002121.5	c.341G>A	NP_002112.3	NP_002112.3:p.Gly114Glu
HLA-DPB1-6-33048691-C>G/G	HLA-DPB1	missense_variant	115	P/A	NM_002121.5:c.343C>G	NM_002121.5	c.343C>G	NP_002112.3	NP_002112.3:p.Pro115Ala
HLA-DPB1-6-33052717-A>C/C	HLA-DPB1	intron_variant	0	NA	NM_002121.5:c.365-10A>C	NM_002121.5	c.365-10A>C	NP_002112.3	NA
HLA-DPB1-6-33052719-G>A/A	HLA-DPB1	intron_variant	0	NA	NM_002121.5:c.365-8G>A	NM_002121.5	c.365-8G>A	NP_002112.3	NA
HLA-DPB1-6-33052723-C>G/G	HLA-DPB1	intron_variant	0	NA	NM_002121.5:c.365-4C>G	NM_002121.5	c.365-4C>G	NP_002112.3	NA
HLA-DPB1-6-33052736-G>A/A	HLA-DPB1	missense_variant	125	R/K	NM_002121.5:c.374G>A	NM_002121.5	c.374G>A	NP_002112.3	NP_002112.3:p.Arg125Lys
HLA-DPB1-6-33052743-T>C/C	HLA-DPB1	synonymous_variant	127	N	NM_002121.5:c.381T>C	NM_002121.5	c.381T>C	NP_002112.3	NM_002121.5:c.381T>C(p.=)
PNPLA1-6-36238293-G>G/A	PNPLA1	synonymous_variant	19	S	NM_001145717.1:c.57G>A	NM_001145717.1	c.57G>A	NP_001139189.2	NM_001145717.1:c.57G>A(p.=)
PNPLA1-6-36275394-G>G/A	PNPLA1	synonymous_variant	500	V	NM_001145717.1:c.1500G>A	NM_001145717.1	c.1500G>A	NP_001139189.2	NM_001145717.1:c.1500G>A(p.=)
TRERF1-6-42200562-C>C/T	TRERF1	synonymous_variant	1045	K	NM_033502.2:c.3135G>A	NM_033502.2	c.3135G>A	NP_277037.1	NM_033502.2:c.3135G>A(p.=)
PRPH2-6-42689824-G>G/A	PRPH2	synonymous_variant	83	Y	NM_000322.4:c.249C>T	NM_000322.4	c.249C>T	NP_000313.2	NM_000322.4:c.249C>T(p.=)
CUL7-6-43018819-G>G/A	CUL7	missense_variant	458	R/W	NM_001168370.1:c.1372C>T	NM_001168370.1	c.1372C>T	NP_001161842.1	NP_001161842.1:p.Arg458Trp
IL17F-6-52101743-G>G/A	IL17F	missense_variant	160	H/Y	NM_052872.3:c.478C>T	NM_052872.3	c.478C>T	NP_443104.1	NP_443104.1:p.His160Tyr
EFHC1-6-52358887-T>T/TCA	EFHC1	3_prime_UTR_variant	0	NA	NM_018100.3:c.*1748_*1749insCA	NM_018100.3	c.*1748_*1749insCA	NP_060570.2	NA
MYO6-6-76624538-G>G/A	MYO6	missense_variant	1223	D/N	NM_004999.3:c.3667G>A	NM_004999.3	c.3667G>A	NP_004990.3	NP_004990.3:p.Asp1223Asn
BCKDHB-6-80837253-G>G/T	BCKDHB	intron_variant	0	NA	NM_000056.3:c.197-11G>T	NM_000056.3	c.197-11G>T	NP_000047.1	NA
TSPYL1-6-116600465-T>T/TCAC	TSPYL1	inframe	NA	-/V	NM_003309.3:c.526_528dupGTG	NM_003309.3	c.526_528dupGTG	NP_003300.1	NP_003300.1:p.Val176dup
MYB-6-135516912-C>C/T	MYB	synonymous_variant	325	P	NM_001130173.1:c.975C>T	NM_001130173.1	c.975C>T	NP_001123645.1	NM_001130173.1:c.975C>T(p.=)

TNFAIP3-6-138199814-G>G/A	TNFAIP3	missense_variant	411	R/Q	NM_006290.3:c.1232G>A	NM_006290.3	c.1232G>A	NP_006281.1	NP_006281.1:p.Arg411Gln
TNFAIP3-6-138199898-G>G/C	TNFAIP3	missense_variant	439	R/P	NM_006290.3:c.1316G>C	NM_006290.3	c.1316G>C	NP_006281.1	NP_006281.1:p.Arg439Pro
SYNE1-6-152697675-G>G/T	SYNE1	synonymous_variant	3055	S	NM_182961.3:c.9165C>A	NM_182961.3	c.9165C>A	NP_892006.3	NM_182961.3:c.9165C>A(p.=)
PLG-6-161152905-C>C/T	PLG	missense_variant	523	R/W	NM_000301.3:c.1567C>T	NM_000301.3	c.1567C>T	NP_000292.1	NP_000292.1:p.Arg523Trp
PMS2-7-6036969-T>T/C	PMS2	missense_variant	264	H/R	NM_000535.5:c.791A>G	NM_000535.5	c.791A>G	NP_000526.1	NP_000526.1:p.His264Arg
BMPER-7-33945364-G>G/A	BMPER	intron_variant	0	NA	NM_133468.4:c.133+6G>A	NM_133468.4	c.133+6G>A	NP_597725.1	NA
NPSR1-AS1.NPSR1-7-34818119-G>G/A	NPSR1-AS1.NPSR1	missense_variant	109	R/Q	NM_207173.1:c.326G>A	NM_207173.1	c.326G>A	NP_997056.1	NP_997056.1:p.Arg109Gln
PGAM2-7-44104754-C>C/T	PGAM2	synonymous_variant	125	P	NM_000290.3:c.375G>A	NM_000290.3	c.375G>A	NP_000281.2	NM_000290.3:c.375G>A(p.=)
ZPBP-7-50121384-C>C/T	ZPBP	missense_variant	107	G/E	NM_007009.2:c.320G>A	NM_007009.2	c.320G>A	NP_008940.2	NP_008940.2:p.Gly107Glu
NCF1-7-74193668-G>A/A	NCF1	missense_variant	99	G/S	NM_000265.4:c.295G>A	NM_000265.4	c.295G>A	NP_000256.3	NP_000256.3:p.Gly99Ser
GTF2IRD2-7-74212036-C>C/G	GTF2IRD2	missense_variant	605	K/N	NM_173537.2:c.1815G>C	NM_173537.2	c.1815G>C	NP_775808.2	NP_775808.2:p.Lys605Asn
GTF2IRD2-7-74212351-C>C/T	GTF2IRD2	synonymous_variant	500	L	NM_173537.2:c.1500G>A	NM_173537.2	c.1500G>A	NP_775808.2	NM_173537.2:c.1500G>A(p.=)
GTF2IRD2-7-74212352-A>A/G	GTF2IRD2	missense_variant	500	L/S	NM_173537.2:c.1499T>C	NM_173537.2	c.1499T>C	NP_775808.2	NP_775808.2:p.Leu500Ser
PMS2P3-7-75145411-T>C/C	PMS2P3	intron_variant	0	NA	NR_028059.1:n.745+9A>G	NR_028059.1	n.745+9A>G	NA	NA
PMS2P3-7-75157166-CCTTCGCCTCCGCCGCCA>CCTTCGCCTCCGCCGCCA/C	PMS2P3	nc_exon_variant	0	NA	NR_028059.1:n.271_287delTGGCGGCGGAGGCGAAG	NR_028059.1	n.271_287delTGGCGGCGGAGGCGAAG	NA	NA
GRM3-7-86394940-C>C/G	GRM3	intron_variant	0	NA	NM_000840.2:c.468+11C>G	NM_000840.2	c.468+11C>G	NP_000831.2	NA
DLX5-7-96650226-C>C/T	DLX5	missense_variant	231	R/H	NM_005221.5:c.692G>A	NM_005221.5	c.692G>A	NP_005212.1	NP_005212.1:p.Arg231His
AP4M1-7-99700378-C>C/T	AP4M1	synonymous_variant	76	P	NM_004722.3:c.228C>T	NM_004722.3	c.228C>T	NP_004713.2	NM_004722.3:c.228C>T(p.=)
ZAN-7-100371473-C>C/T	ZAN	missense_variant	1922	R/C	NM_003386.1:c.5764C>T	NM_003386.1	c.5764C>T	NP_003377.1	NP_003377.1:p.Arg1922Cys
ZAN-7-100371474-G>G/A	ZAN	missense_variant	1922	R/H	NM_003386.1:c.5765G>A	NM_003386.1	c.5765G>A	NP_003377.1	NP_003377.1:p.Arg1922His
MUC3A-7-100610069-C>C/T	MUC3A	missense_variant	1462	H/Y	XM_001725354.4:c.4384C>T	XM_001725354.4	c.4384C>T	XP_001725406.2	XP_001725406.2:p.His1462Tyr
LAMB1-7-107564539-G>A/A	LAMB1	intron_variant	0	NA	NM_002291.2:c.5225-7C>T	NM_002291.2	c.5225-7C>T	NP_002282.2	NA
LAMB1-7-107564542-G>T/T	LAMB1	intron_variant	0	NA	NM_002291.2:c.5225-10C>A	NM_002291.2	c.5225-10C>A	NP_002282.2	NA
FOXP2-7-114331998-G>G/A	FOXP2	3_prime_UTR_variant	0	NA	NM_148898.3:c.*2017G>A	NM_148898.3	c.*2017G>A	NP_683696.2	NA
CEP41-7-130036343-C>C/T	CEP41	3_prime_UTR_variant	0	NA	NM_018718.2:c.*2389G>A	NM_018718.2	c.*2389G>A	NP_061188.1	NA
CEP41-7-130038583-A>A/G	CEP41	3_prime_UTR_variant	0	NA	NM_018718.2:c.*149T>C	NM_018718.2	c.*149T>C	NP_061188.1	NA
CEP41-7-130080985-G>A/T	CEP41	5_prime_UTR_variant	0	NA	NM_018718.2:c.-178C>A	NM_018718.2	c.-178C>A	NP_061188.1	NA
CHRM2-7-136700569-C>C/T	CHRM2	synonymous_variant	319	N	NM_001006627.1:c.957C>T	NM_001006627.1	c.957C>T	NP_001006628.1	NM_001006627.1:c.957C>T(p.=)
PRSS1-7-142460313-T>T/C	PRSS1	synonymous_variant	162	D	NM_002769.4:c.486T>C	NM_002769.4	c.486T>C	NP_002760.1	NM_002769.4:c.486T>C(p.=)
KEL-7-142658471-A>A/G	KEL	missense_variant	67	Y/H	NM_000420.2:c.199T>C	NM_000420.2	c.199T>C	NP_000411.1	NP_000411.1:p.Tyr67His
CNTNAP2-7-148106477-C>C/CTG	CNTNAP2	intron_variant	0	NA	NM_014141.5:c.3716-6_3716-5insTG	NM_014141.5	c.3716-6_3716-5insTG	NP_054860.1	NA
KMT2C-7-151932908-T>T/C	KMT2C	synonymous_variant	921	L	NM_170606.2:c.2763A>G	NM_170606.2	c.2763A>G	NP_733751.2	NM_170606.2:c.2763A>G(p.=)
KMT2C-7-151935798-G>G/T	KMT2C	synonymous_variant	882	I	NM_170606.2:c.2646C>A	NM_170606.2	c.2646C>A	NP_733751.2	NM_170606.2:c.2646C>A(p.=)
KMT2C-7-151935799-A>A/G	KMT2C	missense_variant	882	I/T	NM_170606.2:c.2645T>C	NM_170606.2	c.2645T>C	NP_733751.2	NP_733751.2:p.Ile882Thr
KMT2C-7-151935866-G>G/A	KMT2C	missense_variant	860	P/S	NM_170606.2:c.2578C>T	NM_170606.2	c.2578C>T	NP_733751.2	NP_733751.2:p.Pro860Ser
KMT2C-7-151935871-C>C/A	KMT2C	missense_variant	858	W/L	NM_170606.2:c.2573G>T	NM_170606.2	c.2573G>T	NP_733751.2	NP_733751.2:p.Trp858Leu
DPP6-7-154143378-C>C/T	DPP6	missense_variant	44	S/F		NM_001039350		NA	NA
CSMD1-8-3165949-G>G/C	CSMD1	synonymous_variant	1236	T	NM_033225.5:c.3708C>G	NM_033225.5	c.3708C>G	NP_150094.5	NM_033225.5:c.3708C>G(p.=)
GATA4-8-11614524-G>G/C	GATA4	missense_variant	360	E/Q	NM_002052.3:c.1078G>C	NM_002052.3	c.1078G>C	NP_002043.2	NP_002043.2:p.Glu360Gln

NEIL2-8-11637247-C>C/T	NEIL2	synonymous_variant	93	S	NM_001135746.1:c.279C>T	NM_001135746.1	c.279C>T	NP_001129218.1	NM_001135746.1:c.279C>T(p.=)
MTUS1-8-17581312-G>G/A	MTUS1	missense_variant	773	S/L	NM_001001924.2:c.2318C>T	NM_001001924.2	c.2318C>T	NP_001001924.1	NP_001001924.1:p.Ser773Leu
LOXL2-8-23198540-G>G/A	LOXL2	synonymous_variant	236	F	NM_002318.2:c.708C>T	NM_002318.2	c.708C>T	NP_002309.1	NM_002318.2:c.708C>T(p.=)
NEFL-8-24813363-G>G/A	NEFL	synonymous_variant	223	L	NM_006158.3:c.667C>T	NM_006158.3	c.667C>T	NP_006149.2	NM_006158.3:c.667C>T(p.=)
CLU-8-27455628-G>G/A	CLU	3_prime_UTR_variant	0	NA	NM_001831.3:c.*130C>T	NM_001831.3	c.*130C>T	NP_001822.3	NA
FGFR1-8-38297874-C>C/T	FGFR1	intron_variant	0	NA	NM_001174067.1:c.191-10408G>A	NM_001174067.1	c.191-10408G>A	NP_001167538.1	NA
ANK1-8-41552304-C>C/T	ANK1	missense_variant	1086	E/K	NM_001142446.1:c.3256G>A	NM_001142446.1	c.3256G>A	NP_001135918.1	NP_001135918.1:p.Glu1086Lys
PRKDC-8-48696360-G>G/A	PRKDC	missense_variant	3706	T/M	NM_006904.6:c.11117C>T	NM_006904.6	c.11117C>T	NP_008835.5	NP_008835.5:p.Thr3706Met
TTPA-8-63978658-TA>T/T	TTPA	intron_variant	0	NA	NM_000370.3:c.359-3delT	NM_000370.3	c.359-3delT	NP_000361.1	NA
ZFHX4-8-77764310-T>T/C	ZFHX4	missense_variant	1718	F/S	NM_024721.4:c.5153T>C	NM_024721.4	c.5153T>C	NP_078997.4	NP_078997.4:p.Phe1718Ser
CA2-8-86377539-G>G/A	CA2	missense_variant	25	G/R	NM_000067.2:c.73G>A	NM_000067.2	c.73G>A	NP_000058.1	NP_000058.1:p.Gly25Arg
UQCRB-8-97242133-ACT>ACT/A	UQCRB	3_prime_UTR_variant	0	NA	NM_001254752.1:c.*1198_*1199delAG	NM_001254752.1	c.*1198_*1199delAG	NP_001241681.1	NA
VPS13B-8-100844845-C>C/T	VPS13B	synonymous_variant	3218	P	NM_017890.4:c.9654C>T	NM_017890.4	c.9654C>T	NP_060360.3	NM_017890.4:c.9654C>T(p.=)
TAF2-8-120816184-C>C/T	TAF2	missense_variant	165	S/N	NM_003184.3:c.494G>A	NM_003184.3	c.494G>A	NP_003175.1	NP_003175.1:p.Ser165Asn
TG-8-133899575-G>G/A	TG	missense_variant	653	G/D	NM_003235.4:c.1958G>A	NM_003235.4	c.1958G>A	NP_003226.4	NP_003226.4:p.Gly653Asp
TG-8-134125778-G>G/A	TG	stop_gained	2562	W/*	NM_003235.4:c.7685G>A	NM_003235.4	c.7685G>A	NP_003226.4	NP_003226.4:p.Trp2562Ter
TRAPPC9-8-141294071-G>G/C	TRAPPC9	missense_variant	775	N/K	NM_031466.5:c.2325C>G	NM_031466.5	c.2325C>G	NP_113654.4	NP_113654.4:p.Asn775Lys
ZC3H3-8-144522426-G>G/A	ZC3H3	missense_variant	867	P/L	NM_015117.2:c.2600C>T	NM_015117.2	c.2600C>T	NP_055932.2	NP_055932.2:p.Pro867Leu
PLEC-8-145007478-C>C/T	PLEC	synonymous_variant	572	A	NM_201380.2:c.1716G>A	NM_201380.2	c.1716G>A	NP_958782.1	NM_201380.2:c.1716G>A(p.=)
SLC1A1-9-4567705-G>G/A	SLC1A1	missense_variant	174	D/N	NM_004170.5:c.520G>A	NM_004170.5	c.520G>A	NP_004161.4	NP_004161.4:p.Asp174Asn
SLC1A1-9-4573903-C>C/T	SLC1A1	intron_variant	0	NA	NM_004170.5:c.768-4C>T	NM_004170.5	c.768-4C>T	NP_004161.4	NA
JAK2-9-5054648-C>C/T	JAK2	missense_variant	234	R/C	NM_004972.3:c.700C>T	NM_004972.3	c.700C>T	NP_004963.1	NP_004963.1:p.Arg234Cys
GLDC-9-6595126-G>G/C	GLDC	intron_variant	0	NA	NM_000170.2:c.1156-7C>G	NM_000170.2	c.1156-7C>G	NP_000161.2	NA
IFNA10-9-21206743-G>G/T	IFNA10	missense_variant	118	D/E	NM_002171.1:c.354C>A	NM_002171.1	c.354C>A	NP_002162.1	NP_002162.1:p.Asp118Glu
AQP7-9-33386144-G>G/A	AQP7	synonymous_variant	152	V	NM_001170.1:c.456C>T	NM_001170.1	c.456C>T	NP_001161.1	NM_001170.1:c.456C>T(p.=)
AQP7-9-33386146-C>C/A	AQP7	missense_variant	152	V/F	NM_001170.1:c.454G>T	NM_001170.1	c.454G>T	NP_001161.1	NP_001161.1:p.Val152Phe
AQP7-9-33386167-G>G/C	AQP7	missense_variant	145	Q/E	NM_001170.1:c.433C>G	NM_001170.1	c.433C>G	NP_001161.1	NP_001161.1:p.Gln145Glu
AQP7-9-33386465-A>A/G	AQP7	missense_variant	115	Y/H	NM_001170.1:c.343T>C	NM_001170.1	c.343T>C	NP_001161.1	NP_001161.1:p.Tyr115His
AQP7-9-33386469-C>C/T	AQP7	synonymous_variant	113	P	NM_001170.1:c.339G>A	NM_001170.1	c.339G>A	NP_001161.1	NM_001170.1:c.339G>A(p.=)
AQP7-9-33386510-C>C/T	AQP7	missense_variant	100	A/T	NM_001170.1:c.298G>A	NM_001170.1	c.298G>A	NP_001161.1	NP_001161.1:p.Ala100Thr
AQP7-9-33386511-A>A/G	AQP7	synonymous_variant	99	F	NM_001170.1:c.297T>C	NM_001170.1	c.297T>C	NP_001161.1	NM_001170.1:c.297T>C(p.=)
AQP7-9-33386526-G>G/A	AQP7	synonymous_variant	94	N	NM_001170.1:c.282C>T	NM_001170.1	c.282C>T	NP_001161.1	NM_001170.1:c.282C>T(p.=)
TPM2-9-35683240-T>T/TG	TPM2	intron_variant	0	NA	NM_213674.1:c.772+1002dupC	NM_213674.1	c.772+1002dupC	NP_998839.1	NA
VPS13A-9-79867181-G>G/A	VPS13A	missense_variant	734	S/N	NM_033305.2:c.2201G>A	NM_033305.2	c.2201G>A	NP_150648.2	NP_150648.2:p.Ser734Asn
DAPK1-9-90322189-C>C/T	DAPK1	synonymous_variant	1401	I	NM_004938.2:c.4203C>T	NM_004938.2	c.4203C>T	NP_004929.2	NM_004938.2:c.4203C>T(p.=)
NINJ1-9-95888872-C>C/T	NINJ1	missense_variant	42	A/T	NM_004148.3:c.124G>A	NM_004148.3	c.124G>A	NP_004139.2	NP_004139.2:p.Ala42Thr
ALG2-9-101978997-C>C/T	ALG2	3_prime_UTR_variant	0	NA	NM_033087.3:c.*1219G>A	NM_033087.3	c.*1219G>A	NP_149078.1	NA
INVS-9-103060213-T>T/G	INVS	intron_variant	0	NA	NM_014425.3:c.3017-5T>G	NM_014425.3	c.3017-5T>G	NP_055240.2	NA
GRIN3A-9-104499916-C>C/T	GRIN3A	missense_variant	116	G/S	NM_133445.2:c.346G>A	NM_133445.2	c.346G>A	NP_597702.2	NP_597702.2:p.Gly116Ser
TSC1-9-135781132-T>T/C	TSC1	synonymous_variant	611	A	NM_000368.4:c.1833A>G	NM_000368.4	c.1833A>G	NP_000359.1	NM_000368.4:c.1833A>G(p.=)
CEL-9-135940587-C>C/T	CEL	synonymous_variant	170	P	NM_001807.3:c.510C>T	NM_001807.3	c.510C>T	NP_001798.2	NM_001807.3:c.510C>T(p.=)
CEL-9-135940588-C>C/T	CEL	missense_variant	171	L/F	NM_001807.3:c.511C>T	NM_001807.3	c.511C>T	NP_001798.2	NP_001798.2:p.Leu171Phe
CEL-9-135944524-C>C/T	CEL	synonymous_variant	391	T	NM_001807.3:c.1173C>T	NM_001807.3	c.1173C>T	NP_001798.2	NM_001807.3:c.1173C>T(p.=)

RALGDS-9-135974040-G>G/A	RALGDS	synonymous_variant	893	H	NM_006266.2:c.2679C>T	NM_006266.2	c.2679C>T	NP_006257.1	NM_006266.2:c.2679C>T(p.=)
GBGT1-9-136036927-TA>TA/T	GBGT1	intron_variant	0	NA	NM_021996.4:c.72-9delT	NM_021996.4	c.72-9delT	NP_068836.2	NA
ADAMTSL2-9-136434598-A>G/G	ADAMTSL 2	synonymous_variant	771	V	NM_001145320.1:c.2313A>G	NM_001145320.1	c.2313A>G	NP_001138792.1	NM_001145320.1:c.2313A>G(p.=)
ADAMTSL2-9-136435473-C>T/T	ADAMTSL 2	synonymous_variant	812	R	NM_001145320.1:c.2436C>T	NM_001145320.1	c.2436C>T	NP_001138792.1	NM_001145320.1:c.2436C>T(p.=)
MAN1B1-9-140000648-C>C/T	MAN1B1	synonymous_variant	442	F	NM_016219.4:c.1326C>T	NM_016219.4	c.1326C>T	NP_057303.2	NM_016219.4:c.1326C>T(p.=)
TPRN-9-140093903-G>G/A	TPRN	missense_variant	421	P/S	NM_001128228.2:c.1261C>T	NM_001128228.2	c.1261C>T	NP_001121700.2	NP_001121700.2:p.Pro421Ser
DCLRE1C-10-14968831-G>G/A	DCLRE1C	intron_variant	0	NA	NM_001033855.1:c.972+11C>T	NM_001033855.1	c.972+11C>T	NP_001029027.1	NA
GAD2-10-26569981-C>C/T	GAD2	missense_variant	401	P/S	NM_000818.2:c.1201C>T	NM_000818.2	c.1201C>T	NP_000809.1	NP_000809.1:p.Pro401Ser
ZEB1-10-31809400-C>C/T	ZEB1	synonymous_variant	380	G	NM_001174096.1:c.1140C>T	NM_001174096.1	c.1140C>T	NP_001167567.1	NM_001174096.1:c.1140C>T(p.=)
PCDH15-10-55600246-G>G/A	PCDH15	missense_variant	1278	R/C	NM_001142763.1:c.3832C>T	NM_001142763.1	c.3832C>T	NP_001136235.1	NP_001136235.1:p.Arg1278Cys
MYPN-10-69905257-C>C/T	MYPN	synonymous_variant	368	G	NM_001256267.1:c.1104C>T	NM_001256267.1	c.1104C>T	NP_001243196.1	NM_001256267.1:c.1104C>T(p.=)
KAT6B-10-76781905-GGAA>GGAA/G	KAT6B	inframe	1097	E/-	NM_012330.3:c.3289_3291delGAA	NM_012330.3	c.3289_3291delGAA	NP_036462.2	NP_036462.2:p.Glu1097del
DLG5-10-79576332-G>G/A	DLG5	synonymous_variant	1334	S	NM_004747.3:c.4002C>T	NM_004747.3	c.4002C>T	NP_004738.3	NM_004747.3:c.4002C>T(p.=)
ANXA11-10-81925960-G>G/C	ANXA11	intron_variant	0	NA	NM_001157.2:c.745-7C>G	NM_001157.2	c.745-7C>G	NP_001148.1	NA
ANXA11-10-81926659-G>G/A	ANXA11	missense_variant	244	T/M	NM_001157.2:c.731C>T	NM_001157.2	c.731C>T	NP_001148.1	NP_001148.1:p.Thr244Met
PAPSS2-10-89469057-C>C/G	PAPSS2	synonymous_variant	44	T	NM_001015880.1:c.132C>G	NM_001015880.1	c.132C>G	NP_001015880.1	NM_001015880.1:c.132C>G(p.=)
ANKRD1-10-92678740-AATAAATAAATATATATAT>AATAAATAAATATATATAT/A	ANKRD1	intron_variant	0	NA	NM_014391.2:c.346-29_346-12delATATATATATTTATTTAT	NM_014391.2	c.346-29_346-12delATATATATATTTATTTAT	NP_055206.2	NA
FFAR4-10-95347307-G>G/A	FFAR4	missense_variant	359	G/R	NM_181745.3:c.1075G>A	NM_181745.3	c.1075G>A	NP_859529.2	NP_859529.2:p.Gly359Arg
PLCE1-10-95791566-C>C/T	PLCE1	missense_variant	255	H/Y	NM_016341.3:c.763C>T	NM_016341.3	c.763C>T	NP_057425.3	NP_057425.3:p.His255Tyr
CYP2C9-10-96741038-G>G/A	CYP2C9	missense_variant	354	E/K	NM_000771.3:c.1060G>A	NM_000771.3	c.1060G>A	NP_000762.2	NP_000762.2:p.Glu354Lys
CYP2C8-10-96824553-T>T/G	CYP2C8	intron_variant	0	NA	NM_000770.3:c.642+4A>C	NM_000770.3	c.642+4A>C	NP_000761.3	NA
GOT1-10-101180557-G>G/A	GOT1	missense_variant	42	R/C	NM_002079.2:c.124C>T	NM_002079.2	c.124C>T	NP_002070.1	NP_002070.1:p.Arg42Cys
WDR11-10-122664835-C>C/T	WDR11	synonymous_variant	1066	G	NM_018117.11:c.3198C>T	NM_018117.11	c.3198C>T	NP_060587.8	NM_018117.11:c.3198C>T(p.=)
MGMT-10-131557574-G>G/A	MGMT	missense_variant	159	R/Q	NM_002412.3:c.476G>A	NM_002412.3	c.476G>A	NP_002403.2	NP_002403.2:p.Arg159Gln
MUC5B-11-1253942-T>T/C	MUC5B	synonymous_variant	669	Y	NM_002458.2:c.2007T>C	NM_002458.2	c.2007T>C	NP_002449.2	NM_002458.2:c.2007T>C(p.=)
SMPD1-11-6411935-TGCTGGC>T/T	SMPD1	inframe	NA	VLA/V	NM_000543.4:c.108_113delGCTGGC	NM_000543.4	c.108_113delGCTGGC	NP_000534.3	NP_000534.3:p.Leu37_Ala38del
SAA2-SAA4.SAA2-11-18267463-A>A/G	SAA2-SAA4.SAA 2	missense_variant	75	V/A	NM_030754.4:c.224T>C	NM_030754.4	c.224T>C	NP_110381.2	NP_110381.2:p.Val75Ala
SAA2-SAA4.SAA2-11-18267478-G>G/A	SAA2-SAA4.SAA 2	missense_variant	70	A/V	NM_030754.4:c.209C>T	NM_030754.4	c.209C>T	NP_110381.2	NP_110381.2:p.Ala70Val
WT1-AS-11-32461025-A>A/AGG	WT1-AS	nc_exon_variant	0	NA	NR_023920.1:n.1743_1744insGG	NR_023920.1	n.1743_1744insGG	NA	NA
MYBPC3-11-47372812-G>G/A	MYBPC3	synonymous_variant	90	F	NM_000256.3:c.270C>T	NM_000256.3	c.270C>T	NP_000247.2	NM_000256.3:c.270C>T(p.=)
TMEM216-11-61165741-G>C/C	TMEM216	missense_variant	147	R/T	NM_001173991.2:c.440G>C	NM_001173991.2	c.440G>C	NP_001167462.1	NP_001167462.1:p.Arg147Thr
GAL3ST3-11-65810470-G>G/A	GAL3ST3	synonymous_variant	268	N	NM_033036.2:c.804C>T	NM_033036.2	c.804C>T	NP_149025.1	NM_033036.2:c.804C>T(p.=)

UNC93B1-11-67765163-A>AG/AG	UNC93B1	frameshift_variant	296	NA	NM_030930.2:c.887dupC	NM_030930.2	c.887dupC	NP_112192.2	NP_112192.2:p.Ser297PhefsTer302
SERPINH1-11-75277974-C>C/A	SERPINH1	missense_variant	194	R/S	NM_001235.3:c.580C>A	NM_001235.3	c.580C>A	NP_001226.2	NP_001226.2:p.Arg194Ser
MMP12-11-102738793-G>GT/GT	MMP12	frameshift_variant	211	NA	NM_002426.4:c.631dupA	NM_002426.4	c.631dupA	NP_002417.2	NP_002417.2:p.Thr211AsnfsTer261
MMP12-11-102738795-G>T/T	MMP12	synonymous_variant	210	T	NM_002426.4:c.630C>A	NM_002426.4	c.630C>A	NP_002417.2	NM_002426.4:c.630C>A(p.=)
ATM-11-108119712-C>C/A	ATM	missense_variant	373	T/K	NM_000051.3:c.1118C>A	NM_000051.3	c.1118C>A	NP_000042.3	NP_000042.3:p.Thr373Lys
ANKK1-11-113269718-C>C/T	ANKK1	missense_variant	343	L/F	NM_178510.1:c.1027C>T	NM_178510.1	c.1027C>T	NP_848605.1	NP_848605.1:p.Leu343Phe
CADM1-11-115085329-G>G/A	CADM1	synonymous_variant	331	Y	NM_014333.3:c.993C>T	NM_014333.3	c.993C>T	NP_055148.3	NM_014333.3:c.993C>T(p.=)
CBL-11-119149312-C>C/T	CBL	synonymous_variant	440	G	NM_005188.3:c.1320C>T	NM_005188.3	c.1320C>T	NP_005179.2	NM_005188.3:c.1320C>T(p.=)
WNK1-12-988802-C>C/T	WNK1	intron_variant	0	NA	NM_001184985.1:c.3613-1085C>T	NM_001184985.1	c.3613-1085C>T	NP_001171914.1	NA
WNK1-12-988803-C>C/G	WNK1	intron_variant	0	NA	NM_001184985.1:c.3613-1084C>G	NM_001184985.1	c.3613-1084C>G	NP_001171914.1	NA
CACNA2D4-12-1919467-G>G/A	CACNA2D4	synonymous_variant	900	L	NM_172364.4:c.2698C>T	NM_172364.4	c.2698C>T	NP_758952.4	NM_172364.4:c.2698C>T(p.=)
CACNA1C-12-2774062-C>C/T	CACNA1C	missense_variant	1483	S/F	NM_199460.2:c.4448C>T	NM_199460.2	c.4448C>T	NP_955630.2	NP_955630.2:p.Ser1483Phe
EMG1-12-7080210-A>AG/AG	EMG1	frameshift_variant	42	NA	NM_006331.7:c.124_125insG	NM_006331.7	c.124_125insG	NP_006322.4	NP_006322.4:p.Ser42ArgfsTer204
EMG1-12-7080212-T>C/C	EMG1	synonymous_variant	42	S	NM_006331.7:c.126T>C	NM_006331.7	c.126T>C	NP_006322.4	NM_006331.7:c.126T>C(p.=)
PRB3-12-11420333-AG>A/A	PRB3	frameshift_variant	241	NA	NM_006249.4:c.723delC	NM_006249.4	c.723delC	NP_006240.4	NP_006240.4:p.Ser242HisfsTer69
GRIN2B-12-13716542-G>G/A	GRIN2B	synonymous_variant	1210	S	NM_000834.3:c.3630C>T	NM_000834.3	c.3630C>T	NP_000825.2	NM_000834.3:c.3630C>T(p.=)
PIK3C2G-12-18644400-G>G/A	PIK3C2G	missense_variant	860	E/K	NM_004570.4:c.2578G>A	NM_004570.4	c.2578G>A	NP_004561.3	NP_004561.3:p.Glu860Lys
SLCO1B3-12-21028298-C>C/T	SLCO1B3	missense_variant	286	P/L	NM_019844.3:c.857C>T	NM_019844.3	c.857C>T	NP_062818.1	NP_062818.1:p.Pro286Leu
DDX11-12-31242358-G>G/A	DDX11	missense_variant	272	V/M	NM_001257144.1:c.814G>A	NM_001257144.1	c.814G>A	NP_001244073.1	NP_001244073.1:p.Val272Met
DDX11-12-31247682-T>T/C	DDX11	intron_variant	0	NA	NM_001257144.1:c.1415-7T>C	NM_001257144.1	c.1415-7T>C	NP_001244073.1	NA
PKP2-12-32955454-C>C/T	PKP2	missense_variant	728	E/K	NM_004572.3:c.2182G>A	NM_004572.3	c.2182G>A	NP_004563.2	NP_004563.2:p.Glu728Lys
ANO6-12-45822896-C>C/T	ANO6	synonymous_variant	866	I	NM_001204803.1:c.2598C>T	NM_001204803.1	c.2598C>T	NP_001191732.1	NM_001204803.1:c.2598C>T(p.=)
KRT1-12-53074020-C>C/T	KRT1	missense_variant	38	R/H	NM_006121.3:c.113G>A	NM_006121.3	c.113G>A	NP_006112.3	NP_006112.3:p.Arg38His
AMHR2-12-53819230-C>C/T	AMHR2	intron_variant	0	NA	NM_020547.2:c.503-9C>T	NM_020547.2	c.503-9C>T	NP_065434.1	NA
SMUG1-12-54577582-G>G/A	SMUG1	missense_variant	48	S/L	NM_001243787.1:c.143C>T	NM_001243787.1	c.143C>T	NP_001230716.1	NP_001230716.1:p.Ser48Leu
STAT6-12-57499387-G>G/A	STAT6	intron_variant	0	NA	NM_003153.4:c.681-5C>T	NM_003153.4	c.681-5C>T	NP_003144.3	NA
STAT6-12-57499388-G>G/A	STAT6	intron_variant	0	NA	NM_003153.4:c.681-6C>T	NM_003153.4	c.681-6C>T	NP_003144.3	NA
PAWR-12-80014907-A>T/T	PAWR	synonymous_variant	199	I	NM_002583.2:c.597T>A	NM_002583.2	c.597T>A	NP_002574.2	NM_002583.2:c.597T>A(p.=)
PTPRQ-12-81014013-C>C/T	PTPRQ	synonymous_variant	1672	F	NM_001145026.1:c.5016C>T	NM_001145026.1	c.5016C>T	NP_001138498.1	NM_001145026.1:c.5016C>T(p.=)
CEP290-12-88483184-A>A/G	CEP290	synonymous_variant	1218	L	NM_025114.3:c.3654T>C	NM_025114.3	c.3654T>C	NP_079390.3	NM_025114.3:c.3654T>C(p.=)
ACACB-12-109631451-A>A/C	ACACB	splice_variant	0	NA	NM_001093.3:c.2400-2A>C	NM_001093.3	c.2400-2A>C	NP_001084.3	NA
ACACB-12-109703311-GTCC>GTCC/G	ACACB	inframe	NA	VL/V	NM_001093.3:c.7232_7234delTCC	NM_001093.3	c.7232_7234delTCC	NP_001084.3	NP_001084.3:p.Leu2412del
MMAB-12-109992958-T>T/TTA	MMAB	3_prime_UTR_variant	0	NA	NM_052845.3:c.*1873_*1874dupTA	NM_052845.3	c.*1873_*1874dupTA	NP_443077.1	NA
SETD8-12-123892095-T>T/C	SETD8	missense_variant	302	C/R	NM_020382.3:c.904T>C	NM_020382.3	c.904T>C	NP_065115.3	NP_065115.3:p.Cys302Arg
SETD8-12-123892235-G>G/A	SETD8	synonymous_variant	348	P	NM_020382.3:c.1044G>A	NM_020382.3	c.1044G>A	NP_065115.3	NM_020382.3:c.1044G>A(p.=)
SCARB1-12-125299559-G>G/A	SCARB1	missense_variant	129	S/L	NM_005505.4:c.386C>T	NM_005505.4	c.386C>T	NP_005496.4	NP_005496.4:p.Ser129Leu

GALNT9-12-132683770-C>C/T	GALNT9	synonymous_variant	NA	A		NM_021808		NA	NA
GOLGA3-12-133350849-G>G/A	GOLGA3	missense_variant	1401	P/S	NM_005895.3:c.4201C>T	NM_005895.3	c.4201C>T	NP_005886.2	NP_005886.2:p.Pro1401Ser
ALOX5AP-13-31287979-G>GTA/GTA	ALOX5AP	splice_variant	0	NA	NM_001204406.1:c.116+1_116+2insTA	NM_001204406.1	c.116+1_116+2insTA	NP_001191335.1	NA
B3GALTL-13-31821152-T>T/C	B3GALTL	intron_variant	0	NA	NM_194318.3:c.271-8T>C	NM_194318.3	c.271-8T>C	NP_919299.3	NA
B3GALTL-13-31903679-A>A/G	B3GALTL	synonymous_variant	457	Q	NM_194318.3:c.1371A>G	NM_194318.3	c.1371A>G	NP_919299.3	NM_194318.3:c.1371A>G(p.=)
RXFP2-13-32356863-C>C/T	RXFP2	missense_variant	303	S/L	NM_130806.3:c.908C>T	NM_130806.3	c.908C>T	NP_570718.1	NP_570718.1:p.Ser303Leu
COG6-13-40229756-C>C/T	COG6	up-down_stream_gene_variant	0	NA		NM_020751.2		NP_065802.1	NA
CPB2-AS1.CPB2-13-46658424-G>G/A	CPB2-AS1.CPB2	intron_variant	0	NA	NR_046226.1:n.119-10564G>A	NR_046226.1	n.119-10564G>A	NA	NA
DIAPH3-13-60385060-A>ATTAC/ATTAC	DIAPH3	intron_variant	0	NA	NM_001042517.1:c.3028-4_3028-3insGTAA	NM_001042517.1	c.3028-4_3028-3insGTAA	NP_001035982.1	NA
DOCK9-13-99512723-C>C/G	DOCK9	synonymous_variant	1212	P	NM_015296.2:c.3636G>C	NM_015296.2	c.3636G>C	NP_056111.1	NM_015296.2:c.3636G>C(p.=)
PROZ-13-113818817-T>TC/TC	PROZ	intron_variant	0	NA	NM_001256134.1:c.440-10_440-9insC	NM_001256134.1	c.440-10_440-9insC	NP_001243063.1	NA
RPGRIP1-14-21796751-C>C/T	RPGRIP1	missense_variant	1022	L/F	NM_020366.3:c.3064C>T	NM_020366.3	c.3064C>T	NP_065099.3	NP_065099.3:p.Leu1022Phe
MYH7-14-23888685-G>G/A	MYH7	intron_variant	0	NA	NM_000257.2:c.3853+7C>T	NM_000257.2	c.3853+7C>T	NP_000248.2	NA
CFL2-14-35179728-C>C/T	CFL2	3_prime_UTR_variant	0	NA	NM_021914.7:c.*2343G>A	NM_021914.7	c.*2343G>A	NP_068733.1	NA
SEC23A-14-39502539-G>GA/GA	SEC23A	intron_variant	0	NA	NM_006364.2:c.2209-8dupT	NM_006364.2	c.2209-8dupT	NP_006355.2	NA
PTGER2-14-52782001-C>C/A	PTGER2	synonymous_variant	245	P	NM_000956.3:c.735C>A	NM_000956.3	c.735C>A	NP_000947.2	NM_000956.3:c.735C>A(p.=)
OTX2-14-57268545-T>T/G	OTX2	missense_variant	268	K/Q	NM_001270525.1:c.802A>C	NM_001270525.1	c.802A>C	NP_001257454.1	NP_001257454.1:p.Lys268Gln
HIF1A-14-62207553-G>G/A	HIF1A	synonymous_variant	604	L	NM_001243084.1:c.1812G>A	NM_001243084.1	c.1812G>A	NP_001230013.1	NM_001243084.1:c.1812G>A(p.=)
CCDC88C-14-91755563-C>C/T	CCDC88C	missense_variant	1443	A/T	NM_001080414.3:c.4327G>A	NM_001080414.3	c.4327G>A	NP_001073883.2	NP_001073883.2:p.Ala1443Thr
JAG2-14-105609822-T>T/C	JAG2	missense_variant	1080	T/A	NM_002226.4:c.3238A>G	NM_002226.4	c.3238A>G	NP_002217.3	NP_002217.3:p.Thr1080Ala
MAGEL2-15-23889763-G>G/C	MAGEL2	missense_variant	1043	R/G	NM_019066.4:c.3127C>G	NM_019066.4	c.3127C>G	NP_061939.3	NP_061939.3:p.Arg1043Gly
MAGEL2-15-23890004-G>G/A	MAGEL2	synonymous_variant	962	S	NM_019066.4:c.2886C>T	NM_019066.4	c.2886C>T	NP_061939.3	NM_019066.4:c.2886C>T(p.=)
PWRN1-15-24832739-C>C/T	PWRN1	nc_exon_variant	0	NA	NR_026646.1:n.1251C>T	NR_026646.1	n.1251C>T	NA	NA
UBE3A-15-25616703-T>T/A	UBE3A	synonymous_variant	209	A	NM_000462.3:c.627A>T	NM_000462.3	c.627A>T	NP_000453.2	NM_000462.3:c.627A>T(p.=)
ATP10A-15-26108141-G>G/C	ATP10A	missense_variant	35	P/A	NM_024490.3:c.103C>G	NM_024490.3	c.103C>G	NP_077816.1	NP_077816.1:p.Pro35Ala
HERC2-15-28408400-G>G/A	HERC2	missense_variant	3529	P/L	NM_004667.5:c.10586C>T	NM_004667.5	c.10586C>T	NP_004658.3	NP_004658.3:p.Pro3529Leu
HERC2-15-28408401-G>G/A	HERC2	missense_variant	3529	P/S	NM_004667.5:c.10585C>T	NM_004667.5	c.10585C>T	NP_004658.3	NP_004658.3:p.Pro3529Ser
HERC2-15-28467246-T>C/C	HERC2	synonymous_variant	1860	G	NM_004667.5:c.5580A>G	NM_004667.5	c.5580A>G	NP_004658.3	NM_004667.5:c.5580A>G(p.=)
HERC2-15-28517436-T>T/C	HERC2	synonymous_variant	336	P	NM_004667.5:c.1008A>G	NM_004667.5	c.1008A>G	NP_004658.3	NM_004667.5:c.1008A>G(p.=)
SPTBN5-15-42143145-C>C/CCA	SPTBN5	intron_variant	0	NA	NM_016642.2:c.10731-10_10731-9dupTG	NM_016642.2	c.10731-10_10731-9dupTG	NP_057726.3	NA
UBR1-15-43313515-G>G/A	UBR1	synonymous_variant	966	P	NM_174916.2:c.2898C>T	NM_174916.2	c.2898C>T	NP_777576.1	NM_174916.2:c.2898C>T(p.=)
GATM-15-45661653-G>G/A	GATM	missense_variant	119	H/Y	NM_001482.2:c.355C>T	NM_001482.2	c.355C>T	NP_001473.1	NP_001473.1:p.His119Tyr
HDC-15-50545834-G>G/A	HDC	synonymous_variant	250	V	NM_002112.3:c.750C>T	NM_002112.3	c.750C>T	NP_002103.2	NM_002112.3:c.750C>T(p.=)
MTFMT-15-65321780-A>A/T	MTFMT	missense_variant	58	F/I	NM_139242.3:c.172T>A	NM_139242.3	c.172T>A	NP_640335.2	NP_640335.2:p.Phe58Ile
PML-15-74317251-C>C/T	PML	missense_variant	413	P/S	NM_033238.2:c.1237C>T	NM_033238.2	c.1237C>T	NP_150241.2	NP_150241.2:p.Pro413Ser
NEIL1-15-75644482-G>G/A	NEIL1	synonymous_variant	241	K	NM_001256552.1:c.723G>A	NM_001256552.1	c.723G>A	NP_001243481.1	NM_001256552.1:c.723G>A(p.=)

PSTPIP1-15-77329409-C>C/T	PSTPIP1	synonymous_variant	381	S	NM_003978.3:c.1143C>T	NM_003978.3	c.1143C>T	NP_003969.2	NM_003978.3:c.1143C>T(p.=)
CHRNA3-15-78913067-ACAG>A/A	CHRNA3	inframe	23	L/-	NM_000743.4:c.67_69delCTG	NM_000743.4	c.67_69delCTG	NP_000734.2	NP_000734.2:p.Leu23del
AKAP13-15-86265465-A>A/T	AKAP13	missense_variant	2132	K/M	NM_006738.4:c.6395A>T	NM_006738.4	c.6395A>T	NP_006729.4	NP_006729.4:p.Lys2132Met
ACAN-15-89382261-G>G/A	ACAN	synonymous_variant	146	L	NM_013227.3:c.438G>A	NM_013227.3	c.438G>A	NP_037359.3	NM_013227.3:c.438G>A(p.=)
KIF7-15-90173734-C>CAG/CAG	KIF7	intron_variant	0	NA	NM_198525.2:c.3112-11_3112-10insCT	NM_198525.2	c.3112-11_3112-10insCT	NP_940927.2	NA
IQGAP1-15-91020976-GC>GC/G	IQGAP1	frameshift_variant	1062	NA	NM_003870.3:c.3185delC	NM_003870.3	c.3185delC	NP_003861.1	NP_003861.1:p.Arg1063ValfsTer6
IQGAP1-15-91020978-C>C/A	IQGAP1	synonymous_variant	1062	A	NM_003870.3:c.3186C>A	NM_003870.3	c.3186C>A	NP_003861.1	NM_003870.3:c.3186C>A(p.=)
LMF1-16-1031305-A>A/T	LMF1	up-down_stream_gene_variant	0	NA		NM_014587.3		NP_055402.2	NA
CACNA1H-16-1256259-C>C/T	CACNA1H	missense_variant	920	T/M	NM_021098.2:c.2759C>T	NM_021098.2	c.2759C>T	NP_066921.2	NP_066921.2:p.Thr920Met
TPSB2-16-1278767-C>C/T	TPSB2	missense_variant	238	A/T	NM_024164.5:c.712G>A	NM_024164.5	c.712G>A	NP_077078.5	NP_077078.5:p.Ala238Thr
TPSB2-16-1279732-C>C/A	TPSB2	missense_variant	23	G/V	NM_024164.5:c.68G>T	NM_024164.5	c.68G>T	NP_077078.5	NP_077078.5:p.Gly23Val
TSC2-16-2134508-G>G/T	TSC2	missense_variant	1429	A/S	NM_000548.3:c.4285G>T	NM_000548.3	c.4285G>T	NP_000539.2	NP_000539.2:p.Ala1429Ser
PKD1-16-2147471-G>G/A	PKD1	synonymous_variant	3418	L	NM_001009944.2:c.10254C>T	NM_001009944.2	c.10254C>T	NP_001009944.2	NM_001009944.2:c.10254C>T(p.=)
PKD1-16-2159255-C>C/T	PKD1	synonymous_variant	1971	V	NM_001009944.2:c.5913G>A	NM_001009944.2	c.5913G>A	NP_001009944.2	NM_001009944.2:c.5913G>A(p.=)
PKD1-16-2165395-G>G/A	PKD1	missense_variant	694	P/L	NM_001009944.2:c.2081C>T	NM_001009944.2	c.2081C>T	NP_001009944.2	NP_001009944.2:p.Pro694Leu
PKD1-16-2167874-G>A/A	PKD1	synonymous_variant	373	L	NM_001009944.2:c.1119C>T	NM_001009944.2	c.1119C>T	NP_001009944.2	NM_001009944.2:c.1119C>T(p.=)
PKD1-16-2167970-G>A/A	PKD1	synonymous_variant	341	A	NM_001009944.2:c.1023C>T	NM_001009944.2	c.1023C>T	NP_001009944.2	NM_001009944.2:c.1023C>T(p.=)
PDPK1-16-2647690-C>C/T	PDPK1	synonymous_variant	531	N	NM_002613.4:c.1593C>T	NM_002613.4	c.1593C>T	NP_002604.1	NM_002613.4:c.1593C>T(p.=)
CREBBP-16-3820811-G>G/A	CREBBP	synonymous_variant	880	P	NM_004380.2:c.2640C>T	NM_004380.2	c.2640C>T	NP_004371.2	NM_004380.2:c.2640C>T(p.=)
CREBBP-16-3820812-G>G/A	CREBBP	missense_variant	880	P/L	NM_004380.2:c.2639C>T	NM_004380.2	c.2639C>T	NP_004371.2	NP_004371.2:p.Pro880Leu
USP7-16-9017061-T>TACAACGATGTGGGGGTTTGTAGTTGCATCATTTAA/TACAACGATGTGGGGGTTTGTAGTTGCATCATTTAA	USP7	intron_variant	0	NA	NM_003470.2:c.383+10_383+11insTTAAATGATGCAACTACAAACCCCCACATCGTTGT	NM_003470.2	c.383+10_383+11insTTAAATGATGCAACTACAAACCCCCACATCGTTGT	NP_003461.2	NA
GRIN2A-16-9857672-C>C/T	GRIN2A	synonymous_variant	1243	G	NM_001134407.1:c.3729G>A	NM_001134407.1	c.3729G>A	NP_001127879.1	NM_001134407.1:c.3729G>A(p.=)
GRIN2A-16-9858517-C>C/T	GRIN2A	missense_variant	962	E/K	NM_001134407.1:c.2884G>A	NM_001134407.1	c.2884G>A	NP_001127879.1	NP_001127879.1:p.Glu962Lys
LITAF-16-11643394-G>G/A	LITAF	3_prime_UTR_variant	0	NA	NM_001136472.1:c.*99C>T	NM_001136472.1	c.*99C>T	NP_001129944.1	NA
SMG1-16-18900862-A>G/G	SMG1	synonymous_variant	218	L	NM_015092.4:c.654T>C	NM_015092.4	c.654T>C	NP_055907.3	NM_015092.4:c.654T>C(p.=)
ACSM3.ERI2-16-20792417-C>C/T	ACSM3.ERI2	missense_variant	302	H/Y	NM_005622.3:c.904C>T	NM_005622.3	c.904C>T	NP_005613.2	NP_005613.2:p.His302Tyr
EEF2K-16-22277743-C>C/T	EEF2K	synonymous_variant	491	S	NM_013302.3:c.1473C>T	NM_013302.3	c.1473C>T	NP_037434.1	NM_013302.3:c.1473C>T(p.=)
TOX3-16-52473331-G>G/A	TOX3	missense_variant	513	R/C	NM_001080430.2:c.1537C>T	NM_001080430.2	c.1537C>T	NP_001073899.2	NP_001073899.2:p.Arg513Cys
TOX3-16-52484384-G>G/A	TOX3	synonymous_variant	161	T	NM_001080430.2:c.483C>T	NM_001080430.2	c.483C>T	NP_001073899.2	NM_001080430.2:c.483C>T(p.=)
CES1-16-55844928-A>A/C	CES1	intron_variant	0	NA	NM_001025195.1:c.1087-6T>G	NM_001025195.1	c.1087-6T>G	NP_001020366.1	NA
CCL22-16-57394393-T>T/C	CCL22	missense_variant	40	Y/H	NM_002990.4:c.118T>C	NM_002990.4	c.118T>C	NP_002981.2	NP_002981.2:p.Tyr40His

CNGB1-16-57951298-G>G/A	CNGB1	synonymous_variant	680	F	NM_001297.4:c.2040C>T	NM_001297.4	c.2040C>T	NP_001288.3	NM_001297.4:c.2040C>T(p.=)
CDH8-16-62055181-G>G/A	CDH8	missense_variant	43	P/S	NM_001796.4:c.127C>T	NM_001796.4	c.127C>T	NP_001787.2	NP_001787.2:p.Pro43Ser
CDH5-16-66432424-C>T/T	CDH5	synonymous_variant	517	I	NM_001795.3:c.1551C>T	NM_001795.3	c.1551C>T	NP_001786.2	NM_001795.3:c.1551C>T(p.=)
PLEKHG4-16-67314605-C>C/T	PLEKHG4	intron_variant	0	NA	NM_015432.3:c.500-9C>T	NM_015432.3	c.500-9C>T	NP_056247.1	NA
COG8-16-69368806-G>G/A	COG8	missense_variant	344	S/F	NM_032382.4:c.1031C>T	NM_032382.4	c.1031C>T	NP_115758.3	NP_115758.3:p.Ser344Phe
AARS-16-70298925-C>C/G	AARS	synonymous_variant	476	R	NM_001605.2:c.1428G>C	NM_001605.2	c.1428G>C	NP_001596.2	NM_001605.2:c.1428G>C(p.=)
COG4-16-70557400-C>C/G	COG4	missense_variant	16	G/A	NM_015386.2:c.47G>C	NM_015386.2	c.47G>C	NP_056201.2	NP_056201.2:p.Gly16Ala
HYDIN-16-70884524-C>C/G	HYDIN	missense_variant	4159	E/Q	NM_032821.2:c.12475G>C	NM_032821.2	c.12475G>C	NP_116210.2	NP_116210.2:p.Glu4159Gln
HYDIN-16-70884534-T>T/A	HYDIN	synonymous_variant	4155	T	NM_032821.2:c.12465A>T	NM_032821.2	c.12465A>T	NP_116210.2	NM_032821.2:c.12465A>T(p.=)
HYDIN-16-70896015-GA>GA/G	HYDIN	frameshift_variant	3903	NA	NM_032821.2:c.11709delT	NM_032821.2	c.11709delT	NP_116210.2	NP_116210.2:p.Gln3904ArgfsTer5
HYDIN-16-70897039-C>C/G	HYDIN	missense_variant	3839	V/L	NM_032821.2:c.11515G>C	NM_032821.2	c.11515G>C	NP_116210.2	NP_116210.2:p.Val3839Leu
HYDIN-16-70908736-A>A/G	HYDIN	synonymous_variant	3547	N	NM_032821.2:c.10641T>C	NM_032821.2	c.10641T>C	NP_116210.2	NM_032821.2:c.10641T>C(p.=)
HYDIN-16-70913386-G>G/A	HYDIN	synonymous_variant	3456	T	NM_032821.2:c.10368C>T	NM_032821.2	c.10368C>T	NP_116210.2	NM_032821.2:c.10368C>T(p.=)
HYDIN-16-70913497-G>G/A	HYDIN	intron_variant	0	NA	NM_032821.2:c.10364+11C>T	NM_032821.2	c.10364+11C>T	NP_116210.2	NA
HYDIN-16-70913501-T>T/C	HYDIN	intron_variant	0	NA	NM_032821.2:c.10364+7A>G	NM_032821.2	c.10364+7A>G	NP_116210.2	NA
HYDIN-16-70917855-A>A/G	HYDIN	missense_variant	3315	L/P	NM_032821.2:c.9944T>C	NM_032821.2	c.9944T>C	NP_116210.2	NP_116210.2:p.Leu3315Pro
HYDIN-16-70917931-C>C/G	HYDIN	missense_variant	3290	A/P	NM_032821.2:c.9868G>C	NM_032821.2	c.9868G>C	NP_116210.2	NP_116210.2:p.Ala3290Pro
HYDIN-16-70942688-A>A/C	HYDIN	missense_variant	2693	I/S	NM_032821.2:c.8078T>G	NM_032821.2	c.8078T>G	NP_116210.2	NP_116210.2:p.Ile2693Ser
HYDIN-16-70972620-G>G/C	HYDIN	missense_variant	2297	R/G	NM_032821.2:c.6889C>G	NM_032821.2	c.6889C>G	NP_116210.2	NP_116210.2:p.Arg2297Gly
HYDIN-16-70975667-T>T/C	HYDIN	missense_variant	2241	Q/R	NM_032821.2:c.6722A>G	NM_032821.2	c.6722A>G	NP_116210.2	NP_116210.2:p.Gln2241Arg
HYDIN-16-70989299-C>C/T	HYDIN	missense_variant	2098	V/M	NM_032821.2:c.6292G>A	NM_032821.2	c.6292G>A	NP_116210.2	NP_116210.2:p.Val2098Met
HYDIN-16-70989335-G>G/A	HYDIN	missense_variant	2086	R/C	NM_032821.2:c.6256C>T	NM_032821.2	c.6256C>T	NP_116210.2	NP_116210.2:p.Arg2086Cys
HYDIN-16-70993566-A>G/G	HYDIN	synonymous_variant	2041	H	NM_032821.2:c.6123T>C	NM_032821.2	c.6123T>C	NP_116210.2	NM_032821.2:c.6123T>C(p.=)
HYDIN-16-70998808-G>G/A	HYDIN	intron_variant	0	NA	NM_032821.2:c.5617-9C>T	NM_032821.2	c.5617-9C>T	NP_116210.2	NA
HYDIN-16-71007354-G>G/A	HYDIN	synonymous_variant	1756	R	NM_032821.2:c.5268C>T	NM_032821.2	c.5268C>T	NP_116210.2	NM_032821.2:c.5268C>T(p.=)
HYDIN-16-71008169-T>T/C	HYDIN	synonymous_variant	1647	L	NM_032821.2:c.4941A>G	NM_032821.2	c.4941A>G	NP_116210.2	NM_032821.2:c.4941A>G(p.=)
HYDIN-16-71012855-T>T/C	HYDIN	missense_variant	1533	I/V	NM_032821.2:c.4597A>G	NM_032821.2	c.4597A>G	NP_116210.2	NP_116210.2:p.Ile1533Val
HYDIN-16-71015290-C>C/G	HYDIN	intron_variant	0	NA	NM_032821.2:c.4507+4G>C	NM_032821.2	c.4507+4G>C	NP_116210.2	NA
HYDIN-16-71015482-G>G/A	HYDIN	intron_variant	0	NA	NM_032821.2:c.4330-11C>T	NM_032821.2	c.4330-11C>T	NP_116210.2	NA
HYDIN-16-71026076-C>C/G	HYDIN	missense_variant	1228	V/L	NM_032821.2:c.3682G>C	NM_032821.2	c.3682G>C	NP_116210.2	NP_116210.2:p.Val1228Leu
HYDIN-16-71026098-C>C/T	HYDIN	synonymous_variant	1220	P	NM_032821.2:c.3660G>A	NM_032821.2	c.3660G>A	NP_116210.2	NM_032821.2:c.3660G>A(p.=)
HYDIN-16-71054178-T>T/C	HYDIN	missense_variant	1077	I/V	NM_032821.2:c.3229A>G	NM_032821.2	c.3229A>G	NP_116210.2	NP_116210.2:p.Ile1077Val
HYDIN-16-71061495-A>A/G	HYDIN	intron_variant	0	NA	NM_032821.2:c.3042+10T>C	NM_032821.2	c.3042+10T>C	NP_116210.2	NA
HYDIN-16-71061529-G>G/A	HYDIN	synonymous_variant	1006	L	NM_032821.2:c.3018C>T	NM_032821.2	c.3018C>T	NP_116210.2	NM_032821.2:c.3018C>T(p.=)
HYDIN-16-71098649-T>T/C	HYDIN	missense_variant	724	N/D	NM_032821.2:c.2170A>G	NM_032821.2	c.2170A>G	NP_116210.2	NP_116210.2:p.Asn724Asp
HYDIN-16-71101200-T>T/C	HYDIN	missense_variant	690	T/A	NM_032821.2:c.2068A>G	NM_032821.2	c.2068A>G	NP_116210.2	NP_116210.2:p.Thr690Ala
HYDIN-16-71101270-C>C/T	HYDIN	synonymous_variant	666	V	NM_032821.2:c.1998G>A	NM_032821.2	c.1998G>A	NP_116210.2	NM_032821.2:c.1998G>A(p.=)
HYDIN-16-71103269-T>T/C	HYDIN	synonymous_variant	625	P	NM_032821.2:c.1875A>G	NM_032821.2	c.1875A>G	NP_116210.2	NM_032821.2:c.1875A>G(p.=)
HYDIN-16-71122408-C>C/T	HYDIN	missense_variant	489	G/D	NM_032821.2:c.1466G>A	NM_032821.2	c.1466G>A	NP_116210.2	NP_116210.2:p.Gly489Asp
HYDIN-16-71209556-C>C/T	HYDIN	missense_variant	157	G/E	NM_032821.2:c.470G>A	NM_032821.2	c.470G>A	NP_116210.2	NP_116210.2:p.Gly157Glu
PHLPP2-16-71683759-G>G/A	PHLPP2	synonymous_variant	1002	H	NM_015020.2:c.3006C>T	NM_015020.2	c.3006C>T	NP_055835.2	NM_015020.2:c.3006C>T(p.=)

ZFHX3-16-72923700-C>C/T	ZFHX3	synonymous_variant	1126	K	NM_006885.3:c.3378G>A	NM_006885.3	c.3378G>A	NP_008816.3	NM_006885.3:c.3378G>A(p.=)
PSMD7-16-74339608-GAGGAGAAAA>GAGGAGAAAA/G	PSMD7	inframe	NA	EEKK/E	NM_002811.4:c.953_961delAGGAGAAAA	NM_002811.4	c.953_961delAGGAGAAAA	NP_002802.2	NP_002802.2:p.Glu319_Lys321del
ADAMTS18-16-77398091-C>C/T	ADAMTS18	missense_variant	322	M/I	NM_199355.2:c.966G>A	NM_199355.2	c.966G>A	NP_955387.1	NP_955387.1:p.Met322Ile
MAF-16-79633805-TGCC>TGCC/T	MAF	5_prime_UTR_variant	0	NA	NM_005360.4:c.-9_-7delGGC	NM_005360.4	c.-9_-7delGGC	NP_005351.2	NA
MLYCD-16-83940707-A>A/C	MLYCD	intron_variant	0	NA	NM_012213.2:c.641+3A>C	NM_012213.2	c.641+3A>C	NP_036345.2	NA
GSE1-16-85690000-CGAGCGT>CGAGCGT/C	GSE1	inframe	NA	ER/-	NM_014615.2:c.1042_1047delGAGCGT	NM_014615.2	c.1042_1047delGAGCGT	NP_055430.1	NP_055430.1:p.Glu348_Arg349delinsdel
GSE1-16-85696615-C>C/T	GSE1	synonymous_variant	763	Y	NM_014615.2:c.2289C>T	NM_014615.2	c.2289C>T	NP_055430.1	NM_014615.2:c.2289C>T(p.=)
APRT-16-88877983-G>G/A	APRT	synonymous_variant	54	H	NM_000485.2:c.162C>T	NM_000485.2	c.162C>T	NP_000476.1	NM_000485.2:c.162C>T(p.=)
ACSF3-16-89220973-C>CAAAT/CAAAT	ACSF3	3_prime_UTR_variant	0	NA	NM_001243279.1:c.*358_*359insAAAT	NM_001243279.1	c.*358_*359insAAAT	NP_001230208.1	NA
PRPF8-17-1582082-G>G/A	PRPF8	missense_variant	565	R/W	NM_006445.3:c.1693C>T	NM_006445.3	c.1693C>T	NP_006436.3	NP_006436.3:p.Arg565Trp
SERPINF2-17-1657575-C>C/T	SERPINF2	missense_variant	408	S/F	NM_000934.3:c.1223C>T	NM_000934.3	c.1223C>T	NP_000925.2	NP_000925.2:p.Ser408Phe
GP1BA-17-4837220-T>T/C	GP1BA	missense_variant	441	S/P	NM_000173.5:c.1321T>C	NM_000173.5	c.1321T>C	NP_000164.5	NP_000164.5:p.Ser441Pro
AIPL1-17-6330216-C>C/T	AIPL1	synonymous_variant	209	R	NM_014336.3:c.627G>A	NM_014336.3	c.627G>A	NP_055151.3	NM_014336.3:c.627G>A(p.=)
CTC1-17-8135249-G>G/A	CTC1	missense_variant	786	P/L	NM_025099.5:c.2357C>T	NM_025099.5	c.2357C>T	NP_079375.3	NP_079375.3:p.Pro786Leu
PFAS-17-8158983-C>C/T	PFAS	missense_variant	183	A/V	NM_012393.2:c.548C>T	NM_012393.2	c.548C>T	NP_036525.1	NP_036525.1:p.Ala183Val
DNAH9-17-11648284-C>C/T	DNAH9	synonymous_variant	2094	I	NM_001372.3:c.6282C>T	NM_001372.3	c.6282C>T	NP_001363.2	NM_001372.3:c.6282C>T(p.=)
SREBF1-17-17719517-T>T/C	SREBF1	intron_variant	0	NA	NM_001005291.2:c.2304+4A>G	NM_001005291.2	c.2304+4A>G	NP_001005291.1	NA
LLGL1-17-18141055-C>C/T	LLGL1	synonymous_variant	624	F	NM_004140.3:c.1872C>T	NM_004140.3	c.1872C>T	NP_004131.3	NM_004140.3:c.1872C>T(p.=)
LIG3-17-33325276-C>C/T	LIG3	synonymous_variant	650	I	NM_013975.3:c.1950C>T	NM_013975.3	c.1950C>T	NP_039269.2	NM_013975.3:c.1950C>T(p.=)
TAF15-17-34171583-A>A/G	TAF15	missense_variant	427	Y/C	NM_139215.2:c.1280A>G	NM_139215.2	c.1280A>G	NP_631961.1	NP_631961.1:p.Tyr427Cys
GPR179-17-36485998-A>A/G	GPR179	missense_variant	1152	S/P	NM_001004334.2:c.3454T>C	NM_001004334.2	c.3454T>C	NP_001004334.2	NP_001004334.2:p.Ser1152Pro
ERBB2-17-37865659-C>C/T	ERBB2	synonymous_variant	176	N	NM_004448.2:c.528C>T	NM_004448.2	c.528C>T	NP_004439.2	NM_004448.2:c.528C>T(p.=)
ATP6V0A1-17-40612952-C>C/T	ATP6V0A1	stop_gained	14	Q/*	NM_001130020.1:c.40C>T	NM_001130020.1	c.40C>T	NP_001123492.1	NP_001123492.1:p.Gln14Ter
ABCC3-17-48753045-G>G/A	ABCC3	missense_variant	923	R/Q	NM_003786.3:c.2768G>A	NM_003786.3	c.2768G>A	NP_003777.2	NP_003777.2:p.Arg923Gln
MPO-17-56350226-G>G/A	MPO	missense_variant	559	R/C	NM_000250.1:c.1675C>T	NM_000250.1	c.1675C>T	NP_000241.1	NP_000241.1:p.Arg559Cys
RPS6KB1-17-57992034-G>G/A	RPS6KB1	synonymous_variant	117	G	NM_003161.2:c.351G>A	NM_003161.2	c.351G>A	NP_003152.1	NM_003161.2:c.351G>A(p.=)
PPM1D-17-58677903-G>G/A	PPM1D	missense_variant	43	R/Q	NM_003620.3:c.128G>A	NM_003620.3	c.128G>A	NP_003611.1	NP_003611.1:p.Arg43Gln
CD79B-17-62009606-A>A/T	CD79B	missense_variant	6	L/M	NM_001039933.1:c.16T>A	NM_001039933.1	c.16T>A	NP_001035022.1	NP_001035022.1:p.Leu6Met
FAM20A-17-66596441-G>G/A	FAM20A	synonymous_variant	123	L	NM_017565.3:c.367C>T	NM_017565.3	c.367C>T	NP_060035.2	NM_017565.3:c.367C>T(p.=)
TMC6-17-76116917-C>C/T	TMC6	intron_variant	0	NA	NM_007267.6:c.1536-4G>A	NM_007267.6	c.1536-4G>A	NP_009198.4	NA
RNF213-17-78282833-C>C/T	RNF213	synonymous_variant	839	A	NM_001256071.1:c.2517C>T	NM_001256071.1	c.2517C>T	NP_001243000.1	NM_001256071.1:c.2517C>T(p.=)
WDR45B-17-80606200-C>C/T	WDR45B	missense_variant	6	C/Y	NM_019613.3:c.17G>A	NM_019613.3	c.17G>A	NP_062559.2	NP_062559.2:p.Cys6Tyr
CIDEA-18-12274200-G>G/A	CIDEA	missense_variant	147	V/M	NM_001279.3:c.439G>A	NM_001279.3	c.439G>A	NP_001270.1	NP_001270.1:p.Val147Met
NPC1-18-21124914-C>C/G	NPC1	intron_variant	0	NA	NM_000271.4:c.1947+10G>C	NM_000271.4	c.1947+10G>C	NP_000262.2	NA
SETBP1-18-42456670-C>C/CTCTT	SETBP1	intron_variant	0	NA	NM_015559.2:c.540+7422_540+7423insTCTT	NM_015559.2	c.540+7422_540+7423insTCTT	NP_056374.2	NA
SETBP1-18-42531303-C>C/T	SETBP1	synonymous_variant	666	I	NM_015559.2:c.1998C>T	NM_015559.2	c.1998C>T	NP_056374.2	NM_015559.2:c.1998C>T(p.=)
MYO5B-18-47379818-C>C/T	MYO5B	splice_variant	0	NA	NM_001080467.2:c.4221+1G>A	NM_001080467.2	c.4221+1G>A	NP_001073936.1	NA
MYO5B-18-47421410-C>C/T	MYO5B	synonymous_variant	982	Q	NM_001080467.2:c.2946G>A	NM_001080467.2	c.2946G>A	NP_001073936.1	NM_001080467.2:c.2946G>A(p.=)
SERPINB5-18-61170790-A>A/G	SERPINB5	synonymous_variant	321	K	NM_002639.4:c.963A>G	NM_002639.4	c.963A>G	NP_002630.2	NM_002639.4:c.963A>G(p.=)

STK11-19-1221211-G>G/A	STK11	splice_variant	0	NA	NM_000455.4:c.735-1G>A	NM_000455.4	c.735-1G>A	NP_000446.1	NA
ICAM1-19-10385601-G>G/A	ICAM1	synonymous_variant	76	R	NM_000201.2:c.228G>A	NM_000201.2	c.228G>A	NP_000192.2	NM_000201.2:c.228G>A(p.=)
TYK2-19-10464843-G>G/A	TYK2	missense_variant	928	A/V	NM_003331.4:c.2783C>T	NM_003331.4	c.2783C>T	NP_003322.3	NP_003322.3:p.Ala928Val
CYP4F2-19-15989696-G>G/C	CYP4F2	missense_variant	483	A/G	NM_001082.3:c.1448C>G	NM_001082.3	c.1448C>G	NP_001073.3	NP_001073.3:p.Ala483Gly
CYP4F2-19-15989730-T>T/C	CYP4F2	missense_variant	472	T/A	NM_001082.3:c.1414A>G	NM_001082.3	c.1414A>G	NP_001073.3	NP_001073.3:p.Thr472Ala
GPI-19-34855843-T>T/C	GPI	missense_variant	10	L/P	NM_001184722.1:c.29T>C	NM_001184722.1	c.29T>C	NP_001171651.1	NP_001171651.1:p.Leu10Pro
COX7A1-19-36642562-A>A/C	COX7A1	intron_variant	0	NA	NM_001864.2:c.102+9T>G	NM_001864.2	c.102+9T>G	NP_001855.1	NA
LTBP4-19-41123093-A>AG/AG	LTBP4	frameshift_variant	1078	NA	NM_001042544.1:c.3233_3234insG	NM_001042544.1	c.3233_3234insG	NP_001036009.1	NP_001036009.1:p.Val1079GlyfsTer547
CD177-19-43860192-T>G/G	CD177	missense_variant	184	V/G	NM_020406.2:c.551T>G	NM_020406.2	c.551T>G	NP_065139.2	NP_065139.2:p.Val184Gly
CD177-19-43865692-G>G/A	CD177	missense_variant	348	A/T	NM_020406.2:c.1042G>A	NM_020406.2	c.1042G>A	NP_065139.2	NP_065139.2:p.Ala348Thr
FKRP-19-47258956-C>C/T	FKRP	synonymous_variant	83	A	NM_001039885.2:c.249C>T	NM_001039885.2	c.249C>T	NP_001034974.1	NM_001039885.2:c.249C>T(p.=)
LHB-19-49519883-A>A/G	LHB	missense_variant	35	I/T	NM_000894.2:c.104T>C	NM_000894.2	c.104T>C	NP_000885.1	NP_000885.1:p.Ile35Thr
HRC-19-49657889-T>T/TTCC	HRC	inframe	202	E/EE	NM_002152.2:c.603_605dupGGA	NM_002152.2	c.603_605dupGGA	NP_002143.1	NP_002143.1:p.Glu201dup
CLEC11A-19-51228591-C>C/T	CLEC11A	missense_variant	280	P/L	NM_002975.2:c.839C>T	NM_002975.2	c.839C>T	NP_002966.1	NP_002966.1:p.Pro280Leu
SIGLEC12-19-51995034-G>G/A	SIGLEC12	missense_variant	550	P/L	NM_053003.2:c.1649C>T	NM_053003.2	c.1649C>T	NP_443729.1	NP_443729.1:p.Pro550Leu
SIGLEC12-19-52004795-G>G/T	SIGLEC12	synonymous_variant	65	R	NM_053003.2:c.193C>A	NM_053003.2	c.193C>A	NP_443729.1	NM_053003.2:c.193C>A(p.=)
KIR2DL3-19-55250036-T>T/C	KIR2DL3	missense_variant	9	V/A	NM_015868.2:c.26T>C	NM_015868.2	c.26T>C	NP_056952.2	NP_056952.2:p.Val9Ala
KIR2DL3-19-55250979-C>C/A	KIR2DL3	missense_variant	21	P/T	NM_015868.2:c.61C>A	NM_015868.2	c.61C>A	NP_056952.2	NP_056952.2:p.Pro21Thr
KIR2DL3-19-55253465-C>C/G	KIR2DL3	missense_variant	37	P/R	NM_015868.2:c.110C>G	NM_015868.2	c.110C>G	NP_056952.2	NP_056952.2:p.Pro37Arg
KIR2DL3-19-55253521-C>C/G	KIR2DL3	missense_variant	56	Q/E	NM_015868.2:c.166C>G	NM_015868.2	c.166C>G	NP_056952.2	NP_056952.2:p.Gln56Glu
KIR2DL3-19-55253544-A>A/G	KIR2DL3	synonymous_variant	63	E	NM_015868.2:c.189A>G	NM_015868.2	c.189A>G	NP_056952.2	NM_015868.2:c.189A>G(p.=)
KIR2DL3-19-55253601-G>G/A	KIR2DL3	synonymous_variant	82	K	NM_015868.2:c.246G>A	NM_015868.2	c.246G>A	NP_056952.2	NM_015868.2:c.246G>A(p.=)
KIR2DL3-19-55258830-C>C/T	KIR2DL3	synonymous_variant	236	S	NM_015868.2:c.708C>T	NM_015868.2	c.708C>T	NP_056952.2	NM_015868.2:c.708C>T(p.=)
KIR2DL3-19-55258831-G>G/A	KIR2DL3	missense_variant	237	E/K	NM_015868.2:c.709G>A	NM_015868.2	c.709G>A	NP_056952.2	NP_056952.2:p.Glu237Lys
KIR2DL1-19-55284787-G>G/T	KIR2DL1	missense_variant	25	V/F	NM_014218.2:c.73G>T	NM_014218.2	c.73G>T	NP_055033.2	NP_055033.2:p.Val25Phe
KIR2DL1-19-55284791-A>A/G	KIR2DL1	missense_variant	26	H/R	NM_014218.2:c.77A>G	NM_014218.2	c.77A>G	NP_055033.2	NP_055033.2:p.His26Arg
KIR2DL1-19-55284858-G>G/A	KIR2DL1	synonymous_variant	48	Q	NM_014218.2:c.144G>A	NM_014218.2	c.144G>A	NP_055033.2	NM_014218.2:c.144G>A(p.=)
KIR2DL1-19-55284882-A>A/G	KIR2DL1	synonymous_variant	56	E	NM_014218.2:c.168A>G	NM_014218.2	c.168A>G	NP_055033.2	NM_014218.2:c.168A>G(p.=)
KIR2DL1-19-55285045-G>G/T	KIR2DL1	missense_variant	111	V/L	NM_014218.2:c.331G>T	NM_014218.2	c.331G>T	NP_055033.2	NP_055033.2:p.Val111Leu
KIR2DL1-19-55286769-C>C/A	KIR2DL1	missense_variant	175	P/T	NM_014218.2:c.523C>A	NM_014218.2	c.523C>A	NP_055033.2	NP_055033.2:p.Pro175Thr
KIR2DL1-19-55286796-G>G/A	KIR2DL1	missense_variant	184	D/N	NM_014218.2:c.550G>A	NM_014218.2	c.550G>A	NP_055033.2	NP_055033.2:p.Asp184Asn
KIR2DL1-19-55286822-C>C/T	KIR2DL1	synonymous_variant	192	H	NM_014218.2:c.576C>T	NM_014218.2	c.576C>T	NP_055033.2	NM_014218.2:c.576C>T(p.=)
KIR2DL1-19-55286828-G>G/A	KIR2DL1	synonymous_variant	194	G	NM_014218.2:c.582G>A	NM_014218.2	c.582G>A	NP_055033.2	NM_014218.2:c.582G>A(p.=)
KIR2DL1-19-55286854-A>A/G	KIR2DL1	missense_variant	203	H/R	NM_014218.2:c.608A>G	NM_014218.2	c.608A>G	NP_055033.2	NP_055033.2:p.His203Arg
KIR2DL1-19-55286864-A>A/C	KIR2DL1	synonymous_variant	206	P	NM_014218.2:c.618A>C	NM_014218.2	c.618A>C	NP_055033.2	NM_014218.2:c.618A>C(p.=)
KIR2DL1-19-55294454-C>C/T	KIR2DL1	missense_variant	266	R/C	NM_014218.2:c.796C>T	NM_014218.2	c.796C>T	NP_055033.2	NP_055033.2:p.Arg266Cys
KIR2DL1-19-55294484-T>C/C	KIR2DL1	intron_variant	0	NA	NM_014218.2:c.817+9T>C	NM_014218.2	c.817+9T>C	NP_055033.2	NA
KIR2DL1-19-55294931-T>T/C	KIR2DL1	intron_variant	0	NA	NM_014218.2:c.818-7T>C	NM_014218.2	c.818-7T>C	NP_055033.2	NA
KIR2DL1-19-55294969-A>A/G	KIR2DL1	synonymous_variant	283	G	NM_014218.2:c.849A>G	NM_014218.2	c.849A>G	NP_055033.2	NM_014218.2:c.849A>G(p.=)
KIR2DL1-19-55295215-A>A/G	KIR2DL1	missense_variant	333	T/A	NM_014218.2:c.997A>G	NM_014218.2	c.997A>G	NP_055033.2	NP_055033.2:p.Thr333Ala
KIR2DL4-19-55316329-A>A/G	KIR2DL4	missense_variant	53	Y/C	NM_001080770.1:c.158A>G	NM_001080770.1	c.158A>G	NP_001074239.1	NP_001074239.1:p.Tyr53Cys
KIR2DL4-19-55317524-G>G/A	KIR2DL4	synonymous_variant	160	E	NM_001080770.1:c.480G>A	NM_001080770.1	c.480G>A	NP_001074239.1	NM_001080770.1:c.480G>A(p.=)
KIR2DL4-19-55320279-G>A/A	KIR2DL4	intron_variant	0	NA	NM_001080770.1:c.656-9G>A	NM_001080770.1	c.656-9G>A	NP_001074239.1	NA
KIR3DL1-19-55327960-C>C/T	KIR3DL1	missense_variant	2	S/L	NM_013289.2:c.5C>T	NM_013289.2	c.5C>T	NP_037421.2	NP_037421.2:p.Ser2Leu
KIR3DL1-19-55329783-A>A/G	KIR3DL1	synonymous_variant	28	K	NM_013289.2:c.84A>G	NM_013289.2	c.84A>G	NP_037421.2	NM_013289.2:c.84A>G(p.=)
KIR3DL1-19-55329822-A>A/C	KIR3DL1	synonymous_variant	41	R	NM_013289.2:c.123A>C	NM_013289.2	c.123A>C	NP_037421.2	NM_013289.2:c.123A>C(p.=)

KIR3DL1-19-55329901-A>A/G	KIR3DL1	missense_variant	68	I/V	NM_013289.2:c.202A>G	NM_013289.2	c.202A>G	NP_037421.2	NP_037421.2:p.Ile68Val
KIR3DL1-19-55329934-A>A/G	KIR3DL1	missense_variant	79	S/G	NM_013289.2:c.235A>G	NM_013289.2	c.235A>G	NP_037421.2	NP_037421.2:p.Ser79Gly
KIR3DL1-19-55330036-G>G/A	KIR3DL1	missense_variant	113	V/M	NM_013289.2:c.337G>A	NM_013289.2	c.337G>A	NP_037421.2	NP_037421.2:p.Val113Met
KIR3DL1-19-55333023-C>C/T	KIR3DL1	missense_variant	220	P/L	NM_013289.2:c.659C>T	NM_013289.2	c.659C>T	NP_037421.2	NP_037421.2:p.Pro220Leu
KIR3DL1-19-55333246-C>C/T	KIR3DL1	synonymous_variant	294	F	NM_013289.2:c.882C>T	NM_013289.2	c.882C>T	NP_037421.2	NM_013289.2:c.882C>T(p.=)
KIR3DL1-19-55333275-G>G/T	KIR3DL1	missense_variant	304	W/L	NM_013289.2:c.911G>T	NM_013289.2	c.911G>T	NP_037421.2	NP_037421.2:p.Trp304Leu
KIR3DL2-19-55361942-G>G/T	KIR3DL2	synonymous_variant	4	T	NM_006737.3:c.12G>T	NM_006737.3	c.12G>T	NP_006728.2	NM_006737.3:c.12G>T(p.=)
KIR3DL2-19-55363704-G>G/A	KIR3DL2	missense_variant	108	A/T	NM_006737.3:c.322G>A	NM_006737.3	c.322G>A	NP_006728.2	NP_006728.2:p.Ala108Thr
FCAR-19-55399537-G>G/A	FCAR	synonymous_variant	175	P	NM_002000.2:c.525G>A	NM_002000.2	c.525G>A	NP_001991.1	NM_002000.2:c.525G>A(p.=)
NLRP7-19-55451116-G>G/A	NLRP7	synonymous_variant	357	F	NM_001127255.1:c.1071C>T	NM_001127255.1	c.1071C>T	NP_001120727.1	NM_001127255.1:c.1071C>T(p.=)
GP6-19-55526249-G>G/A	GP6	missense_variant	355	S/L	NM_001083899.1:c.1064C>T	NM_001083899.1	c.1064C>T	NP_001077368.1	NP_001077368.1:p.Ser355Leu
SLC27A5-19-59021378-G>G/A	SLC27A5	intron_variant	0	NA	NM_012254.2:c.899-6C>T	NM_012254.2	c.899-6C>T	NP_036386.1	NA
RSPO4-20-944659-G>G/T	RSPO4	synonymous_variant	172	R	NM_001029871.3:c.514C>A	NM_001029871.3	c.514C>A	NP_001025042.2	NM_001029871.3:c.514C>A(p.=)
TGM6-20-2411240-C>C/T	TGM6	synonymous_variant	609	T	NM_198994.2:c.1827C>T	NM_198994.2	c.1827C>T	NP_945345.2	NM_198994.2:c.1827C>T(p.=)
MAVS-20-3845296-C>C/T	MAVS	missense_variant	340	A/V	NM_020746.4:c.1019C>T	NM_020746.4	c.1019C>T	NP_065797.2	NP_065797.2:p.Ala340Val
PLCB4-20-9457409-G>G/A	PLCB4	intron_variant	0	NA	NM_000933.3:c.3496+9G>A	NM_000933.3	c.3496+9G>A	NP_000924.3	NA
RPN2-20-35856945-C>C/T	RPN2	intron_variant	0	NA	NM_002951.3:c.1300-8C>T	NM_002951.3	c.1300-8C>T	NP_002942.2	NA
CHD6-20-40033494-G>G/A	CHD6	synonymous_variant	2629	S	NM_032221.3:c.7887C>T	NM_032221.3	c.7887C>T	NP_115597.3	NM_032221.3:c.7887C>T(p.=)
CHD6-20-40033495-G>G/A	CHD6	missense_variant	2629	S/F	NM_032221.3:c.7886C>T	NM_032221.3	c.7886C>T	NP_115597.3	NP_115597.3:p.Ser2629Phe
PTPRT-20-40790158-G>G/A	PTPRT	missense_variant	858	S/F	NM_133170.3:c.2573C>T	NM_133170.3	c.2573C>T	NP_573400.3	NP_573400.3:p.Ser858Phe
ADNP-20-49508776-C>C/A	ADNP	synonymous_variant	825	G	NM_015339.2:c.2475G>T	NM_015339.2	c.2475G>T	NP_056154.1	NM_015339.2:c.2475G>T(p.=)
CYP24A1-20-52774674-C>C/T	CYP24A1	missense_variant	396	R/Q	NM_000782.4:c.1187G>A	NM_000782.4	c.1187G>A	NP_000773.2	NP_000773.2:p.Arg396Gln
VAPB-20-56964483-C>C/G	VAPB	5_prime_UTR_variant	0	NA	NM_004738.4:c.-33C>G	NM_004738.4	c.-33C>G	NP_004729.1	NA
GNAS-20-57464277-C>CCGGCG/CCGGCG	GNAS	intron_variant	0	NA	NM_080425.2:c.2069-6390_2069-6389insCGGCG	NM_080425.2	c.2069-6390_2069-6389insCGGCG	NP_536350.2	NA
LAMA5-20-60886124-G>G/A	LAMA5	missense_variant	3372	P/L	NM_005560.3:c.10115C>T	NM_005560.3	c.10115C>T	NP_005551.3	NP_005551.3:p.Pro3372Leu
LAMA5-20-60902379-G>G/A	LAMA5	synonymous_variant	1674	L	NM_005560.3:c.5022C>T	NM_005560.3	c.5022C>T	NP_005551.3	NM_005560.3:c.5022C>T(p.=)
LAMA5-20-60942256-G>G/A	LAMA5	missense_variant	16	P/S	NM_005560.3:c.46C>T	NM_005560.3	c.46C>T	NP_005551.3	NP_005551.3:p.Pro16Ser
TPTE-21-10935004-A>A/T	TPTE	intron_variant	0	NA	NM_199261.2:c.796-7T>A	NM_199261.2	c.796-7T>A	NP_954870.2	NA
TPTE-21-10942756-G>G/A	TPTE	stop_gained	229	R/*	NM_199261.2:c.685C>T	NM_199261.2	c.685C>T	NP_954870.2	NP_954870.2:p.Arg229Ter
TPTE-21-10943003-C>C/T	TPTE	missense_variant	195	R/Q	NM_199261.2:c.584G>A	NM_199261.2	c.584G>A	NP_954870.2	NP_954870.2:p.Arg195Gln
TMPRSS15-21-19642394-G>G/A	TMPRSS15	synonymous_variant	984	F	NM_002772.2:c.2952C>T	NM_002772.2	c.2952C>T	NP_002763.2	NM_002772.2:c.2952C>T(p.=)
APP-21-27327970-G>G/A	APP	missense_variant	520	P/S	NM_000484.3:c.1558C>T	NM_000484.3	c.1558C>T	NP_000475.1	NP_000475.1:p.Pro520Ser
GRIK1-21-31045476-G>G/A	GRIK1	missense_variant	185	R/C	NM_000830.3:c.553C>T	NM_000830.3	c.553C>T	NP_000821.1	NP_000821.1:p.Arg185Cys
TRPM2-21-45817705-G>G/A	TRPM2	missense_variant	670	D/N	NM_003307.3:c.2008G>A	NM_003307.3	c.2008G>A	NP_003298.1	NP_003298.1:p.Asp670Asn
TRPM2-21-45821681-C>C/T	TRPM2	synonymous_variant	813	F	NM_003307.3:c.2439C>T	NM_003307.3	c.2439C>T	NP_003298.1	NM_003307.3:c.2439C>T(p.=)
COL6A2-21-47533977-G>G/A	COL6A2	missense_variant	264	R/H	NM_001849.3:c.791G>A	NM_001849.3	c.791G>A	NP_001840.3	NP_001840.3:p.Arg264His
PCNT-21-47754449-G>G/A	PCNT	missense_variant	136	G/S	NM_006031.5:c.406G>A	NM_006031.5	c.406G>A	NP_006022.3	NP_006022.3:p.Gly136Ser
PCNT-21-47754471-G>G/A	PCNT	missense_variant	143	R/H	NM_006031.5:c.428G>A	NM_006031.5	c.428G>A	NP_006022.3	NP_006022.3:p.Arg143His
CLTCL1-22-19189003-A>AC/AC	CLTCL1	frameshift_variant	1201	NA	NM_007098.3:c.3601dupG	NM_007098.3	c.3601dupG	NP_009029.3	NP_009029.3:p.Val1201GlyfsTer19
CLTCL1-22-19213835-C>C/T	CLTCL1	synonymous_variant	618	E	NM_007098.3:c.1854G>A	NM_007098.3	c.1854G>A	NP_009029.3	NM_007098.3:c.1854G>A(p.=)

TXNRD2-22-19883063-G>G/A	TXNRD2	synonymous_variant	272	G	NM_006440.3:c.816C>T	NM_006440.3	c.816C>T	NP_006431.2	NM_006440.3:c.816C>T(p.=)
COMT-22-19956262-G>GC/GC	COMT	3_prime_UTR_variant	0	NA	NM_000754.3:c.*3_*4insC	NM_000754.3	c.*3_*4insC	NP_000745.1	NA
BCR-22-23651670-G>G/A	BCR	synonymous_variant	1024	G	NM_004327.3:c.3072G>A	NM_004327.3	c.3072G>A	NP_004318.3	NM_004327.3:c.3072G>A(p.=)
BCR-22-23651671-G>G/A	BCR	splice_variant	0	NA	NM_004327.3:c.3072+1G>A	NM_004327.3	c.3072+1G>A	NP_004318.3	NA
GGT5-22-24616028-G>G/A	GGT5	synonymous_variant	558	F	NM_001099781.1:c.1674C>T	NM_001099781.1	c.1674C>T	NP_001093251.1	NM_001099781.1:c.1674C>T(p.=)
GGT5-22-24629461-G>G/A	GGT5	intron_variant	0	NA	NM_001099781.1:c.400+5C>T	NM_001099781.1	c.400+5C>T	NP_001093251.1	NA
ADORA2A.ADORA2A-AS1-22-24837124-C>C/T	ADORA2A.ADORA2A-AS1	intron_variant	0	NA	NR_028484.1:n.574+836G>A	NR_028484.1	n.574+836G>A	NA	NA
MYO18B-22-26164462-G>G/A	MYO18B	synonymous_variant	193	G	NM_032608.5:c.579G>A	NM_032608.5	c.579G>A	NP_115997.5	NM_032608.5:c.579G>A(p.=)
NEFH-22-29885686-C>C/A	NEFH	missense_variant	686	A/E	NM_021076.3:c.2057C>A	NM_021076.3	c.2057C>A	NP_066554.2	NP_066554.2:p.Ala686Glu
NCF4-22-37261097-C>C/A	NCF4	missense_variant	85	T/N	NM_013416.3:c.254C>A	NM_013416.3	c.254C>A	NP_038202.2	NP_038202.2:p.Thr85Asn
TRIOBP-22-38120041-G>G/A	TRIOBP	missense_variant	493	S/N	NM_001039141.2:c.1478G>A	NM_001039141.2	c.1478G>A	NP_001034230.1	NP_001034230.1:p.Ser493Asn
TRIOBP-22-38120503-G>G/A	TRIOBP	missense_variant	647	S/N	NM_001039141.2:c.1940G>A	NM_001039141.2	c.1940G>A	NP_001034230.1	NP_001034230.1:p.Ser647Asn
APOBEC3B-22-39387400-C>C/T	APOBEC3B	missense_variant	263	P/S	NM_004900.4:c.787C>T	NM_004900.4	c.787C>T	NP_004891.4	NP_004891.4:p.Pro263Ser
SYNGR1-22-39777822-C>C/CCAA	SYNGR1	inframe	202	P/PN	NM_004711.4:c.605_606insCAA	NM_004711.4	c.605_606insCAA	NP_004702.2	NP_004702.2:p.Pro202_Thr203insAsn
NDUFA6-22-42482228-T>T/C	NDUFA6	missense_variant	142	K/E	NM_002490.3:c.424A>G	NM_002490.3	c.424A>G	NP_002481.2	NP_002481.2:p.Lys142Glu
VCX3A-X-6451778-C>C/T	VCX3A	3_prime_UTR_variant	0	NA	NM_016379.3:c.*8G>A	NM_016379.3	c.*8G>A	NP_057463.2	NA
PHKA2-X-18936859-T>C/C	PHKA2	missense_variant	693	I/V	NM_000292.2:c.2077A>G	NM_000292.2	c.2077A>G	NP_000283.1	NP_000283.1:p.Ile693Val
SYTL5-X-37961637-T>C/C	SYTL5	missense_variant	369	S/P	NM_001163334.1:c.1105T>C	NM_001163334.1	c.1105T>C	NP_001156806.1	NP_001156806.1:p.Ser369Pro
IQSEC2-X-53279792-G>G/A	IQSEC2	missense_variant	656	P/S	NM_001111125.2:c.1966C>T	NM_001111125.2	c.1966C>T	NP_001104595.1	NP_001104595.1:p.Pro656Ser
EDA2R-X-65835841-A>G/G	EDA2R	missense_variant	8	Y/H	NM_001242310.1:c.22T>C	NM_001242310.1	c.22T>C	NP_001229239.1	NP_001229239.1:p.Tyr8His
TSIX.XIST-X-73042393-T>T/C	TSIX.XIST	nc_exon_variant	0	NA	NR_003255.2:n.30354T>C	NR_003255.2	n.30354T>C	NA	NA
ZDHHC15-X-74649010-G>G/A	ZDHHC15	missense_variant	169	S/F	NM_144969.2:c.506C>T	NM_144969.2	c.506C>T	NP_659406.1	NP_659406.1:p.Ser169Phe
IRS4-X-107977804-C>T/T	IRS4	missense_variant	591	G/S	NM_003604.2:c.1771G>A	NM_003604.2	c.1771G>A	NP_003595.1	NP_003595.1:p.Gly591Ser
TRPC5-X-111024424-G>A/A	TRPC5	missense_variant	704	A/V	NM_012471.2:c.2111C>T	NM_012471.2	c.2111C>T	NP_036603.1	NP_036603.1:p.Ala704Val
GRIA3-X-122318386-A>AG/AG	GRIA3	5_prime_UTR_variant	0	NA	NM_007325.4:c.-2_-1insG	NM_007325.4	c.-2_-1insG	NP_015564.4	NA
GRIA3-X-122538605-G>G/A	GRIA3	missense_variant	447	G/E	NM_007325.4:c.1340G>A	NM_007325.4	c.1340G>A	NP_015564.4	NP_015564.4:p.Gly447Glu
GRIA3-X-122616676-C>C/A	GRIA3	missense_variant	822	S/R	NM_007325.4:c.2466C>A	NM_007325.4	c.2466C>A	NP_015564.4	NP_015564.4:p.Ser822Arg
XIAP-X-123047668-G>G/C	XIAP	3_prime_UTR_variant	0	NA	NM_001204401.1:c.*6637G>C	NM_001204401.1	c.*6637G>C	NP_001191330.1	NA
XIAP-X-123047728-T>T/C	XIAP	3_prime_UTR_variant	0	NA	NM_001204401.1:c.*6697T>C	NM_001204401.1	c.*6697T>C	NP_001191330.1	NA
SLC6A8-X-152954025-A>G/G	SLC6A8	5_prime_UTR_variant	0	NA	NM_005629.3:c.-5A>G	NM_005629.3	c.-5A>G	NP_005620.1	NA

Table S4

Gene	Chr	Position	HGVSc_nomenclature	Consequence	Allele Frequency				
					PR	P9	P60	Mean	St.Dev
EDA2R	X	65835841	NM_001242310.1:c.22T>C	missense_variant	100	100	100	100.0	0.0
HLA-DQB1	6	32632781	NM_001243961.1:c.173T>A	missense_variant	100	100	100	100.0	0.0
IRS4	X	107977804	NM_003604.2:c.1771G>A	missense_variant	100	100	100	100.0	0.0
PHKA2	X	18936859	NM_000292.2:c.2077A>G	missense_variant	100	100	100	100.0	0.0
TMEM216	11	61165741	NM_001173991.2:c.440G>C	missense_variant	100	100	100	100.0	0.0
TRPC5	X	111024424	NM_012471.2:c.2111C>T	missense_variant	100	100	100	100.0	0.0
UNC93B1	11	67765163	NM_030930.2:c.887dupC	frameshift_variant	100	100	100	100.0	0.0
PCNT	21	47754449	NM_006031.5:c.406G>A	missense_variant	26.4	25.6	25.9	26.0	0.4
ZNF804A	2	185801756	NM_194250.1:c.1633G>A	missense_variant	30.4	29.8	30.8	30.3	0.5
PRB3	12	11420333	NM_006249.4:c.723delC	frameshift_variant	89.1	90	89.1	89.4	0.5
ATR	3	142274770	NM_001184.3:c.2290A>G	missense_variant	49.2	49.3	48.3	48.9	0.6
TPRN	9	140093903	NM_001128228.2:c.1261C>T	missense_variant	52.9	52.6	53.7	53.1	0.6
NEFH	22	29885686	NM_021076.3:c.2057C>A	missense_variant	23	22.5	21.8	22.4	0.6
NCF1	7	74193668	NM_000265.4:c.295G>A	missense_variant	100	98.7	100	99.6	0.8
ANXA11	10	81926659	NM_001157.2:c.731C>T	missense_variant	62.9	61.6	61.5	62.0	0.8
LTBP4	19	41123093	NM_001042544.1:c.3233_3234insG	frameshift_variant	99.3	97.5	98.5	98.4	0.9
EMG1	12	7080210	NM_006331.7:c.124_125insG	frameshift_variant	99.5	97.7	98.9	98.7	0.9
SCARB1	12	125299559	NM_005505.4:c.386C>T	missense_variant	57.3	55.4	56.3	56.3	1.0
HLA-DQB1	6	33052736	NM_002121.5:c.374G>A	missense_variant	100	98.8	98	98.9	1.0
APOB	2	21260123	NM_000384.2:c.542C>T	missense_variant	27.3	28.1	29.4	28.3	1.1
DNASE1L3	3	58196522	NM_004944.3:c.112G>C	missense_variant	51.4	49	50.7	50.4	1.2
ZFHX4	8	77764310	NM_024721.4:c.5153T>C	missense_variant	51.1	49.7	48.4	49.7	1.4
TAF2	8	120816184	NM_003184.3:c.494G>A	missense_variant	41.3	44.8	44.6	43.6	2.0
RANBP2	2	109383674	NM_006267.4:c.6679T>C	missense_variant	38.6	35.4	34.8	36.3	2.0
ABHD5	3	43743914	NM_016006.4:c.341G>T	missense_variant	51.2	49.3	53.4	51.3	2.1
MYO7B	2	128351149	NM_001080527.1:c.2174T>G	missense_variant	70	69.4	73.3	70.9	2.1
MMP12	11	102738793	NM_002426.4:c.631dupA	frameshift_variant	96	100	100	98.7	2.3
PCNT	21	47754471	NM_006031.5:c.428G>A	missense_variant	30.3	30.9	26.1	29.1	2.6

TOPBP1	3	133320222	NM_007027.3:c.4441G>A	missense_variant	47.3	52.7	49.4	49.8	2.7
FRG1	4	190883026	NM_004477.2:c.679G>T	missense_variant	25	21.5	27.3	24.6	2.9
AKAP13	15	86265465	NM_006738.4:c.6395A>T	missense_variant	50	45.5	51.2	48.9	3.0
KIR2DL3	19	55250979	NM_015868.2:c.61C>A	missense_variant	28.2	25.6	22.2	25.3	3.0
TPTE	21	10943003	NM_199261.2:c.584G>A	missense_variant	29.2	30.9	35.2	31.8	3.1
SRGAP2	1	206566904	NM_015326.2:c.448T>C	missense_variant.	36.4	42.5	41.1	40.0	3.2
FMN2	1	240370907	NM_020066.4:c.2795T>C	missense_variant	42.9	46.2	39.5	42.9	3.4
HYDIN	16	71122408	NM_032821.2:c.1466G>A	missense_variant	38.1	42.9	44.6	41.9	3.4
GTF2IRD2	7	74212036	NM_173537.2:c.1815G>C	missense_variant	42.2	48.6	43.2	44.7	3.4
PEX19	1	160254899	NM_002857.3:c.16G>A	missense_variant	42.2	46.2	39.3	42.6	3.5
TSC2	16	2134508	NM_000548.3:c.4285G>T	missense_variant	54.5	48.1	53.6	52.1	3.5
CLTCL1	22	19189003	NM_007098.3:c.3601dupG	frameshift_variant	91.3	93.9	98.6	94.6	3.7
NINJ1	9	95888872	NM_004148.3:c.124G>A	missense_variant	49.7	53.7	45.9	49.8	3.9
PMS2	7	6036969	NM_000535.5:c.791A>G	missense_variant	35	42.7	37.5	38.4	3.9
KIR2DL3	19	55250036	NM_015868.2:c.26T>C	missense_variant	62.2	62.2	55.3	59.9	4.0
HLA-A	6	29912856	NM_002116.7:c.1033A>T	missense_variant	43.2	49.3	51	47.8	4.1
COL7A1	3	48618079	NM_000094.3:c.4987C>A	missense_variant	52.7	54.6	60.7	56.0	4.2
ACSM3.ERI2	16	20792417	NM_005622.3:c.904C>T	missense_variant	48.1	52.8	44.4	48.4	4.2
KIR3DL1	19	55327960	NM_013289.2:c.5C>T	missense_variant	42	34	40.9	39.0	4.3
NPSR1	7	34818119	NM_207173.1:c.326G>A	missense_variant	42	45.3	50.7	46.0	4.4
JAG2	14	105609822	NM_002226.4:c.3238A>G	missense_variant	30.2	36.6	38.9	35.2	4.5
HYDIN	16	70917931	NM_032821.2:c.9868G>C	missense_variant	51.1	43.6	42.9	45.9	4.5
ABCC3	17	48753045	NM_003786.3:c.2768G>A	missense_variant	55.2	50.9	46	50.7	4.6
TG	8	133899575	NM_003235.4:c.1958G>A	missense_variant	44.5	48.8	53.8	49.0	4.7
KIR3DL1	19	55329901	NM_013289.2:c.202A>G	missense_variant	52.1	46	42.6	46.9	4.8
HYDIN	16	70897039	NM_032821.2:c.11515G>C	missense_variant	43.3	47.4	37.8	42.8	4.8
TPSB2	16	1278767	NM_024164.5:c.712G>A	missense_variant	38.6	37.9	29.7	35.4	4.9

Table S5
